# Insights into In Vitro Adaptation of EV71 and Analysis of Reduced Virulence by In Silico Predictions

**DOI:** 10.3390/vaccines11030629

**Published:** 2023-03-11

**Authors:** Jia Xuen Koh, Malihe Masomian, Mohd Ishtiaq Anasir, Seng-Kai Ong, Chit Laa Poh

**Affiliations:** 1 Centre for Virus and Vaccine Research, School of Medical and Life Sciences, Sunway University, Bandar Sunway, Petaling Jaya 47500, Selangor, Malaysia; 2 Research and Development Department, Pure Biologics SA, Duńska 11, 54-427 Wroclaw, Poland; 3 Virology Unit, Infectious Disease Research Center, Institute for Medical Research, National Institutes of Health, Ministry of Health Malaysia, Shah Alam 40170, Selangor, Malaysia; 4 Department of Biological Sciences, School of Medical and Life Sciences, Sunway University, Bandar Sunway, Petaling Jaya 47500, Selangor, Malaysia

**Keywords:** enterovirus 71, quasispecies, spontaneous mutations, in vitro virulence, structural analysis, next-generation sequencing

## Abstract

EV-A71 is a common viral pathogen that causes hand, foot and mouth disease. It is a single-stranded RNA virus that has a low fidelity RNA polymerase and, as a result, spontaneous mutations frequently occur in the EV-A71 genome. The mutations within the genome give rise to quasispecies within the viral population that could be further defined by haplotypes. In vitro virulence of EV-A71 was shown by plaque size in Rhabdomyosarcoma (RD) cells, which was substantiated by in vitro characterizations of growth, RNA replication, binding, attachment and host cell internalization. Viruses could exhibit different host cell adaptations in different cell lines during viral passaging. The EV-A71/WT (derived from EV-A71 subgenotype B4) was shown to comprise six haplotypes through next-generation sequencing, where only EV-A71/Hap2 was found to be cultivable in RD cells, while EV-A71/Hap4 was the only cultivable haplotype in Vero cells. The EV-A71/WT produced plaques of four different sizes (small, medium, big, huge) in RD cells, while only two plaque variants (small, medium) were present in Vero cells. The small plaque variant isolated from RD cells displayed lower RNA replication rates, slower in vitro growth kinetics, higher TCID_50_ and lower attachment, binding and entry ability when compared against EV-A71/WT due to the mutation at 3D-S228P that disrupted the active site of the RNA polymerase, resulting in low replication and growth of the variant.

## 1. Introduction

Enterovirus 71 (EV-A71) is a non-enveloped positive-sense single-stranded RNA virus of approximately 7.4 kb in length, belonging to the genus *Enterovirus* of the *Picornaviridae* family. The RNA genome is flanked by 5′ and 3′ untranslated regions (UTR), followed by a polyadenylated tail [1]. The 5′ UTR contains six stem-loop structures (I-VI) that are involved in RNA replication and cap-independent viral protein translation that are controlled by the internal ribosome entry site (IRES) [2]. The large open reading frame (ORF) encodes a single polyprotein, which is further cleaved into P1, P2 and P3 regions by viral proteases [1]. The structural protein P1 region, consisting of capsid proteins viral protein 1-4 (VP1-VP4), is responsible for viral entry, attachment and delivery of the viral genome into host cells [3,4]. The P2 and P3 regions comprise the non-structural proteins 2A-2C and 3A-3D, respectively [3]. The 3D protein of EV-A71 is an RNA-dependent RNA polymerase (RdRp) controlling replication and is known to be error-prone, producing up to 10^−4^ to 10^−5^ mutations and leading to nucleotide changes in the genome due to its low fidelity [5]. Therefore, RNA polymerases could contribute to the emergence of diverse progenies with different genome compositions, referred to as quasispecies, which are mutants with genetically related genomes in a viral population [6].

Plaque sizes are indicative of viral replication rates, where small plaque variants have been reported to exhibit slow replication rates, leading to less spread and cell killing and could have lower in vitro virulence [7,8,9,10,11]. Small plaque variants of the Chikungunya virus, the Dengue virus, the Newcastle disease virus and the Zika virus with reduced growth rates in cell cultures were similarly reported [7,8,9,10]. Mandary et al. (2020) reported the presence of two plaque variants from the wild-type EV-A71 subgenotype B4 strain 41, namely the big and small plaque variants from Rhabdomyosarcoma (RD) cells. Both variants showed slower growth kinetics in terms of RNA production and infectivity when compared with the wild-type EV-A71. Genome analysis indicated several spontaneous mutations in both plaque variants; they were all located in the VP1 region, with the big plaque variant harboring four mutations at I97L, N104S, S246P and N282D and the small plaque variant carrying three mutations at I97T, N237T and T292A [12]. In the West Nile virus, a small plaque variant (SP) was isolated from a population displaying mixed plaque phenotypes; sequence analysis revealed four nucleotide changes [13]. Two of the changes resulted in amino acid substitutions (prM-P54S and NS2A-V61A) when compared with the wild type (WT). In vitro growth kinetics of the SP variant at incubation temperatures above 41 °C in the Vero and DF-1 chicken embryo fibroblasts and in vivo growth kinetics in mosquitoes after 2, 5 and 8 dpi at 32 °C, 27 °C and 15 °C, respectively, revealed the presence of mixed-plaque phenotypes, indicating reversion to the WT. The large plaque within the mixed-plaque phenotype was found to harbor the same nucleotide at position 625 (prM-54) as the WT. The minority of the large plaques contained the same nucleotide at position 3707 (NS2A-V61A) as the WT. This suggested that the amino acid substitution at either prM-54 or NS2A-61 might have contributed to small plaque formation [13]. There are a few exceptions where the small plaque variants were reported to have higher replication rates and virulence, more severe disease manifestations and were invasive in certain in vivo systems, e.g., in the case of the Japanese encephalitis virus, the Ross river virus and the Chikungunya virus [14,15,16]. The mutations observed in the plaque variants could be due to mutations already present in the various variants, referred to as haplotypes in the quasispecies population. However, the cultivation of viruses in specific cell lines could lead to the adaptation of the quasispecies by acquiring spontaneous mutations. EV-A71 subgenotype C2 propagated in Vero cells (EV-V) showed strong cell death and high virulence in mice [17]. However, EV-V propagated in RD cells produced EV-R, exhibited reduced virulence in mice and harbored mutations at 5′-UTR-U494C, VP1-E145G, VP1-V146I and VP1-S241L [17]. In the West Nile virus (WNV) and its close relative the St. Louis encephalitis virus (SLE), both viruses serially passaged in C6/36 mosquito cell lines showed enhanced relative fitness and replications when they were assessed for viral characteristics in a homologous system (C6/36 mosquito cell) [18]. Viral characteristics were also assessed in an alternate system by using DF-1 avian cells as the heterologous cell line, which differed from the cells used for serial passages, and both WNV and SLE did not show any signs of fitness gain or enhanced viral replication [18]. The genetic sequences of the WNV and SLE after serial passages in the C6/36 cells revealed a number of mutations that could contribute to cell adaptation of the mosquito cell lines, where three non-synonymous mutations identified in the NS4 gene of the SLE, namely U6720A (T to A), U6968A (V to E) and A7533C (I to L), were more likely to be cell-adapted mutations. These observations could provide evidence of cell-specific adaptations. However, the C6/36 cells were shown to have dysfunctional RNAi interference, which could explain high permissiveness of the cells towards arbovirus infections [19], resulting in increased replication and viral fitness. Nevertheless, these mutations should be reverted to the original sequence through site-directed mutagenesis techniques to evaluate the possibility of being cell-adapted mutations.

Some spontaneous mutations could serve as molecular determinants of virulence and studies have led to further understanding of the virus biology. For instance, Yee et al. (2016) reported that position 475 of the 5’-UTR of EV-A71 subgenotype B4 strain 41 might be a potential molecular determinant of virulence as it was shown that the mutation C475U had resulted in reduced cytopathic effects (CPE) and lower viral titers in RD cells [20]. The VP1 protein is a structural protein that was shown to play a role in viral binding towards cell receptors, such as SCARB2 [21]. The VP1 of the EV-A71 could correlate with viral tropism, such as VP1-145 G/Q that specifically interacted with leukocytes through the P-selectin glycoprotein ligand (PSGL-1), while VP1-145E was associated with the inability to bind [22]. Other mutations in VP1, such as VP1-244 and VP1-242, were found by Nishimura et al. (2013) to influence viral binding to PSGL-1. Depending on the amino acid being present at VP1-145, it could exert its influence to control the exposure of the side chains of VP1-244K and could act as a switch to control PSGL-1 binding [22]. Two mutations, K242A and K244A, were generated in a PSGL-1 binding strain (VP1-145G/Q) and the mutants containing K244A had reduced PSGL-1 binding, while double mutants exhibited no PSGL-1 binding [22]. Similarly, the VP1-L97R in the BC loop was reported to play a critical role in cell tropism and was demonstrated to affect viral binding ability and fitness in neuronal cells in vitro [23]. On the other hand, the 3D polymerase (3D^pol^) is known to be involved in the replication of the virus. It is an RNA-dependent-RNA polymerase (RdRp) which replicates infecting positive-sense genomes into minus-sense complements and uses these as templates for the synthesis of positive-sense genomes for translation and packaging into new virions [24]. It operates through the uridylation of VPg that is needed for RNA replication [25]. The L123F mutation in conjunction with G64R of the 3D^pol^ showed higher in vitro fidelity and 500 times higher LD_50_ in mice, indicating the presence of an attenuated pathogenic phenotype [26].

In silico studies, such as structural analysis, were used to gain an understanding of the effects of mutations in the virus on its infectivity and virulence at a molecular level. Through a cryoEM structure analysis of the EV-A71-SCARB2 binding complex, after substitution of alanine to threonine at VP1-280, it was found that VP1-280T, which formed a hydrogen bonding with VP2-139T, helped to strengthen the VP1-VP2 complex essential for the attachment and entry of EV-A71 towards a host SCARB2 receptor [27]. Through docking studies, VP1-K242 and K244 residues of EV-A71 were found to exhibit the highest interaction energy against a computer-simulated 12-mer heparin [28]. Mutagenesis of EV-A71 subgenotype B4 strain 41 was performed through alanine substitutions and mutants containing VP1-K242A and VP1-K244A yielded smaller foci and significant reductions in heparin binding [28].

Venkataraman et al. (2018) provided a thorough review on the domains and motifs of RNA polymerases [29]. The 3D^pol^ was made up of three domains, consisting of the thumb, palm and fingers region, and they were involved in RNA-template binding, nucleoside triphosphate (NTP) entry and polymerization. The active site cavity was encircled by the three domains and played a role in catalysis that facilitated viral replication. The palm region also contained entry channels in the front and at the back of the polymerase structure for RNA-primer binding and NTP entry, respectively. RdRps contained seven structural motifs (A–G) that had similar structures across various groups of viruses. Motif B functions as a hinge during template and substrate binding by inducing conformational changes in the polymerase for catalysis, such as repositioning of residues and changes in bond formation, with the assistance of nearby motif A and C [29]. A crystal structure of the 3D protein in the complex with its protein primer VPg was reported [30]. Residues 73, 80, 84, 87 and 311–343 formed the VPg-binding interface and it was shown that site 311 located at the base of the palm domain is the main VPg-binding region [25,30]. Alanine substitutions of T313, F314 and I317 caused a reduction of VPg uridylylation activity of more than 90% [25]. Alanine substitutions at position 319, 320 and 335 also caused significant defective VPg uridylylation, whereas F337A mutant impeded 50% reduced uridylylation activity and disrupted EV-A71 replication [30].

Generally, virus research focusing on developing a live-attenuated vaccine would consider the wild-type virus to be a single strain [8]. However, in the case of EV-A71, the study by Mandary et al. (2020) [12] indicated the presence of two variants in the wild-type population that were capable of forming big and small plaques. Plaque sizes could be indicative of in vitro infectivity [7,8,9,10,12] and the selection of a small plaque variant for use as attenuated seed strain would be desirable. This was proven to be the case for Dengvaxia, which utilized a small plaque-forming strain of DENV-2 [8].

In this study, four plaque variants (huge, big, medium, small) were isolated from EV-A71/WT grown in RD cells, while two plaque variants (medium, small) were isolated from EV-A71/WT passaged in Vero cells. All plaque variants were subjected to sequencing and the sequences were compared against the six haplotypes within the wild-type EV-A71. The RNA replication rate, growth kinetics, TCID_50_, viral binding, attachment and internalization studies of the EV-A71/SP small plaque variant isolated from RD cells indicated a much attenuated phenotype in comparison with the EV-A71/WT. A secondary structure of the 3D polymerase was predicted and analyzed to explore the effects of the changes in amino acid residues on in vitro virulence by in silico analysis. The 3D-S228P mutation could contribute to the decreased in vitro virulence observed in RD cells through in silico predictions.

## 2. Materials and Methods

### 2.1. Cell Lines

Human Rhabdomyosarcoma RD (ATCC^®^ CCL136^TM^, Manassas, VA, USA) and African green monkey kidney Vero (ATCC^®^ CCL-81^TM^) cells were grown in complete growth medium: Dulbecco’s modified minimal essential medium (DMEM) (Gibco, Carlsbad, CA, USA) supplemented with 10% fetal bovine serum (FBS) (Gibco, USA) and 1% penicillin/streptomycin (PSA). The cells were grown at 37 °C and supplemented with 5% CO_2_ until 70–90% confluency was achieved.

### 2.2. Passage of Virus

The wild-type EV-A71 subgenotype B4 strain 41 (GenBank: AF316321.2) was isolated from the lymph node of a deceased child who died from hand, foot and mouth disease during the Singapore 2000 outbreak. The genome sequence of this EV-A71 strain was deposited in GenBank with accession number AF316321.2. This clinical specimen was passaged in RD cells three times and the virus was named as EV-A71/WT [31] and was used for all in vitro growth analysis. The EV-A71/WT at MOI 1 was added to RD or Vero cell monolayers and incubated for 1 h at 37 °C in an incubator supplemented with 5% CO_2_. The viral supernatant was then discarded and the cells were washed with 1X PBS, followed by the addition of reduced growth media (DMEM, 2% FBS and 1% PSA). Upon observation of cytopathic effects of the cells, the virus was harvested by three cycles of freeze thawing and the viral supernatant was stored at −80 °C. This step was repeated for five times for viruses passaging in Vero cells to allow adaptation to the cell culture.

### 2.3. Plaque Assay

Approximately 1.5 × 10^5^ RD or Vero cells/mL were seeded into each well of a 6-well plate and maintained in complete growth medium for a day. Before viral infection, the medium was removed and washed with 1X PBS and then cells were infected with the viral inoculum from different 10-fold dilutions of the virus stock. After incubation for 1 h at 37 °C, the inocula were removed, the cells were washed with 1X PBS and 1mL plaque medium (2X DMEM containing 4% FBS and 2% PSA) and 1 mL of 1% agarose was added as overlay. After 72 h of incubation, cells were fixed with 3.7% formaldehyde and stained with 0.5% crystal violet. Plaque-forming units were counted.

### 2.4. Isolation of Plaque Variants

Approximately 1.5 × 10^5^ RD or Vero cells/mL (70–90% confluency) were seeded into each well of a 6-well plate and maintained in the complete growth medium. Before viral infection, the medium was removed and washed with 1X PBS and then infected with viral inoculum from different dilutions of the virus stock. After incubation for 1 h at 37 °C, the inocula were removed, cells were washed with 1X PBS and then 1mL of plaque medium and 1 mL of 1% agarose as the overlaying medium were added (Hydragene, Xiamen, China). The overlay was left to solidify before incubation at 37 °C for 3 days. After 3 days, agarose plugs containing plaques of interest were removed using a sterile glass pipette with a fine tip. Each plaque was added to separate wells of a 24-well tissue culture plate seeded with 1.5 × 10^5^ cells per well (70–90% confluency) in 500 µL of reduced growth media. The plates were incubated at 37 °C overnight in the presence of 5% CO_2_ before the plaque variants were harvested.

### 2.5. Re-Infection of Cells by Plaque Variants and the Determination of Plaque Sizes

Isolated plaques from RD and Vero cells were used to re-infect RD or Vero cells. After incubation for 1 h at 37 °C, the inocula were removed, the cells were washed with 1X PBS and then 1mL of plaque medium and 1 mL of 1% agarose were added as the overlaying media (Hydragene, Xiamen, China). The overlay was left to solidify before incubation at 37 °C for 3 days. After 3 days, the wells containing agarose overlay were fixed with 3.7% formaldehyde overnight and stained with 0.5% crystal violet (Sigma, St. Louis, MO, USA) to observe the plaques. Plaques were observed and images of the plaques were captured with the Immunospot^®^ S6 VERSA Analyzer (Cellular Technology Limited, Shaker Heights, OH, USA). Diameters of at least 40 plaque variants in monolayers of RD or Vero cells were measured using the DS-L3 viewer with the Nikon NIS-Elements software and analyzed by Graph pad prism 7.04 (San Diego, CA, USA). Section 2.4 had to be repeated using the harvested supernatant until homogenous plaque sizes were observed.

### 2.6. Growth Kinetics

RD cells at a density of 1.5 × 10^5^ cells/mL were seeded in the wells of several 6-well plates for 24 h. Cells were washed with 1X PBS and infected with EV-A71/WT and the small plaque variant (EV-A71/SP) in triplicates at a MOI of 0.1 and incubated for 1 h at 37 °C. Next, the virus inoculum was discarded and the RD cells were washed with 1X PBS before reduced growth media were added. The virus supernatants were harvested at 12 h, 24 h, 48 h and 72 h post-infection (h.p.i) after three cycles of freeze-thawing and were subsequently kept at −80 °C for determination of viral RNA copy numbers using RT-qPCR and plaque quantification using plaque assays.

### 2.7. Rates of Viral RNA Replication (RT-qPCR)

Primers targeting the VP1 sequence of the EV-A71 genome were obtained from a previous study [32]. The forward primer employed was 5′-GAGCTCTATAGGAGATAGTGTGAGTAGGG-3′, the reverse primer was 5′-ATGACTGCTCACCTGCGTGTT-3′ and the TaqMan probe used was 5′6-FAM-ACTTACCCA/ZEN/GGCCCTGCCAGCTCC-lowa Black FQ-3′. Viral RNA samples were extracted using the QIAamp Viral RNA mini kit (QIAGEN, Hilden, Germany) according to the manufacturer’s protocols. The reagents for RT-qPCR reaction tubes are described in Supplementary Material, Table S1. RT-qPCR was performed by Touch^TM^ Real-Time Reverse Transcriptase PCR Detection System, CFX96 (BIORAD, Hercules, CA, USA) using TaqMan^®^ Fast virus 1-step master mix (Life Technologies, Los Angeles, CA, USA) by reverse transcription for quantification of viral RNA copy number determination. The EV-A71/WT virus was used as the positive control. A non-template control consisting of master mix and dH_2_O was used as the negative control. The cycling conditions are as follows: 5 min at 50 °C, 40 amplification cycles at 95 °C for 3 s, 60 °C for 30 s. The serial dilutions of the standard viral samples were used to plot a standard graph and the RNA copy number was obtained using the default settings of the machine. Results were reported as positive if Cq was ≤40.36, based upon the signal threshold as calculated by the machine in default settings. Three independent experiments with two technical replicates were conducted for each sample.

### 2.8. Tissue Culture Infectious Dose (TCID_50_) Assays

The assay was conducted with reference to Yee et al. [20]. Approximately 2 × 10^5^ RD cells/mL were seeded into each well of a 96-well plate and maintained in complete growth medium for a day. Before viral infection, the medium was removed and washed with 1X PBS and then infected with the EV-A71/WT or EV-A71/SP from different dilutions (10^−1^ to 10^−8^) of the MOI 1 virus stock, with ten technical replicates each. Infections were carried out in quadruplicates and negative controls contained only uninfected RD cells. After incubation for 1 h at 37 °C, the inocula were removed and the cells were observed for cytopathic effects (CPE) for up to 48 h. The number of wells containing CPE across each dilution row were calculated for % cell death (e.g., 8 out of 10 wells that showed CPE indicated 80% cell death). The TCID_50_/_mL_ value was determined using the Reed and Muench formula [33,34], as described below:

$$\mathrm{PD}=\frac{\left(\%\;\mathrm{wells}\;\mathrm{infected}\;\mathrm{at}\;\mathrm{dilution}\;\mathrm{next}\;\mathrm{above}\;50\%\right)-\left(50\%\right)}{\left(\%\;\mathrm{wells}\;\mathrm{infected}\;\mathrm{at}\;\mathrm{dilution}\;\mathrm{next}\;\mathrm{above}\;50\%\right)-\left(\%\;\mathrm{wells}\;\mathrm{infected}\;\mathrm{at}\;\mathrm{dilution}\;\mathrm{next}\;\mathrm{below}\;50\%\right)}$$

logID_50_ = log (dilution with >50% positive) + PD × [−log (dilution factor)]

$${\mathrm{TCID}}_{50}{/}_{\mathrm{mL}}=\frac{\frac{1}{ID50}}{viral\;inoculum\;in\;mL}$$

### 2.9. Virus Binding Assay

The virus binding assay (cell-based ELISA) was performed according to Spurgers et al. (2013), with minor modifications [35]. RD cells (2 × 10^5^/mL) were grown overnight in the wells of a 96-well plate. Pre-chilled RD cells were infected with EV-A71/WT or EV-A71/SP at MOI 1 and further incubated at 4 °C for 1 h to allow for virus binding. The inoculum was removed after 1 h and unbound viruses were washed away with ice-cold PBS. RD cells were fixed with 80% acetone for 30 min, washed with PBS and blocked with 5% bovine serum albumin (BSA) (dissolved in PBS). Incubation with the anti-VP1 mouse primary monoclonal antibody (1:2000) (GeneTex, Irvine, CA, USA) in 1% BSA at room temperature was conducted for one hour. After incubation, the 96-well plate was washed with 1X Tris-buffered saline containing 0.1% Tween^®^ 20 detergent (TBST) and goat anti-mouse IgG HRP secondary antibody in 1% BSA (1:2000) (Santa Cruz Biotechnology, Dallas, TX, USA) was added and incubated for 1 h at room temperature. The excess antibody was removed by washing the plate with TBST. The substrate 3,3′,5,5′-Tetramethylbenzidine (TMB) (SeraCare Life Sciences Inc., Milford, MA, USA) was added and the plate was incubated in the dark for at least 20 min. A stop solution of 1M sulfuric acid (100 μL) was added and binding of the antibody with the viruses was measured at 450 nm using Infinite 200 Pro Multiplate Reader (Tecan, Mannededorf, Switzerland). The experiment was performed in triplicates. Positive antibody levels were defined as those above a cutoff OD value set at the mean OD of cell controls plus three standard deviations.

### 2.10. Viral Attachment Assay

The attachment assay was performed using the method previously described by Yeh et al. (2015) [36]. RD cells were grown overnight in wells within 6-well plates to 3 × 10^5^/2 mL. Pre-chilled RD cells were infected with EV-A71/WT or EV-A71/SP at MOI 1 and incubated at 4 °C for 1 h to allow virus attachment. The inoculum was removed after 1 h and RD cells were washed with PBS. Amounts of 1mL of plaque medium and 1 mL of 1% agarose were added as the overlaying media (Hydragene, Xiamen, China). After incubation for 3 days, the RD cells were fixed with 3.7% formaldehyde and stained with 0.5% crystal violet. The plaques were counted manually against a white background and images were taken using CTL Immunospot^®^ S6 Versa (Cleveland, OH, USA). All experiments were performed as three independent replicates. Percentage viral attachment of EV-A71/WT was assumed at 100%. Percentage viral attachment of EV-A71/SP was calculated as the number of plaques formed by EV-A71/SP over the number of plaques formed by EV-A71/WT.

### 2.11. Viral Entry Assay

The entry assay was performed using the method previously described by Yeh et al. (2015) [36]. EV-A71/WT or EV-A71/SP at MOI 1 at MOI 1 was added to the pre-chilled RD cells and incubated at 4 °C for 1 h to allow virus attachment. The inoculum was removed after 1 h and RD cells were washed with PBS to remove any unattached virus. The RD cells were then incubated at 37 °C for 1 h to allow virus entry. After 1 h, the medium was removed and RD cells were treated with PBS (pH 3) for 60 s at room temperature to inactivate the extracellular virus. After 60 s, the acidic pH was neutralized by the addition of PBS (pH 11) to each well. Cells were then washed with serum-free media. Amounts of 1 mL of plaque medium and 1 mL of 1% agarose were added as the overlaying media (Hydragene, Xiamen, China). After incubation for 3 days, the RD cells were fixed with 3.7% formaldehyde and stained with 0.5% crystal violet. The plaques were counted manually against a white background and images were taken using CTL Immunospot^®^ S6 Versa. All experiments were performed as three independent replicates. Percentage viral entry of EV-A71/WT was assumed at 100%. Percentage viral entry of EV-A71/SP was calculated as the number of plaques formed by EV-A71/SP over the number of plaques formed by EV-A71/WT.

### 2.12. cDNA Preparation and Amplification

Viral RNA was isolated from each individual plaque variant using QIAmp^®^ Viral RNA Mini Spin Kit (Qiagen, Redwood City, CA, USA) and used according to manufacturer’s protocols. The purified RNA was reverse-transcribed into cDNA using the SuperScript^®^ IV First Strand Synthesis System (ThermoFisher Scientific, Waltham, MA, USA) and oligo (dT)_20_ primer. Each cDNA synthesis mixture contained master mix and the viral RNA as a template. PCR amplification of a ~7.5 kb cDNA was performed by Q5^®^ High-Fidelity DNA polymerase (NEB, Ipswich, MA, USA) using 5′-UTR forward and 3D-3′UTR reverse primers (sequence in Supplementary Material, Table S2). The following cycling conditions were employed for the PCR reactions: 98 °C for 30 s, followed by 98 °C for 10 s, 62 °C for 30 s and 72 °C for 3 min 45 s for 35 cycles. The cycles were terminated with a 2 min extension at 72 °C. Agarose gel (1%) was prepared in 1 × Tris-acetate-EDTA (TAE) buffer. SYBR^TM^ Safe DNA Gel Stain (Invitrogen, CA, USA) was added to the gel to reach a final concentration of 0.5 μg/mL. Gel Loading Dye Purple (6×) (NEB, MA, USA) stain was mixed with DNA samples before loading into the wells. The GeneRuler 1 kb DNA Ladder (ThermoFisher Scientific, MA, USA) was used for determination of DNA size fragments in the gel. After electrophoresis at 100 V for 1 h, the gel was analyzed.

### 2.13. Genome Sequencing

PCR products obtained from Materials and Methods Section 2.12 were characterized by size through agarose gel electrophoresis and subjected to Sanger sequencing (Apical Scientific, Selangor, Malaysia) using designed primers stated in Supplementary Material, Table S2. Sequences were assembled for construction of the complete EV-A71 genome using Geneious Software (Biomatters, Auckland, New Zealand) using default settings at high/medium sensitivity. Clustal Omega (Conway Institute UCD, Dublin, Ireland) was used for sequence alignment using default settings.

### 2.14. Homology Modeling and Structure Analysis

Template search was performed by aligning the sequence of EV-A71/WT 3D polymerase with other 3D polymerase of enterovirus using PSI-BLAST from NCBI based on the PDB database. The top homologues were ranked based on the alignment score and structure quality. Based on the PSI-BLAST result, the crystal structure of the 3D polymerase of four enterovirus 71 strains and one coxsackie A16 strain with the PDB ID of 6KWQ, 5Y6Z, 4IKA, 6LSE and 3N6L with more than 94% identity were retrieved from PDB database and used as templates for structure predictions of the 3D polymerase of EV-A71/WT and EV-A71/SP. The 3D polymerase structures were modeled using the YASARA structure software version 21.6.17 (http://www.yasara.com, accessed on 8 August 2022). An unrestrained high-resolution refinement with explicit solvent molecules was run, using YASARA 03 force fields. The result was validated by the YASARA program, using the default parameter setting to ensure that the refinement did not move the model in the wrong direction, based on the template used to predict the structure. For each template, five models were built. To refine the geometry and orientation of the binding model, the highest-ranked models for each template were subsequently refined using the YASARA program. In the refinement, after the side chains had been built, optimized and fine-tuned, all the newly modeled parts were subjected to a combined steepest descent and simulated annealing minimization (i.e., the backbone atoms of the aligned residues were kept fixed to avoid potential damage) until the force field energy converged. Then, YASARA combined the best parts of the models to obtain a hybrid model, to increase the accuracy beyond that of each of the contributors. Finally, the model was subjected to a final round of simulated annealing minimization in an explicit solvent and the quality Z-scores were obtained fully automatically. Superimpositions of 3D of EV-A71/WT against EV-A71/SP were conducted using the default parameter for Cα of the residue in both structures. and the differences in RMSD were analyzed by the YASARA software for those amino acids where their Cα were not matched.

Information of all salt bridges within the structures was obtained through ESBRI at a cutoff distance of 4.0 Å (evaluating the salt bridges in proteins) (http://bioinformatica.isa.cnr.it/ESBRI/, accessed on 8 August 2022).

### 2.15. Next-Generation Sequencing

Viral RNA was extracted from EV-A71/WT using the QIAmp^®^ Viral RNA Mini Spin Kit (Qiagen, CA, USA). The kit was used according to the manufacturer’s instructions. The purified RNA was reverse-transcribed into cDNA by using the SuperScript^®^ IV First Strand Synthesis SuperMix Kit (Thermofisher, USA) and oligo (dT)_20_ primer. Full length cDNA of EV-A71 was used as the template and was amplified with Q5^®^ High-Fidelity DNA Polymerase (NEB, Ipswich, MA, USA). The forward and reverse primer used are the 5′UTR forward and 3D-3′UTR reverse primers stated in Supplementary Material, Table S2. The following cycling conditions were employed for the PCR reactions: 98 °C for 30 s, followed by 98 °C for 10 s, 62 °C for 30 s and 72 °C for 3 min 45 s for 35 cycles. The cycles were terminated with a 2 min extension at 72 °C. The products were purified by a QIAQuick^®^ PCR purification kit (Qiagen, CA, USA) and size-selected by agarose gel electrophoresis (7.4 kb). The PCR product was applied to paired-end Illumina sequencing (Apical Scientific, Selangor, Malaysia). Input genomic DNA (1–5 μg) was fragmented by hydrodynamic shearing to generate ~350 bp fragments using a Bioruptor^®^ Pico sonication device (Diagenode, Liege, Belgium) for 15 cycles “ON/30”OFF. The fragments were blunt ended and phosphorylated and a single ‘A’ nucleotide was added to the 3′ ends of the fragments in preparation for ligation to an adapter that had a single-base ‘T’ overhang. An adapter ligation at both ends of the genomic DNA fragment conferred different sequences at the 5′ and 3′ ends of each strand in the genomic fragment. The sequence of the 5′ and 3′ adapters were 5′-AATGATACGGCGACCACCGAGATCTACACTCTTTCCCTACACGACGCTCTTCCGATCT-3′ and 5′-GATCGGAAGAGCACACGTCTGAACTCCAGTCACATCACGATCTCGTATGCCGTCTTCTGCTTG-3′, respectively. The resulting sample library was purified using an AMPure XP system (Beckman Coulter, Indianapolis, IN, USA) and checked for size distribution using an Agilent 2100 Bioanalyzer (Agilent Technologies, Santa Clara, CA, USA). The final purified product was then quantitated prior to seeding clusters on a flow cell. The library flow cell lane was named as FDMS202341864-1a_H7MVCCCX2_L7 and showed a Q-score of Q20, indicating 99% of right bases. Variant analysis was then performed with the following methods (BIOEasy, Shah Alam, Malaysia). Reads were mapped by BBMap of the BBTools packages (https://jgi.doe.gov/data-and-tools/bbtools/, accessed on 22 January 2021). Paired-end Illumina sequences were first removed of sequence adaptors and reads with low-quality scores using BBDuk of the BBTools packages (https://jgi.doe.gov/data-and-tools/bbtools/, accessed on 22 January 2021). Low-quality reads were defined as reads containing N > 10% (where N represents the base that cannot be determined) and those with Qscore ≤ 5 (low-quality base that is over 50% of the total base.) Reads were aligned using SortSam of the Picard Tools package (https://broadinstitute.github.io/picard/, accessed on 22 January 2021). Variant calling was performed using FreeBayes (https://github.com/ekg/freebayes, accessed on 22 January 2021) against enterovirus 5865/sin/00009 as the reference genome [37]. Mapped reads were further subjected to CliqueSNV analysis (https://github.com/vtsyvina/CliqueSNA/blob/master/README.md, accessed on 22 January 2021), a novel reference-based method for reconstruction of viral variants from NGS data [38]. Parameters of BBMap, BBDuk, SortSam, FreeBayes and Clique SNV are listed in Supplementary Material, Data S3. Nucleotide sequence comparisons of the six haplotypes and enterovirus strain 41 subgenotype B4 and nucleotide sequence comparisons of the six haplotype and plaque variants were performed by Clustal Omega 2.1 Identity Matrix (Conway Institute UCD, Dublin, Ireland).

### 2.16. Data Analyses

Data were analyzed using GraphPad Prism, version 7 (GraphPad Software, San Diego, CA, USA) to determine the plaque size, growth kinetics, RNA replication rate, TCID_50_, viral binding studies, viral attachment and entry assays. Data presented are the average ± S.D. of three or four independent experiments and analyzed by Student’s *t*-test or ANOVA (** *p* < 0.01, *** *p* < 0.001, **** *p* < 0.0001). ANOVA used α value of 0.01. Tukey’s test was used for correction of multiple corrections.

## 3. Results

### 3.1. Next-Generation Sequencing (NGS) of EV-A71/WT

EV-A71 is an RNA virus that is capable of introducing 10^−4^ to 10^−5^ mutations per replication cycle due to the low fidelity of the RNA polymerase. The high mutation rates could allow the formation of a quasispecies population within the genome that comprises different progenies with different genomic makeups, termed haplotypes. Next-generation sequencing (NGS) could provide a more in-depth analysis of haplotypes that are present within a viral population. After the reconstruction of viral variants using CliqueSNV, the EV-A71/WT was found to contain six haplotypes within the viral population. Raw genome sequences of the six haplotypes can be referred to in Supplementary Material, Data S4. The sequences of the six haplotypes were compared against the sequences of the EV-A71 subgenotype B4 strain 41 (GenBank: AF316321.2) and percentage similarities are presented in Table 1. EV-A71/Hap2 and EV-A71/Hap4 showed the highest percentage similarities relative to the EV-A71 subgenotype B4 strain 41 at 99.80%. EV-A71/Hap1 and EV-A71/Hap3 were placed second, with 99.78% similar nucleotide sequences as the EV-A71 subgenotype B4 strain 41, while EV-A71/Hap0 and EV-A71/Hap5 had 99.76% percentage similarities relative to the EV-A71 subgenotype B4 strain 41.

Relative frequencies of the six haplotypes are presented in Table 2. The EV-A71/Hap0 was found to be the most abundant haplotype within the EV-A71/WT population, at a percentage frequency of 36.78%. EV-A71/Hap1 was the second abundant haplotype at 17.07%, while EV-A71/Hap2, EV-A71/Hap3 and EV-A71/Hap4 had percentage frequencies at 15.83%, 9.89% and 9.64%, respectively. EV-A71/Hap5 was the least abundant haplotype and only accounted for 6.86% of the whole EV-A71/WT population. The remaining 3.93% accounted for unclassified single nucleotide polymorphism (SNV) pairs. The presence of unaccounted SNV pairs could occur for various reasons, as assembly of haplotype populations from NGS was usually complicated by sequencing errors, an unknown number of haplotypes in a sample and the genetic similarity of haplotypes within a sample [38]. Sequences of the six haplotypes were compared and a summary of the nucleotide differences were found at 15 positions (Table 2).

Haplotypes 1 and 3 were the only haplotypes that showed a uridine at nt. 309 and a cytosine at nt. 290, respectively. Interestingly, the VP1-97 in haplotype 2 was leucine (L), but threonine (T) was present in haplotypes 0, 1, 3, 5, whilst haplotype 4 contained isoleucine (I). Haplotype 2 was the only haplotype to harbor serine at aa. 104, asparagine at aa.237, proline at aa. 246 and aspartate at aa. 282, when compared against the five other haplotypes. Haplotype 0 is the only haplotype among the six that had isoleucine at VP2-141, whilst haplotype 4 had a unique tyrosine residue at aa.71 of the 2A protein. Except for haplotypes 2 and 4, the 292A in the VP1 region was also found in the remaining four haplotypes.

### 3.2. Plaque Morphology of Wildtype EV-A71 and the Plaque Variants

**Figure 1:**
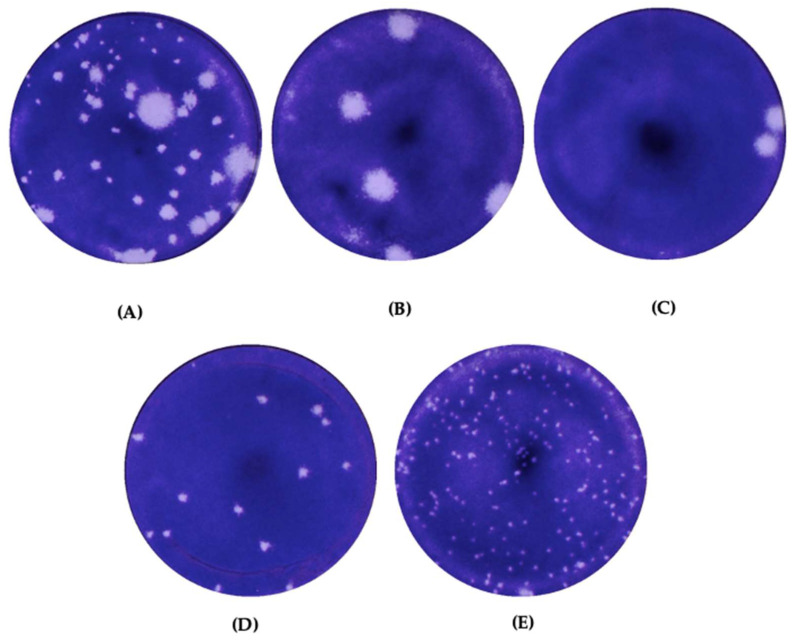
Plaque morphology of (**A**) EV-A71/WT cultured in Rhabdomyosarcoma (RD) cells, (**B**) EV-A71/HP plaque variant, (**C**) EV-A71/BP plaque variant, (**D**) EV-A71/MP plaque variant and (**E**) EV-A71/SP plaque variant. The plaques were visualized 72 h post infection (h.p.i.) after staining with crystal violet. Plaques were observed and images were captured with an Immunospot^®^ S6 VERSA Analyzer (Cellular Technology Limited, USA).

**Figure 2:**
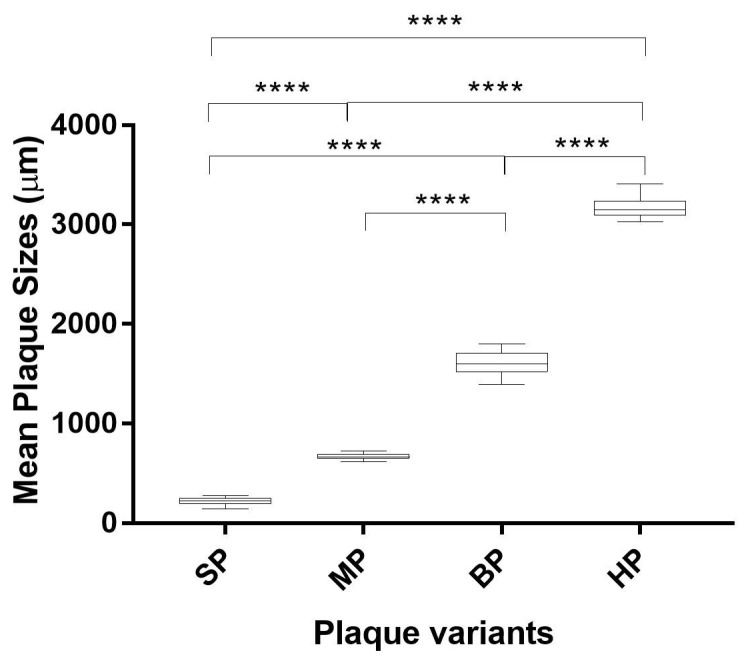
Diameters of 40 plaques representing each plaque variant in monolayers of RD were measured using the DS-L3 viewer with the Nikon’s NIS-Elements software and analyzed by Graph pad prism 7.04. The mean size of all plaque variants were compared against all plaque variants in RD cells. Error bars indicate standard deviation of average. Statistical analysis, one-way ANOVA, **** *p* < 0.0001.

The EV-A71/WT was propagated in RD cells and plaques of various sizes were observed (Figure 1A). Diameters of plaque variants were measured and were categorized into four groups that differed significantly in size. The plaques formed in RD cells were isolated and passaged in RD cells to obtain purified plaque variants. Four different types of plaque variants were isolated after purifications and designated as EV-A71/HP (huge plaque variant) (Figure 1B), EV-A71/BP (big plaque variant) (Figure 1C), EV-A71/MP (medium plaque variant) (Figure 1D) and EV-A71/SP (small plaque variant) (Figure 1E) based on different sizes of the plaques. Diameters of the plaque variants isolated from RD cells were measured using an average of 40 plaques for each variant (Figure 2). All four plaque variants were distinctly different in size when compared with each other. The EV-A71/HP variant was nearly 3000 µm in diameter (3161.84 ± 102 µm), followed by the EV-A71/BP variant (1608.36 ± 119 µm), EV-A71/MP variant (669.86 ± 31 µm) and the EV-A71/SP variant (222.56 ± 37 µm). Hence, the results indicated that there were significant differences in size as indicated by their diameters among the four plaque variants (one-way ANOVA, F (degree of freedom) = 11, *p* < 0.0001) (Figure 2).

### 3.3. Comparison of Genome Sequences among Plaque Variants in RD Cells

**Figure 3:**
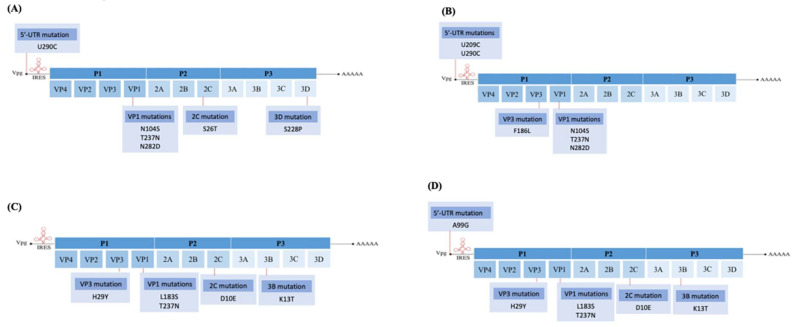
Differences in amino acid sequences between the (**A**) EV-A71/SP small plaque variant, (**B**) EV-A71/MP medium plaque variant, (**C**) EV-A71/BP big plaque variant and (**D**) EV-A71/HP huge plaque variant isolated from RD cells in comparison with the EV-A71/WT. VP indicates viral protein.

EV-A71 is an RNA virus known for its high mutation rates due to its low fidelity RNA polymerase. There could be genome differences among the four purified plaque variants. Purified plaque variants derived from the four plaque variants were subjected to DNA sequencing and the sequence of each variant was compared against the sequence of the EV-A71/WT (Figure 3). Raw genome sequences of the four plaque variants can be referred to in Supplementary Material, Data S4. The mutations present in each of the four plaque variants compared with the EV-A71/WT are presented in Supplementary Material, Table S5. All four plaque variants carried a common non-synonymous mutation at T237N in the VP1 region. The EV-A71/SP variant carried exclusive mutations at S26T in the 2C protein and S228P in the 3D polymerase. The EV-A71/MP variant, on the other hand, displayed a nucleotide substitution at 5′-UTR U209C and a single amino acid substitution at F186L in the VP3, while other variants lacked these substitutions. Interestingly, both EV-A71/BP and EV-A71/HP variants exhibited the same mutations that were lacking in the EV-A71/SP and EV-A71/MP variants. These mutations were in the VP3 (H29Y), VP1 (L183S), 2C (D10E) and 3B (K13T) regions, except for an exclusive nucleotide mutation, A99G, found only in the 5′-UTR region of the EV-A71/HP variant.

The sequences of all four plaque variants were compared against the six haplotypes to distinguish the genetic relationships between haplotypes present in the EV-A71/WT population and the four purified plaque variants. Nucleotide similarity between the plaque variants and the haplotypes are presented in Table 3. Interestingly, amongst all four plaque variants, EV-A71/SP, EV-A71/MP, EV-A71/BP and EV-A71/HP showed the highest similarity with EV-A71/Hap2, with similarity percentages at 99.92%, 99.95%, 99.82% and 99.81%, respectively. When amino acid sequences of the plaque variants were compared against the sequences of haplotypes revealed through NGS, the EV-A71/SP and EV-A71/MP variants exhibited all amino acid residues found in the VP1 of EV-A71/Hap2, which were Leu97, Ser104, Asn237, Pro246, Asp282 and Thr292. On the other hand, both EV-A71/BP and EV-A71/HP variants had the same amino acid residues in VP1 as the EV-A71/Hap2, except Ser104 and Asp282. This indicated that Ser104 and Asn282 were not involved in the adaptation of EV-A71/BP and EV-A71/HP variants in RD cells.

### 3.4. Adaptation of EV-A71 in Vero Cells and Isolation of Plaque Variants

**Figure 4:**
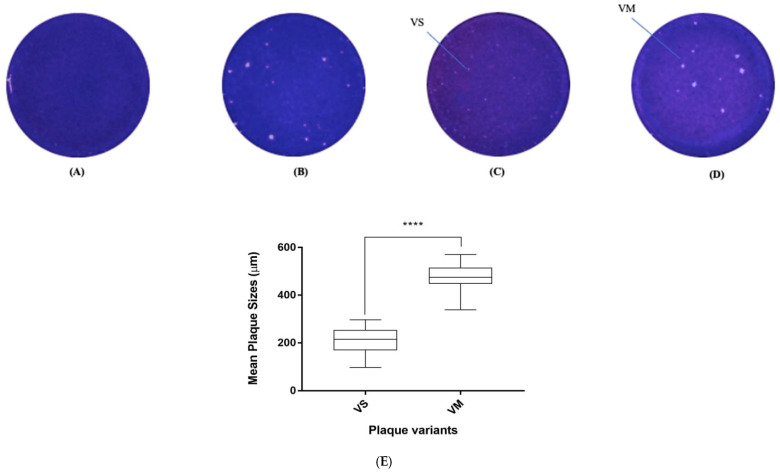
Six-well plates containing Vero cells with 70–80% confluency were infected with the EV-A71/WT after (**A**) 1st passage (P1) and (**B**) 5th passage (P5). (**C**) Plaque morphology of EV-A71/VS plaque variant and (**D**) EV-A71/VM plaque variant after 5th passage. The plaques were visualized 72 h post-infection (h.p.i.) after staining with crystal violet. Plaques were observed and images were captured with Immunospot^®^ S6 VERSA Analyzer (Cellular Technology Limited, USA). (**E**) Diameters of 40 plaques representing each plaque variant in monolayers of Vero cells were measured using the DS-L3 viewer with the Nikon’s NIS-Elements software and analyzed by Graph pad prism 7.04. The mean size of the EV-A71/VS variant was compared against the mean plaque size of the EV-A71/VM variant in Vero cells. Error bars indicate standard deviation of average. Statistical analysis, unpaired *t*-test, **** *p* <0.0001.

The EV-A71/WT was propagated in Vero cells to observe the type of plaque variants formed when it was grown in a different cell line. The virus was propagated five times in Vero cells and the viral supernatants from the first (P1) and fifth (P5) passages were plated in Vero cells (Figure 4A,B). Interestingly, as Vero cells were not used as the main host cells for isolation of EV-A71 from the clinical samples, no plaques were formed in this host cell during the first passage. After a few passages, EV-A71 started to adapt to the new cell culture environment and began to yield plaques of two sizes, the small plaque (EV-A71/VS) and the medium plaque (EV-A71/VM) from the fifth passage. The plaques formed in Vero cells were isolated and passaged to obtain purified plaque variants (Figure 4C,D).

Diameters of the plaque variants isolated from Vero cells (Figure 4E) were measured using an average of 40 plaques for each variant. The two plaque variants were distinctly different in sizes when compared with each other (unpaired *t*-test, F = 78, *p* < 0.0001). The EV-A71/VS variant had quite similar diameters (212.86 ± 50 µm) as the EV-A71/SP variant (222.56 ± 37 µm) that was isolated from viruses passaged in RD cells, but there was a 10 µm difference. On the other hand, the medium plaque isolated from Vero cells (EV-A71/VM) had a different plaque size of 476.09 ± 53 µm when compared with the medium plaque (EV-A71/MP) isolated from RD cells, which had a plaque diameter of 669.86 ± 31 µm.

**Figure 5:**
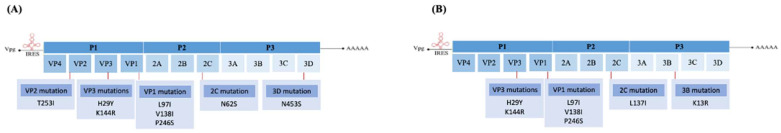
Differences in amino acid sequences between (**A**) EV-A71/VS variant and (**B**) EV-A71/VM variant isolated from Vero cells in comparison with the EV-A71/WT.

Raw genome sequences of the two plaque variants can be accessed in Supplementary Material, Data S4. The mutations present in each of the two plaque variants compared with the EV-A71/WT are presented in Supplementary Material, Table S5. In the case of plaque variants isolated from Vero cells, the EV-A71/VS variant displayed distinct mutations at T253I of the VP2 region, N62S in the 2C and N453S in the 3D polymerase (Figure 5). On the other hand, the EV-A71/VM exhibited mutations at L137I in the 2C and K13R in the 3B protein.

Nucleotide sequences of the two plaque variants were compared with the six haplotypes to distinguish the genetic relationships between haplotypes present in the wild-type viral population and the two purified plaque variants. Nucleotide similarity between the two plaque variants and the six haplotypes are presented in Table 4. Interestingly, the two plaque variants, EV-A71/VS and EV-A71/VM, showed the highest similarity with EV-A71/Hap4, with similarity percentages at 99.82% and 99.76%, respectively. Both EV-A71/VS and EV-A71/VM showed the same amino acids in the VP1 region, namely Ile97, Asn104, Thr237, Ser246, Asn282 and Thr292, as those found with the EV-A71/Hap4 haplotype. One exception was the amino acid residue Tyr71 present in the 2A protein of the EV-A71/Hap4, which was not found in either of these two plaque variants. It is possible that the six mutations found within the VP1 of the EV-A71/Hap4 allowed adaptations to Vero cells.

### 3.5. In Vitro Virulence Characteristics of the Small Plaque Variants Isolated from RD Cells

#### 3.5.1. Growth Kinetics and RNA Replication Rate

**Figure 6:**
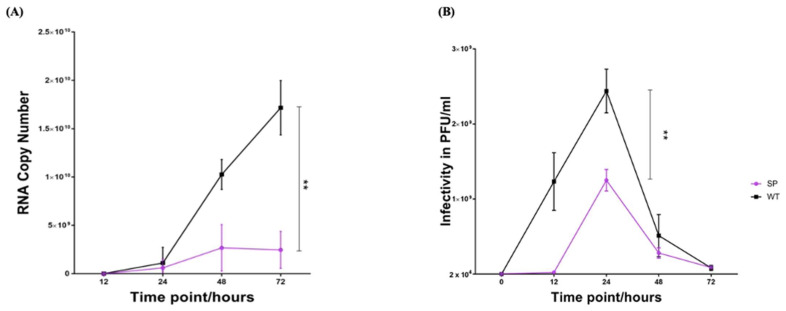
(**A**) Comparison of RNA replication rates and (**B**) plaque forming ability (PFU/mL) of EV-A71/WT (black) and EV-A71/SP (magenta) plated in RD cells. Viral supernatants were harvested from infected RD cells at 12, 24, 48 and 72 h.p.i. and subjected to plaque and qRT-PCR assays in triplicates to yield PFU/mL and RNA copy number, respectively. Statistical analysis, unpaired *t*-test, ** *p* < 0.01.

Analysis of growth kinetics of the EV-A71/SP variant in RD cells showed that the variant had a lower RNA replication rate when compared with the EV-A71/WT (unpaired *t*-test, F = 4, *p* < 0.01) (Figure 6A). At 12 h.p.i., the EV-A71/WT was observed to replicate faster (yielding 1.11 × 10^9^ viral copy number) than the EV-A71/SP (yielding 6.05 × 10^8^ viral copy number). At 24 h.p.i., EV-A71/WT had RNA copy numbers at 1.03 × 10^10^, which was 1 log higher in comparison to the replication rate of the EV-A71/SP variant (yielding 2.67 × 10^9^ viral copy number). After this, the EV-A71/WT was still replicating efficiently and showed exponential growth right up to 72 h.p.i (yielding 1.72 × 10^10^ viral copy number), while the replication rate of the EV-A71/SP variant remained static and reached a plateau at 48 h.p.i. (yielding 2.67 × 10^9^ viral copy number). No further increase of copy number was observed from 48 to 72 h.p.i. and the viral copy number remained at 2.46 × 10^9^.

Quantification of plaque-forming units (PFU) is another way to determine the infectivity of the plaque variants. Both the EV-A71/WT and the EV-A71/SP variant started off with 2 × 10^4^ PFU/mL of viruses which were used for infection of RD cells. The PFU of the EV-A71/WT started to rise steeply at 12 h.p.i. with 1.23 × 10^9^ PFU/mL, while the plaque-forming ability of the EV-A71/SP variant was slower (2.05 × 10^7^ PFU/mL). Both the EV-A71/WT and the EV-A71/SP variant exhibited maximum infectivity at 24 h.p.i., with 2.44 x 10^9^ PFU/mL and 1.25 × 10^9^ PFU/mL, respectively (unpaired *t*-test, F = 4, *p* < 0.01). Both plaque-forming abilities of the EV-A71/WT (5.13 × 10^8^ PFU/mL) and EV-A71/SP variant (2.80 × 10^8^ PFU/mL) dropped drastically at 48 h.p.i. and reached approximately 8.0 × 10^7^ PFU/mL at 72 h.p.i. (Figure 6B), possibly due to the lack of live host cells needed for the growth and replication of viruses to occur.

#### 3.5.2. TCID_50_

**Figure 7:**
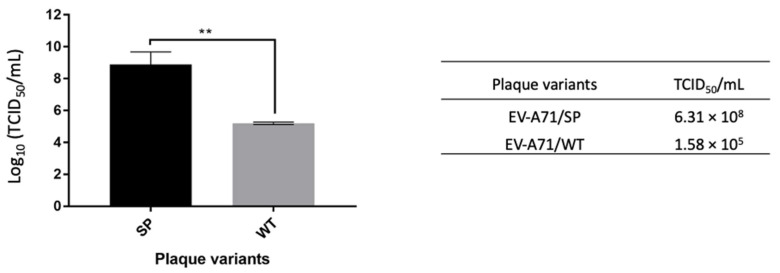
Tissue culture infectious dose (TCID_50_) of the EV-A71/SP variant compared with the EV-A71/WT. The values were derived from three independent experiments and the average value was presented. Statistical analysis, unpaired *t*-test, ** *p* < 0.01.

The EV-A71/SP variant displayed a higher log_10_ TCID_50_/mL value (log_10_ 8.80 ± 0.8), which was approximately 3-logs higher than the EV-A71/WT (log_10_ TCID_50_/mL = log_10_ 5.2 ± 0.1) (unpaired *t*-test, F = 4, *p* < 0.01) (Figure 7). Higher TCID_50_/mL indicated higher numbers of small plaque variants that were needed to cause 50% total cell death when compared with the wild type.

#### 3.5.3. Viral Binding Studies

**Figure 8:**
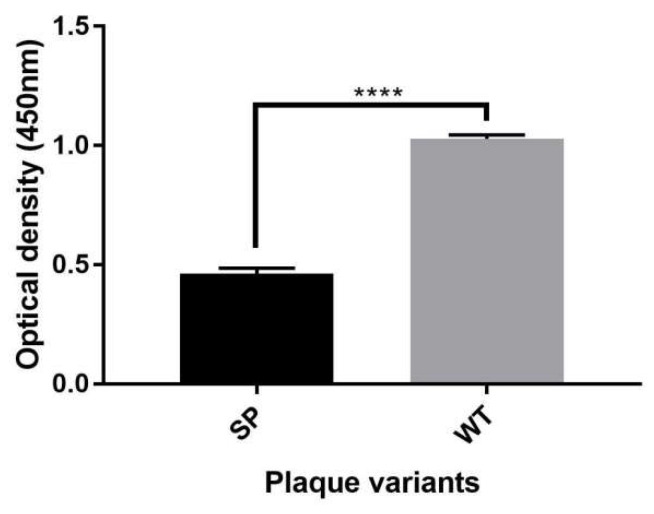
Viral-binding ability of EV-A71/SP was analyzed using ELISA performed in RD cells in comparison with the EV-A71/WT. The experiments were repeated three times and the average value is presented. Error bars indicate standard deviation of average. Statistical analysis, unpaired *t*-test, **** *p* < 0.0001.

The EV-A71/SP variant showed significantly reduced viral-binding ability when compared with the EV-A71/WT and this explained the reduced plaque forming units of the EV-A71/SP variant in RD cells (unpaired *t*-test, F = 4, *p* < 0.0001). This indicated lesser viral loads and lower in vitro virulence in RD cells (Figure 8). The EV-A71/WT showed higher viral-binding ability at an OD_450_ of 1.03 ± 0.02. The binding capability of the EV-A71/SP variant at an OD_450_ of 0.46 ± 0.02 showed significant reduction by 55% when compared with the EV-A71/WT.

#### 3.5.4. Viral Attachment and Entry

**Figure 9:**
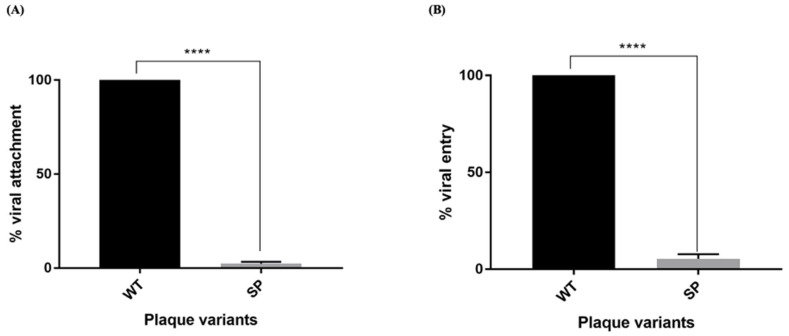
(**A**) Viral attachment and (**B**) viral entry of the EV-A71/SP variant, corresponding with the EV-A71/WT. The experiment was conducted as three independent experiments and the average value is presented. Error bars indicate standard deviation of average. Statistical analysis, unpaired *t*-test, **** *p* < 0.0001.

To assess viral attachment of EV-A71/SP, the viruses were added to RD cells and incubated for 1 h at 4 °C to allow attachment of the virus to the cells. Later, the inoculum was removed and the cells were incubated at 37 °C to facilitate the entry of attached viruses to infect the cells. Average viral attachments of the EV-A71/SP variant and EV-A71/WT are presented (Figure 9A). The results showed that the EV-A71/SP variant exhibited 2.27% of viral-attachment ability when compared with EV-A71/WT, which expressed a value of 100% (unpaired *t*-test, F = 4, *p* < 0.0001).

To assess viral internalization of EV-A71/SP, viruses were pre-attached to RD cells at 4ºC for one hour and the incubation temperature was immediately shifted to 37 °C for 1 h to facilitate entry of the pre-attached virus into the cells. Viruses that did not enter the cells were inactivated using acidic PBS (pH 3) followed by neutralization using alkaline PBS (pH 11) and the plaques were counted after 72 h. The average viral entry of the EV-A71/SP variant and the EV-A71/WT is presented in Figure 9B. The EV-A71/SP variant demonstrated a viral entry percentage of 5.3% when compared with the EV-A71/WT (unpaired *t*-test, F = 4, *p* < 0.0001).

### 3.6. Homology Modeling and the Analysis of Mutation in the Small Plaque Variant Isolated from RD Cells

#### 3D Polymerase

Since the EV-A71/SP variant carried several spontaneous mutations (2C-S26T, 3D-S228P), 3D structure modeling would indicate mutations that could affect viral functionality. In this case, the S228P mutation was predicted to affect catalysis, leading to reduced viral replication. This could guide future efforts of conducting site-directed mutagenesis on this specific amino acid residue.

EV-A71 undergoes a viral replication process using the RNA-dependent RNA polymerase enzyme (3D^pol^). In general, the 3D^pol^ is made up of the thumb, fingers and palm domains, which in turn are composed of conserved motifs A-G [39]. In this study, the EV-A71/SP variant showed an amino acid substitution at position S228P in the 3D^pol^. Both 3D protein structures of the EV-A71/WT (green) and the EV-A71/SP variant (magenta) were superimposed and resulted in a RMSD value of 0.6457Å (Table 5). The RMSD value indicated that there were slight differences between the secondary structures of both of the 3D polymerases (Table 5). The EV-A71/SP variant had 414 hydrogen bond interactions within its 3D^pol^ structure and showed 24 more hydrogen bonds than those present in the EV-A71/WT (390 H_2_ interactions). The secondary structures between the two also showed slight differences. The EV-A71/SP variant showed a decrease in α-helix structures when compared with the EV-A71/WT, but an increase in both turns and coil structures were observed. On the other hand, the EV-A71/WT had 0.6% more ß-sheet structures in comparison with the EV-A71/SP variant.

**Figure 10:**
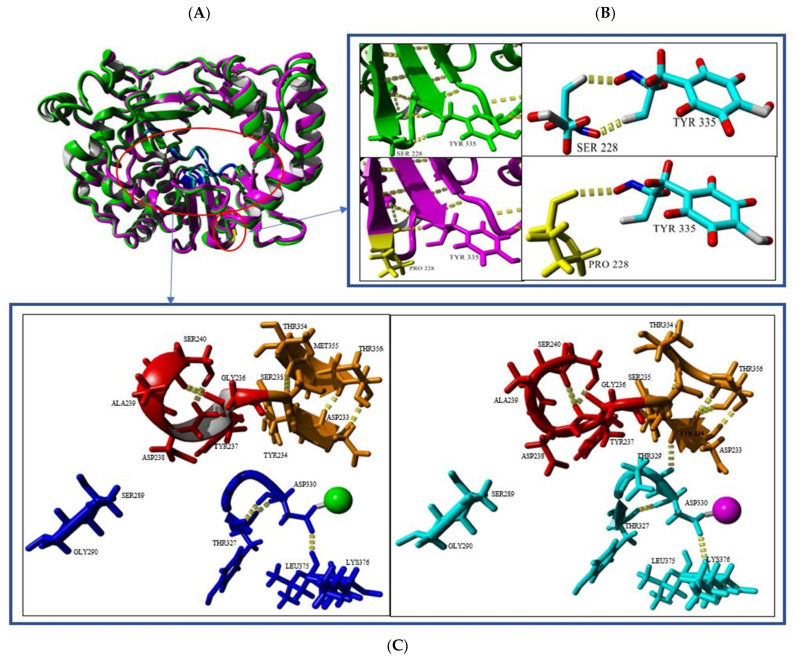
(**A**) The superimposition of the 3D polymerase protein structure of EV-A71/WT and the EV-A71/SP variant. EV-A71/WT is colored in green, while EV-A71/SP is colored in magenta. The active sites of EV-A71/WT consisting of amino acids 233–238, 289–290, 327, 328–330, 375–376 are marked in blue, while the active site of EV-A71/SP is marked in cyan. Mutation S228P is denoted in yellow. (**B**) A close-up view of the S228P mutation found in the 3D polymerase of EV-A71/SP (bottom) when compared with the EV-A71/WT (top). Mutated Pro228 is marked in yellow. Hydrogen bonds are denoted as yellow dotted lines. (**C**) A close-up view of the differences between the active site in the 3D polymerase of EV-A71/SP variant (right) when compared with EV-A71/WT (left). Turn structure within the active site of the EV-A71/WT and equivalent amino acids in the EV-A71/SP are marked in red. Interaction of Ser240 towards Gly236 and Asp237 are responsible for the formation of turn structure in the active site of EV-A71/WT. A longer sheet structure was found in Asp233-Ser235 of the EV-A71/WT active site, which were formed through Ser235-Thr354 and two hydrogen bonds between Asp233-Thr356, whereas three hydrogen bond interactions between Asp233-Thr356, Tyr234-Thr356 of the EV-A71/SP active site caused a shorter sheet to be formed. EV-A71/SP active site had an extra hydrogen bond between Tyr234-Asp329. Asp330 of the EV-A71/WT active site formed two hydrogen bonds with Tyr327 and one hydrogen bond with Leu375, whilst Asp330 of EV-A71/SP active site formed hydrogen bond interactions with Tyr327 and Lys376, respectively.

The EV-A71/SP variant was observed to carry a single S228P mutation in the 3D polymerase and this could affect the 3D structure. Both serine and proline were polar amino acids with uncharged R groups. In the EV-A71/WT, serine at position 228 of the 3D^pol^ formed two hydrogen bonds with tyrosine at position 335. However, a substitution to proline at position 228 only formed a single hydrogen bond with tyrosine at position 335 (Figure 10B). Interestingly, although this single amino acid substitution was located at the bottom of the active site, it indirectly caused the 3D^pol^ of the EV-A71/SP variant to lose a portion of the ß-sheet (amino acids 233–235) and helix (amino acids 236–238) structure in the active site, which might explain the slower growth of the EV-A71/SP variant caused by disruption of the active site of the 3D polymerase (Figure 10C). The active site of the 3D^pol^ was composed of 13 amino acids, consisting of amino acids 233–238, 289–290, 327, 328–330, 375–376 (PDB ID: 3N6L_A, 5Y6Z_A, 4IKA_A). Ser240 was responsible for the formation of the helix structure within the active site of EV-A71/WT as it could interact with Gly236 and Asp237. The short ß-sheet structure in the active site of EV-A71/SP variant was formed through three hydrogen bondings between Asp233-Thr356, Tyr234-Thr356. The longer sheet structure in EV-A71/WT was formed by two hydrogen bonds between Asp233-Thr356 and another hydrogen interaction at Ser235-Thr354. Furthermore, three other hydrogen bonds were found in the active sites of both EV-A71/WT and the EV-A71/SP variant. The EV-A71/SP active site had an extra hydrogen bond between Tyr234-Asp329. The EV-A71/WT had two Tyr327-Asp330 hydrogen bonds, while EV-A71/SP only contained one. Asp330 of EV-A71/WT was found to bind to Leu375, while the Asp330 of EV-A71/SP could only form a hydrogen bond with Lys376 (Figure 10C).

A list of salt bridge interactions within the 3D^pol^ structures of both the EV-A71/WT and the EV-A71/SP variant is presented in Supplementary Material, Table S6. Salt bridges could play important roles in protein structure and function and have stabilizing and destabilizing effects in protein folding. The ESBRI (http://bioinformatica.isa.cnr.it/ESBRI/, accessed on 8 August 2022) software used was available as a web tool that analyzed the salt bridges in a protein structure, starting from the atomic coordinates. The software could predict the salt bridge between the charged residues (Arg, Lys, His, Asp and Glu) with a distance ≤4.0 Å.

**Figure 11:**
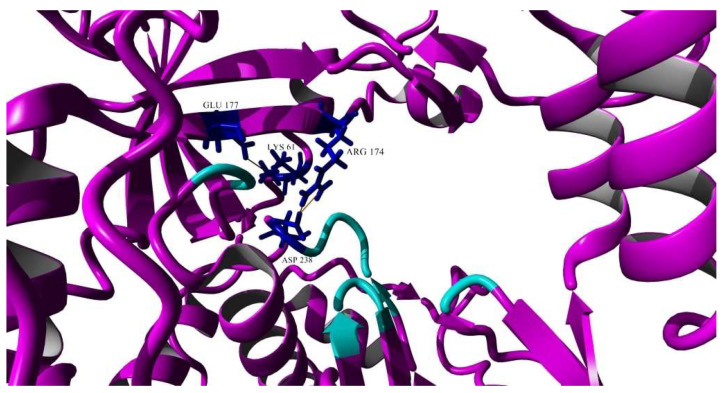
Three additional salt bridges found in the EV-A71/SP 3D polymerase surrounding the active site that could affect the structure and function. A salt bridge formed between Lys61-Glu177 was found in the EV-A71/SP variant. Two salt bridges were formed between Arg174-Asp238 (colored in blue) in the EV-A71/SP variant.

Among the various salt bridges, two of the interactions were found exclusively in the EV-A71/SP variant and they were chosen for further discussion as they were located near the active site and could affect the overall conformation of the 3D^pol^ structure (Figure 11). Two salt bridge interactions were formed between Arg174 and Asp238 and these were of particular interest as Asp238 accounted for one of the amino acids that made up the active site of the 3D^pol^ and was involved in active site closure for catalysis, thus it might directly affect the flexibility of the active site [39] (Figure 11). One of the amino acids that lined the binding pocket, Lys61, demonstrated a salt bridge with Glu177, which was close to the active site (Figure 11).

**Figure 12:**
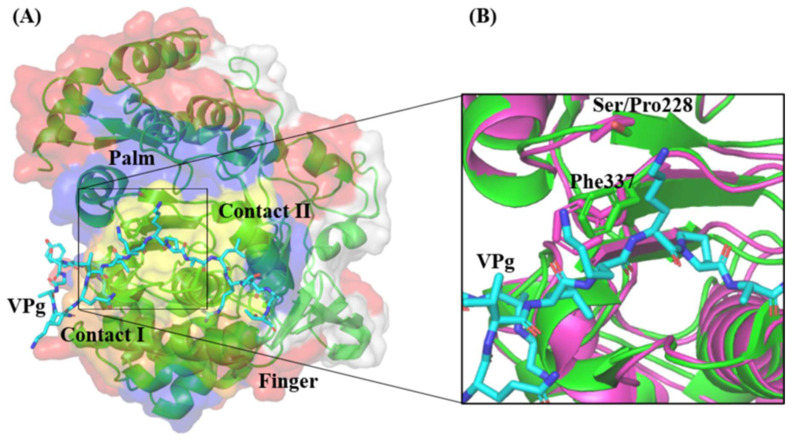
3D polymerase–VPg (virus protein, genome-linked) complex. (**A**) 3D polymerase is shown in cartoon representation (green) and surface, while VPg is shown in sticks (cyan). The amino acids within the palm (blue) and finger (red) domains of 3D polymerase formed an interaction with VPg. These amino acids formed two major regions of the interface named as Contact I (orange) and Contact II (yellow). (**B**) Close-up view of the interface at Phe337. The model of EV-A71/SP was superimposed on the structure to depict the proximity of the S228P mutation to Phe337.

Detailed inspection of the 3D polymerase in the complex with VPg revealed that there were two major regions that could be identified to interact with VPg. These regions are named as Contact I and Contact II. The amino acid Phe337 within Contact II was demonstrated to be critical for 3D polymerase–VPg interaction since its substitution with an alanine residue significantly disrupted the interaction and reduced the uridylylation activity of 3D polymerase by 50% [30]. Interestingly, the mutation S228P found in the EV-A71/SP 3D polymerase was proximal to Phe337 within Contact II (Figure 12). It is possible that this mutation might slightly perturb the structure of the palm domain at Contact II and affect the orientation of Phe337.

## 4. Discussion

The EV-A71 virus uses the RNA-dependent RNA polymerase (RdRp), which lacks proofreading ability as its replication machinery, hence this could lead to high mutation rates, giving rise to genetically unstable strains known as haplotypes in the viral population [6,40]. The high mutation rates of EV-A71 allowed the presence of a quasispecies population in the EV-A71/WT and it was found to consist of six haplotypes through next generation sequencing analysis after reconstruction of the viral strains through CliqueSNV. The six haplotypes differed in genome sequences of the virus, including the 5′-UTR, VP2, VP1, 2A and 3C protein. The EV-A71/WT was subjected to propagation in cell lines and plaque variants emerged with different sizes. Plaque variants were categorized by the different plaque sizes and they were repeatedly purified until homogenous plaques were observed; this was to ensure that there would only be a single haplotype remaining within the viral population. Plaque-to-plaque transfers were shown to reduce fitness of a viral population by presenting it to repeated bottlenecks [40]. Only four plaque variants were successfully isolated displaying different sizes in RD cells (huge, big, medium and small). When the sequences of the four plaque variants were compared against the sequences of the six haplotypes, only the EV-A71/Hap2 haplotype showed the highest similarity to all four plaque variants. The EV-A71/Hap2 harbored amino acid residues at Leu97, Asn237, Pro246 and Thr292 that could possibly act as cell-adapted mutations in RD cells as all four plaque variants derived from EV-A71/Hap2 contained these four mutations. This analysis provided an insight that revealed that the EV-A71/Hap2 was the only cultivable strain within the EV-A71/WT quasispecies population and it is the fittest haplotype to grow in RD cells due to the adaptation. The four plaque variants could have arisen from the EV-A71/Hap2 haplotype due to positive selection, which refers to a certain genotype becoming dominant as a result of positive selection of phenotypic traits expressed by the individual [40]. The selective advantage of a mutant genome could affect the rate of dominance in a population [40]. In this case, despite both EV-A71/Hap0 and EV-A71/Hap1 displaying the highest frequencies of occurrence being detected within the population, they did not become dominant as a result of non-viability in both RD and Vero cell lines. Due to successive plaque-to-plaque transfers during plaque variant purifications, bottleneck events would have occurred and resulted in an accumulation of mutations in the viral genomes, leading to a decrease in fitness level of the non-surviving plaque variants due to selective pressures in the growing environment [40]. Therefore, although the four plaque variants isolated from the RD cells had the highest similarity in genome sequences with EV-A71/Hap2, they carried spontaneous mutations which would have arisen from the passages. On the other hand, as the genome sequences of the plaque variants isolated from Vero cells (EV-A71/VS and EV-A71/VM) showed high similarity to the EV-A71/Hap4 haplotype, EV-A71/Hap4 harboring amino acids at Ile97, Asn104, Thr237, Ser246 and Asn282 were important cell-adapted mutations for survival in Vero cells. The first viral passage of the EV-A71/WT in Vero cells did not produce any plaques. After a number of viral passages, plaque formation was observed and this was related to the phenomenon of natural selection [41], where EV-A71/Hap4 possessed the ability to break through “new host pressures” by adapting to environmental stress, hence gaining fitness for survival in Vero cells. Amino acid sequences of the six haplotypes differed mostly in the VP1 region. It has been reported in many studies that the VP1 protein harbors molecular determinants of virulence [42,43]. The VP1-145 amino acid has been determined as a “hot spot” for evolutionary pressures on EV-A71 [44]. The VP1-145E and 98K/E were responsible for the development of uremia and neuropathogenesis as well as increasing cytokine levels in Cynomolgus monkey models [45]. This was further confirmed by Kobayashi et al. (2018), who reported that VP1-145G showed an attenuated phenotype in wild-type suckling mice and transgenic mice models expressing hSCARB2 as it failed to disseminate well in mouse organs due to absorption to heparin sulfate attachment receptors during circulation. In contrast, VP1-145 E resulted in enhanced viral infections in cell cultures in a heparin sulfate-dependent manner, showing virulent phenotypes in both models [46].

The two plaque variants isolated from Vero cells (EV-A71/VS and EV-A71/VM) acquired mutations in the VP1 region. Annexin II was one of the binding receptors used by EV-A71 and VP1-40-100 was involved in the interactive with annexin II [47]. Both the plaque variants contained an L to I substitution in VP1-97 and this residue was located within the binding region of annexin II. As Vero cells originated from African green monkey (*Chlorocebus aethiops*) kidney cells, there could be subtle differences between the receptors in human and monkey cells. We had performed a comparison of the SCARB2 sequences of humans (hSCARB2) (PDB: 6I2K) and African green monkey (mSCARB2) (GenBank: XP_007997136) using Clustal Omega. Results revealed that there were four amino acid differences and that the amino acid sequence of the mSCARB2 was 62 amino acids longer than the hSCARB2 (Supplementary Material, Data S7). According to Zhou et al. (2019), the α5 (aa.152–163) and α7 (a.183–193) helices of the SCARB2 interacted with the GH loop of VP1 (aa.214–216) and the EF loop of VP2 (aa.134–162) of EV-A71 [48]. The four amino acid differences observed in the mSCARB2 were not in the region of contact with VP1 and VP2. On the other hand, the VP2-T253I and VP1-V138I mutations observed in EV-A71/VS and EV-A71/VM were not within the described residues in the VP1 GH loop and the VP2 EF loop that formed the binding interaction with SCARB2. The results indicated that there were no effects on binding with the hSCARB2 receptor. However, since the amino sequence of the mSCARB2 differed from the hSCARB2, there might be unknown binding regions within EV-A71. For instance, the EV-A71/VS was found to harbor the T253I mutation in the VP2 gene. This mutation could reduce binding towards mSCARB2 and displayed smaller plaque sizes.

In this study, the EV-A71/SP variant isolated from RD cells exhibited the smallest plaque size present in the EV-A71/WT population. Viruses that formed small plaques generally showed lower replication rates, yielding lower amounts of viruses [7,8,9,10,12]. The EV-A71/SP variant displayed slower growth and exhibited lower RNA replication rates when compared with the EV-A71/WT. Significant reductions of viral attachment and internalization into cells would result in an attenuated phenotype with low viral growth and infectivity, as shown in the reduced growth and high TCID_50_ of the EV-A71/SP variant, which indicated that higher amounts of EV-A71/SP variants were needed to cause 50% cytopathic effects in the RD cells. The trend between RNA copy numbers/mL and PFU/mL across the 12, 24, 48 and 72 h.p.i. of the viruses were different. The RNA copy numbers of both the EV-A71/WT and the EV-A71/SP variant increased across the four timepoints, while both viruses reached a peak in the infectivity (PFU/mL) study. This indicated that there could be a presence of defective interfering particles (DIPs). DIPs were virus-like particles containing part of the viral genome but could interfere with replication of the non-defective homologous virus [49]. They could not form matured viral particles [49], and this could account for the reduction in PFUs in later cycles. The high viral copy numbers, as indicated by the RNA replication analysis, revealed that VP1 was increasing at an exponential manner based on the probe targeting VP1. As the VP1 probe could not detect other regions defective in viral genes in the DIPs, any decrease in RNA copy numbers could not be identified, thus suggesting there was a presence of DIPs in the viral supernatant.

A recent study by Gonçalves-Carneiro et al. (2022) had reported that viral replication of EV-A71 could be inhibited by the action of zinc finger antiviral proteins (ZAPs) targeting CpG dinucleotides within the viral genomes [50]. An EV-A71 mutant containing a total of 48 CpG dinucleotides was generated, particularly focusing on the region encompassing amino acid residues D1270-R1586. This region is equivalent to nucleotide numbers 4935-5510 of the EV-A71/WT and the EV-A71/SP variant. Comparisons of synonymous mutations within the EV-A71/SP variant were performed by referring to Supplementary Material, Table S5 and there were no nucleotide substitutions being found within this region, therefore ruling out the fact that slower replication of the EV-A71/SP variant was caused by the action of ZAPs.

Among the mutations found within the EV-A71/SP variant, the substitutions found exclusively in the 2C (S26T) and 3D polymerase (S228P) of the EV-A71/SP were postulated to affect the in vitro virulence of the virus in RD cells. It was reported that the N-terminus of the 2C protein, in particular, amino acid residues 5–43 interacted with reticulon 3 (RTN3) which belongs to a host-encoded 2C binding protein; this was associated with the replication complex due to the RNA and membrane binding ability of the 2C N-terminus [51]. The 25th amino acid of the 2C protein was shown to interact with RTN3 to undertake RNA synthesis [51]. The N-terminus of the 2C protein was shown to harbor functional motifs that were involved in membrane binding (aa.21–54), RNA binding (aa.21–45) and oligomerization (aa.1–38), which could affect viral replication [52]. Since the S26T mutation was localized within the N-terminus of the 2C protein, it could affect RNA replication efficiency and lead to reduced plaque size.

Mutations occurring in the palm region of the RdRp have been shown to alter the elongation rates and in vivo mutation frequencies [24]. In the current investigation, the S228P mutation occurred within the RdRp of the EV-A71/SP variant, which is situated in the palm region of the polymerase. This single mutation in the EV-A71/SP had indirectly caused slight changes in the 3D polymerase active site. This included the RdRp of EV-A71/SP having a shorter sheet (Asp233-Tyr235) and loss of a turn (Gly236-Asp238) within the active site. Peersen (2017) provided an intensive review on the structure, function and fidelity modulation of the RdRp [39]. It was shown that the RdRp active site closure for EV-A71 happened through inducing conformational changes in the palm domain. When a cognate nucleotide was present, a canonical antiparallel ß-sheet was formed between motifs A and C. This interaction was formed through linkage of the hydrogen bond network surrounding Asn297 in motif B towards the NTP 2′-OH of Ser288, followed by a movement of Asp238 to prompt closure of the active site. This led to a re-positioning of Asp233 and prebound metal A Mg^2+^ ion to allow both Asp233 and Asp329 to interact with the two newly yielded magnesium ions within the active site to trigger the catalysis process [53]. The shortened sheet structure in the RdRp of the EV-A71/SP variant was due to the three hydrogen bond interactions located between Asp233-Thr356. The Asp233 was located within the affected sheet and the reposition of this particular amino acid was important for metal ion coordination and effective closure of the RdRp active site for catalysis. On the other hand, Asp238 was responsible for the closure of the active site and it was located within the lost turn structure in the RdRp of the EV-A71/SP variant. Since Asp238 was located in the RdRp of EV-A71/WT, it might contribute to the active site closure when the helix was disrupted and the change in the overall structure of the active site due to the disruption of this helix could possibly affect the overall replication activity.

EV-A71 replication is initiated by the uridylylation of its non-structural protein 3B, also known as VPg [54]. The 3D polymerase plays a key role in this process where it binds to VPg and covalently links VPg to two UMP molecules. The Contact I and II of the 3D polymerase demonstrated the region of contact with VPg (Figure 12). The Phe337 within Contact II is proximal to the mutation of interest, S228P. Chen et al. (2013) reported that Phe337 was vital for the 3D polymerase–VPg interaction, as an alanine substitution at position 337 of the 3D polymerase was shown to reduce the uridylation activity by 50% [30]. Further study is needed to investigate the importance of Ser228 residue on the 3D polymerase interaction with VPg in an EV-A71 replication.

The 3D polymerase of the EV-A71/SP variant also contained a few salt bridges that were not present within the RNA polymerase of the EV-A71/WT. Salt bridges contribute to the rigidity of a protein, thus decreasing the flexibility of the structure [55]. The 3D-S228P mutation had caused a change in conformation of the active site of the 3D polymerase. This could alter the position of the surrounding amino acids, which therefore could affect bond interactions of nearby amino acid residues. The salt bridge formed between Lys61-Glu177 was speculated to affect the replication process, as Lys61 acted as a critical residue through binding to GTP and exerted polymerase activity, utilizing GTP for de novo initiation and elongation of RNA synthesis [56,57]. The formation of this salt bridge could possibly affect the capture of GTP molecules as an initiator nucleotide. Sholders and Peersen (2014) indicated that the disruption of this specific salt bridge released energetic constraints prior to the preparation of catalysis [58]. Therefore, the EV-A71/SP variant containing this salt bridge in the 3D polymerase could not proceed to catalysis. The two additional salt bridges formed between Arg174 and Asp238 in the RdRp of the EV-A71/SP variant could affect protonation in catalysis as Arg174 took part in the pre-catalysis open state. Arg174 belonged to motif F and it indirectly interacted with NTP phosphate via two water molecules. The Arg174 residue might also function as a proton donor to NTP phosphates in order to facilitate enzyme catalysis [53]. Asp238 was involved in an intermediate step placed before the active site closure, during the initial NTP binding event that occurred through a conformation transition termed *in*/*down* [58]. The hydroxyl group of Ser288 would flip downwards facing the active site, thereby forming a hydrogen bond with Asp238, where this interaction played a vital role in rearranging Motif A for catalysis [58]. Asp238 served as a critical residue in the priming and closure of the active site and this might have caused its movement to be affected by the salt bridges [58].

It is important to note that the 3D polymerase might not be the only protein that could account for plaque size differences. The four plaque variants isolated from RD cells were represented by different plaque sizes but the EV-A71/MP, EV-A71/BP and EV-A71/HP did not differ in amino acid sequences in the 3D polymerase. This indicated the possibility of mutations found in other genes in the three plaque variants that could affect plaque size. For instance, since the 5′-UTR-U209C and the VP3-F186L mutations of the EV-A71/MP were unique, they could be responsible for the size of the medium plaque. The 5′-UTR-A99G found in the EV-A71/HP variant could be an important substitution that caused the difference of the big and huge plaque size between EV-A71/BP and EV-A71/HP variants. The 5′-UTR is involved in viral translation and it is essential for virus replication [59]. A nucleotide substitution at position 158 from C to U altered the conformation of the 5′UTR stem loop II RNA secondary structure in a subgenotype B1 strain, resulting in decreased viral translations and reduced virulence in mice [60]. Substitution of U from C at position 115 of 5′UTR in an EV-A71 strain was shown to reduce virulence in 1-day-old BALB/c mice [61]. The VP3 protein is part of the capsid protein and the GH loop (aa.170–192) of the VP3 structure was reported to involve viral uncoating in conjunction with VP1 [62]. The mutations found in the EV-A71/MP and EV-A71/HP variants could be involved in protein translation and viral uncoating (only EV-A71/MP), which affect viral replication processes and hence contribute to the difference in plaque sizes.

Huang et al. (2017) reported the presence of a haplotype that carried a glycine instead of aspartate at VP1-31 [6]. The haplotype containing VP1-31G was able to breakthrough “bottlenecks”, which led to the dissemination of viruses towards the central nervous system. This substitution was found to increase viral adaption and fitness, as a selective bottleneck had shaped the evolutionary pathway, thus resulting in a haplotype switch. Studies by Mandary et al. (2020) revealed the presence of two plaque variants (big and small) in the EV-A71 subgenotype B4 [12]. However, careful examination of the plaque morphology of viruses plated in RD cells indicated the presence of plaques that differed from the two reported. The study also did not provide information on the number of haplotypes within the viral population and the haplotype from which the plaque variants were derived. Our study provided an insight to explain the number of haplotypes in the EV-A71/WT population. However, when subjected to pressures of evolution, only one haplotype (EV-A71/Hap2) was viable in RD cells, but a different haplotype (EV-A71/Hap4) was present in Vero cells. This could indicate that the other haplotypes that were not isolated were due to extinction of the haplotypes within the EV-A71/WT population due to selective pressures.

The molecular basis behind the attenuated phenotype should be studied in order to select a suitable seed strain for live-attenuated vaccines (LAV). The EV-A71/SP variant investigated in this study showed reduced growth and infectivity, which could be due to reduced binding and internalization of the virus. Furthermore, an altered 3D polymerase could also contribute to slow replication. The characteristics were desirable for it to be evaluated further in immunological parameters to act as a LAV. A study by Vignuzzi et al. (2006) revealed the possibility of cooperation interactions within the poliovirus population [63]. Ribavirin and 5-fluoroacil were used for expanding the diversity of the poliovirus population, resulting in some variants that could facilitate entry to the blood–brain barrier, while some facilitated colonization in the gut and others had a role in immune evasion. The EV-A71/WT in our study is an example of a viral population that consisted of a quasispecies structure represented by haplotypes that gave rise to variants with different plaque morphologies. The high in vitro infectivity of the EV-A71/WT population could indicate the complementation effects of variants within the viral population.

## 5. Conclusions

To conclude, the quasispecies structure of a viral population is diverse and the propagation of RNA viruses in different cell lines displayed different variants derived from haplotypes within the viral population. The EV-A71/WT comprises six haplotypes and EV-A71/Hap2 was the only cultivable haplotype in RD cells, indicating that it is the fittest haplotype to adapt to RD cells. On the other hand, EV-A71/Hap4 was able to adapt well in Vero cells and was the only haplotype cultivable in Vero cells. The EV-A71/SP variant was purified and exhibited small-plaque morphology. This small-plaque variant had decreased in vitro virulence in RD cells, reflected through higher TCID_50_, when compared with the wild type. The slower growth of the EV-A71/SP variant was indicated by lower growth kinetics and RNA replication rates. The significant reduction in viral attachment and entry could explain the lower growth rates. In silico predictions have shown that the mutation in the 3D polymerase (S228P) might have contributed to the decrease in in vitro virulence in RD cells. Therefore, further studies could involve the use of site-directed mutagenesis (SDM) to revert this mutation found within the 3D polymerase of the EV-A71/SP variant to that present in the EV-A71/WT. Other spontaneous mutations present in the plaque variants should be further investigated via SDM of specific amino acid residues. This could be further verified by both in vitro and in vivo virulence studies to distinguish true molecular determinants of virulence and cell-adapted mutations. Selecting specific amino acid residues of attenuated viruses would enable the rational design of a stable live-attenuated vaccine (LAV) strain.

## Figures and Tables

**Table 1:** Similarity nucleotide sequences of EV-A71/WT and the six haplotypes.

	EV-A71/WT	Hap0	Hap1	Hap2	Hap3	Hap4	Hap5
**EV-A71/WT**	100.00%	99.76%	99.78%	99.80%	99.78%	99.80%	99.76%
**Hap0**	99.76%	100.00%	99.95%	99.88%	99.95%	99.93%	99.95%
**Hap1**	99.78%	99.95%	100.00%	99.88%	99.97%	99.93%	99.95%
**Hap2**	99.80%	99.88%	99.88%	100.00%	99.88%	99.92%	99.88%
**Hap3**	99.78%	99.95%	99.97%	99.88%	100.00%	99.93%	99.95%
**Hap4**	99.80%	99.93%	99.93%	99.92%	99.93%	100.00%	99.93%
**Hap5**	99.76%	99.95%	99.95%	99.88%	99.95%	99.93%	100.00%

**Table 2:** Abundance and nucleotide and amino acid differences found in the six haplotypes.

Wild-Type/ Variants	Frequency	Nucleotide Differences (Amino Acid Differences)														
		5′-UTR		VP4	VP2		VP1							2A		3C
		290	309	800 (aa.18)	1375 (aa.141)	1664 (aa.237)	2730 (aa.97)	2731 (aa.97)	2752 (aa.104)	3151 (aa.237)	3177 (aa.246)	3285 (aa.282)	3315 (aa.292)	3543 (aa.71)	5825 (aa.144)	5939 (aa.182)
EV-A71/WT	-	U	C	A (Ser)	C (Thr)	U (Thr)	C (Leu)	U (Leu)	A (Asn)	C (Thr)	C (Pro)	A (Asn)	A (Thr)	C (His)	A (Gly)	A (Gln)
Hap0	36.78%	•	•	G (Ser)	U (Ile)	A (Thr)	A (Thr)	C (Thr)	•	•	U (Ser)	•	G (Ala)	•	•	•
Hap1	17.07%	•	U	•	•	•	A (Thr)	C (Thr)	•	•	U (Ser)	•	G (Ala)	•	•	•
Hap2	15.83%	•	•	G (Ser)	•	•	•	•	G (Ser)	A (Asn)	•	G (Asp)	•	•	•	•
Hap3	9.89%	C	•	•	•	•	A (Thr)	C (Thr)	•	•	U (Ser)	•	G (Ala)	•	•	•
Hap4	9.64%	•	•	G (Ser)	•	•	A (Ile)	U (Ile)	•	•	U (Ser)	•	•	U (Tyr)	•	•
Hap5	6.86%	•	•	G (Ser)	•	•	A (Thr)	C (Thr)	•	•	U (Ser)	•	G (Ala)	•	G (Gly)	G (Gln)
Unaccounted SNV pairs	3.93%	-	-	-	-	-	-	-	-	-	-	-	-	-	-	-

‘•’ indicate the same nucleotide/amino acid as the EV-A71/WT reference genome. ‘SNV’ indicates single-nucleotide variant. ‘VP’ indicates virion protein.

**Table 3:** Similarity nucleotide sequences of plaque variants EV-A71/SP, EV-A71/MP, EV-A71/BP and EV-A71/HP with all the six haplotypes.

Plaque Variants	Haplotypes					
	EV-A71/Hap0	EV-A71/Hap1	EV-A71/Hap2	EV-A71/Hap3	EV-A71/Hap4	EV-A71/Hap5
**EV-A71/SP**	99.80%	99.82%	99.92%	99.85%	99.84%	99.80%
**EV-A71/MP**	99.82%	99.85%	99.95%	99.88%	99.87%	99.82%
**EV-A71/BP**	99.76%	99.78%	99.82%	99.78%	99.80%	99.76%
**EV-A71/HP**	99.74%	99.77%	99.81%	99.77%	99.78%	99.74%

**Table 4:** Similarity nucleotide sequences of plaque variants EV-A71/VS and EV-A71/VM with all the six haplotypes.

Plaque Variants	Haplotypes					
	EV-A71/Hap0	EV-A71/Hap1	EV-A71/Hap2	EV-A71/Hap3	EV-A71/Hap4	EV-A71/Hap5
**EV-A71/VS**	99.78%	99.81%	99.77%	99.81%	99.82%	99.78%
**EV-A71/VM**	99.72%	99.74%	99.70%	99.74%	99.76%	99.72%

**Table 5:** Comparison analysis of the 3D^pol^ structures between EV-A71/WT and EV-A71/SP.

	EV-A71/WT	EV-A71/SP
**RMSD/Å**	0.00	0.6457
**Hydrogen bond interactions**	390	414
**Helix**	42.0%	40.5%
**Sheet**	15.8%	15.2%
**Turn**	11.0%	12.3%
**Coil**	30.3%	31.2%
**3_10_ helix**	0.9%	0.9%

## Data Availability

The data presented in this study are available in the article.

## Supplementary Materials

The following supporting information can be downloaded at: https://www.mdpi.com/article/10.3390/vaccines11030629/s1, Table S1: Reagents for RT-qPCR determination; Table S2: Designed primers for EV-A71 PCR products; Data S3: Software parameters; Data S4: Raw genome sequencing data of the six haplotypes in EV-A71/WT, four plaque variants isolated from RD cells and two plaque variants isolated from Vero cells; Table S5: Synonymous and non-synonymous mutations of plaque variants in comparison with the EV-A71 strain 41 subgenotype B4 (GenBank: AF316321.2); Table S6: Summary of salt bridge interactions in 3D polymerase of EV-A71/WT and EV-A71/SP. Data S7: Sequences of human SCARB2 (PDB: 6I2K) and African green monkey SCARB2 (GenBank: XP_007997136.2) with its sequence comparisons.
