# Supplementary material for: Insights into In Vitro Adaptation of EV71 and Analysis of Reduced Virulence by In Silico Predictions
Source: Vaccines (Basel). 2023 Mar 11;11(3):629. doi: 10.3390/vaccines11030629 (PMC10059274; doi:10.3390/vaccines11030629)
Supplement: Supplementary file 1 [file vaccines-11-00629-s001.zip › vaccines-2171762-supplementary.pdf]

Supplementary data

Table S1: Reagents for RT-qPCR determination.

Reagent	Volume (µL)	Final concentration
Forward Primer (10 µM)	1	0.25 µM
Reverse Primer (10 µM)	1	0.25 µM
Probe (5 µM)	1	0.125 µM
TaqMan Fast Virus 1-step mastermix	5	-
RNA template	5	-
Molecular Grade water for qPCR	7	-
Total	20	-

Table S2: Designed primers for EV-A71 PCR products.

Primer	Sequence (5' to 3')
**5' UTR Forward**	TTAAAACAGCTGTGGGTTGTAC
**5' UTR Reverse**	GTGGAGCCTTCTGTAGCTGAAT
**VP4-VP2 Forward**	CAATCAAACATGGGCTCACAGG
**VP4-VP2 Reverse**	TGATTCGTTCCTGGTTTGGCT
**VP3 Forward**	GATTTTGACCAAGGGGCAACTC
**VP3 Reverse**	GCTGGAACTTCACCAGTGTCTA
**VP1 Forward**	GCTTACATAATAGCACTAGCGGC
**VP1 Reverse**	GTAGCGAGGTGACGATTAACCA
**2A-2B Forward**	ACTGTGGGTTCATCAAAGTCCA
**2A-2B Reverse**	CTGCTGGAACAATCTTCTCCCT
**2C Forward**	AAGCTGATTTCAGCGCTAGTGA
**2C Reverse**	AATCCAGCCTTGTTCCCTACAG
**3A-3B-3C Forward**	GTCGCAGGTTCTACATGGATTG
**3A-3B-3C Reverse**	AAACAGGGCTTGTTCAAAGTCG
**3D 3' UTR Forward**	AAGAGGAGCTACTTTGCGAG
**3D 3' UTR Reverse**	GCTATTCCGGTTATAACAAATTTACC
**3602 Forward**	CCCTGCTAGATACCAATCGCA
**6615 Forward**	CCT GGG TCA CTC TTT GCC TT

Data S3: Software parameters.

BBDuk

Trim reads below the threshold value (`trimq`) = 20

Adapter sequences (`ref`) = adapters.fa

Discard reads with length below the threshold value (`minlen`) = 35

Trim the right side (`qtrim`) = r

Trim kmer and all the bases to the right (`ktrim`) = r Kmers (`k`) = 23

Minimum kmers (`mink`) = 9

Hamming distance = (`hdist`) = 1

Trim both reads to the same length (`tpe`)

Trim adapters based on pair overlap detection (`tbo`)

BBMap

Kmers (`k`) = 15

Maximum length of insertions and deletions (`maxindel`) = 100

Bans indel longer than maxindel (`strictmaxindel`) = t

Mark read pairs with low mapping quality as unmapped (`killbadpairs`) = t

Treat unpaired reads as unmapped (`pairedonly`) = t

Do not analyse or print more than this many alignments per read (`maxsites2`) = 80

Minimum alignment identity to look for (`minid`) = 0.95

Sets exact minimum identity allowed for alignments to be printed (`idfilter`) = 0.95

SortSam

Sort order of output file required. Possible values:{unsorted, queryname, coordinate, duplicate} (SORT_ORDER) = coordinate

Freebayes

Ploidy per sample (`-p`) = 1

Exclude alignments from analysis if they have a mapping less than –min-mapping-quality (`--min-mapping-quality`) = 20

Require at least this coverage to process a site (`--min-coverage`) = 3

Use mapping quality of alleles when calculating data likelihood (`--use-mapping-quality`)

CliqueSNV

Run CliqueSNV with Illumina input (`snv-illumina`)

Data S4: Raw genome sequencing data of the six haplotypes in EV-A71/WT, four plaque variants isolated from RD cells and two plaque variants isolated for Vero cells.

```
>EV-A71/Hap0
TTAAAACAGCTGTGGGTTGTACCCACTCACAGGGCCCACGTGGCGCTAGCACTCTGGTTCTGCGGAACCTT
TGTGCGCCTGTTTTACGCCCCCCCCCAATTTGCAACTTAGAAGCAATACACAACACTGATCAACAGCAGG
CATGGCGCACCAGCTATGTCTTGATCAAGCACTTCTGTTTCCCCGGCCGAGTATCAATAGACTGTTCACG
CGGTTGAAGGAGAAAGCGCCCGTTATCCGGCTAACTACTTCGAGAAACCTAGTAGCACCATTGAAGCTGCA
GAGTGTTTCGCTCGGCACTTCCCCCGTGTAGATCAGGTCGATGAGTCACTGCAATCCCCACGGGCGACCGT
GGCAGTGGCTGCGCTGGCGGCCTGCCTATGGGGCAACCCATAGGACGCTCTAATGTGGACATGGTGCGAAG
AGTCTATTGAGCTAGTTAGTAGTCCTCCGGCCCCTGAATGCGGCTAATCCTAACTGTGGAGCACATGCCTT
CAATCCAGAGGGTAGTGTGTCGTAATGGGCAACTCTGCAGCGGAACCGACTACTTTGGGTGTCCGTGTTTC
CTTTTATCTTTACATTGGCTGCTTATGGTGACGATTATAGAATTGTTACCATATAGCTATTGGATTGGCCA
TCCGGTGTGCAATAGAGCTATTATATACCTGTTTGTTGGCTTTGTACCACTAACCTTAAAATCTATAACCA
CCCTCGATTTTATATTAACCCTCAATACAATCAAACATGGGCTCACAGGTGTCTACTCAGCGATCCGGCTC
CCACGAGAACTCCAATTCGGCTACAGAAGGCTCCACCATTAATTACACTACCATCAACTATTACAAAGACT
CCTATGCTGCGACAGCGGGCAAACAGAGCCTCAAGCAAGACCCTGATAAATTTGCTAACCCTGTCAAGGAC
ATTTTCACTGAAATGGCTGCACCACTGAAGTCTCCATCCGCTGAGGCTTGTGGTTACAGTGATCGCGTGGC
ACAACTCACCATTGGAAACTCCACCATCACTACACAGGAGGCGGCGAATATCATAGTCGGTTATGGTGAGT
GGCCCTCATACTGCTCTGATGACGATGCTACAGCGGTGGACAAGCCAACGCGCCCAGATGTTTCAGTGAAT
AGGTTTTATACGTTGGATACTAAATTGTGGGAAAAGTCATCCAAGGGGTGGTATTGGAAGTTTCCTGATGT
ACTGACTGAGACCGGAGTCTTTGGCCAGAATGCACAGTTTCACTATTTATATAGGTCAGGATTTTGCATTC
ATGTGCAATGTAATGCTAGCAAGTTCCATCAAGGAGCGTTGTTAGTCGCCATACTTCCAGAGTATGTTATA
GGGACAGTGGCAGGCGGCACAGGAATTGAGGACAGCCACCCTCCTTACAAACAAACACAACCTGGCGCCGA
TGGTTTTGAGTTGCAGCACCCGTACGTACTCGATGCTGGGATTCCTATATCACAATTAACAGTGTGCCCCC
ACCAATGGATTAACCTACGGACCAATAACTGTGCCACAATAATAGTGCCGTATATGAACACACTGCCTTTC
GACTCTGCCCTGAACCATTGCAACTTTGGGCTGTTGGTGGTGCCCATTAGCCCACTAGATTTTGACCAAGG
GGCAACTCCGGTTATCCCTATTACAATCACACTAGCTCCAATGTGCTCTGAGTTTGCAGGTCTCAGACAGG
CGGTCACTCAAGGTTTTCCCACCGAGCCAAAACCAGGAACGAATCAATTTTTGACCACCGATGACGGTGTC
TCAGCACCCATTTTACCAAATTTCCACCCCACACCATGTATTCACATACCCGGTGAAGTCAGAAACCTGCT
TGAGTTGTGTCAAGTGGAGACCATTCTTGAGGTTAACAATGTACCCACCAATGCCACCAGTCTGATGGAAA
GGCTACGATTCCCGGTGTCCGCGCAAGCGGGAAAAGGTGAATTGTGTGCCGTGTTTAGGGCCGACCCTGGA
AGAGACGGTCCATGGCAATCAACAATGCTGGGCCAGTTGTGTGGATATTACACCCAGTGGTCAGGATCACT
GGAGGTTACTTTTATGTTCACCGGGTCTTTCATGGCCACGGGTAAAATGCTCATAGCTTATACACCTCCTG
GTGGCCCCTTACCCAAAGATCGGGCCACAGCAATGCTGGGCACACATGTTATCTGGGATTTTGGGCTACAA
TCATCTGTCACCCTTGTAATACCATGGATTAGCAACACCCACTACAGAGCGCATGCCCGGGATGGAGTGTT
CGATTACTATACCACAGGACTGGTTAGTATCTGGTATCAAACAAACTACGTGGTTCCAATTGGGGCACCCA
ACACAGCTTACATAATAGCACTAGCGGCAGCCCAGAAGAATTTTACCATGAAACTGTGTAAAGACACCAGT
CACATATTACAGACAGCCTCTATTCAGGGAGATAGAGTGGCAGATGTGATAGAGAGCTCTATAGGAGATAG
TGTGAGTAGGGCACTTACCCAGGCCCTGCCAGCTCCAACAGGTCAGAACACGCAGGTGAGCAGTCATCGAC
TAGACACTGGTGAAGTTCCAGCGCTCCAAGCTGCTGAAATAGGGGCATCGTCAAATACTAGTGATGAGAGT
ATGATTGAGACACGATGCGTTCTTAATTCACACAGTACGGCAGAGACCACCCTGGACAGCTTCTTCAGTAG
GGCAGGCTTGGTAGGAGAGATAGATCTCCCTACTGAGGGTACCACTAATCCAAATGGTTATGCTAATTGGG
ATATAGACATAACTGGTTACGCACAAATGCGCAGGAAAGTGGAGCTGTTCACCTACATGCGCTTTGATGCG
GAATTCACTTTTGTTGCGTGCACTCCTACTGGTCAGGTTGTCCCACAATTACTTCAGTATATGTTTGTTCC
CCCTGGTGCTCCCAAACCAGAGTCTAGAGAATCACTTGCTTGGCAGACAGCCACAAACCCCTCAGTTTTTG
TCAAGTTGACTGATCCCCCGGCACAGGTCTCAGTTCCGTTCATGTCACCCGCGAGCGCTTACCAGTGGTTT
TACGACGGGTACCCCACGTTTGGAGAACACAAACAGGAGAAAGACCTTGAGTATGGAGCGTGCCCTAATAA
TATGATGGGCACTTTCTCGGTGCGAACTGTGGGTTCATCAAAGTCCAAGTATTCTTTGGTTGTCAGGATAT
ATATGAGAATGAAGCATGTCAGGGCGTGGATACCTCGCCCGATGCGCAACCAAAACTACCTGTTTAAAGCC
AATCCAAACTATGCCGGTAACTCCATCAAACCGACCGGCACTAGTCGTGCTGCCATTACTACCCTTGGAAA
GTTCGGCCAGCAATCTGGGGCCATCTACGTGGGCAACTTCAGAGTGGTTAATCGTCACCTCGCTACTCATA
ATGACTGGGCGAACCTCGTCTGGGAAGATAGCTCCCGCGACCTATTAGTGTCGTCTACCACCGCCCAGGGC
TGTGATACAATTGCACGTTGTGACTGTCAAACAGGAGTGTACTATTGTAATTCCAAAAGAAAGCACTATCC
AGTCAGCTTCTCCAAACCCAGCCTCATATATGTGGAGGCTAGCGAGTATTACCCTGCTAGATACCAATCGC
ACCTGATGCTTGCAGCAGGCCACTCTGAGCCCGGCGACTGCGGGGGCATCTTAAGGTGTCAACATGGTGTA
GTTGGTATAGTGTCCACGGGTGGCAACGGGCTCGTTGGTTTTGCTGATGTGAGGGATCTCTTGTGGTTGGA
TGAAGAGGCCATGGAGCAAGGTGTGTCTGACTACATTAAGGGGCTCGGTGACGCATTTGGAACAGGTTTCA
```

CTGATGCTGTATCCAGGGAAGTTGAAGCCCTCAGGAACCACCTCATAGGATCTGATGGAGCAGTGGAAAAA
ATCCTAAAGAACCTTATTAAGCTGATTTCAGCGTTAGTAATTGTGATTAGGAGCGATTATGATATGGTCAC
CCTCACAGCAACTTTAGCCCTGATTGGTTGTCATGGAAGTCCCTGGGCTTGGATTAAAGCCAAAACAGCAT
CCATTTTAGGTATCCCCATCGCCCAGAAGCAGAGCGCTTCTTGGCTAAAGAAATTTAATGATATGGCGAGT
GCTGCCAAGGGTTTAGAATGGATATCCAACAAAATTAGTAAGTTCATTGACTGGCTCAGGGAGAAGATTGT
TCCAGCAGCTAAAGAGAAAGCAGAATTTTTAACCAATTTGAAGCAATTACCACTATTAGAGAACCAGATCA
CGAACTTGGAGCAGTCCGCTGCCTCGCAAGAGGACCTTGAAGCTATGTTTGGGAATGTGTCATACCTCGCC
CATTTCTGTCGCAAGTTCCAACCATTATACGCCACGGAGGCCAAGCGAGTCTATGTTCTAGAGAAGAGAAT
GAACAATTACATGCAGTTCAAGAGCAAACACCGTATTGAACCTGTATGTCTCATCATTAGAGGCTCACCAG
GCACTGGAAAGTCCCTTGCGACCGGCATCATTGCCCGGGCCATAGCAGACAAGTACCACTCTAGTGTGTAC
TCACTCCCACCGGATCCTGACCATTTTGACGGGTACAAACAGCAAGTGGTTACAGTTATGGATGACCTGTG
CCAGAATCCTGACGGCAAAGACATGTCATTATTTTGCCAGATGGTATCCACCGTGGATTTTATTCCACCAA
TGGCTTCTCTCGAAGAAAAGGGAGTTTCTTTCACATCTAAATTTGTTATCGCATCCACCAACGCCAGCAAC
ATTATAGTGCCCACAGTGTCTGACTCTGACGCCATTCGTCGCAGGTTCTACATGGATTGCGACATTGAGGT
CACAGACTCATACAAAACAGACTTGGGTAGACTAGACGCTGGGCGGGCTGCTAAGTTATGCTCTGAAAACA
ACACCGCAAATTTCAAACGATGCAGCCCACTAGTGTGTGGGAAAGCTATTCAACTTAGAGACAGGAAATCC
AAGGTCAGGTATAGCGTGGACACAGTGGTCTCTGAACTTATTAGAGAATACAATAGCAGATCCGCTATTGG
TAACACAATTGAAGCATTATTCCAAGGCCCACCCAAGTTCAGGCCAATAAGGATCAGTCTTGAGGAGAAGC
CAGCCCCAGACGCTATTAGCGATCTCCTTGCTAGTGTGGATAGCGAGGAAGTGCGCCAATACTGTAGGGAA
CAAGGCTGGATTATCCCTGAAACTCCCACCAATGTTGAACGACATCTTAATAGAGCAGTGCTAGTCGTGCA
ATCCATCACTACTGTGGTGGCAGTCGTCTCACTGGTGTACGTCATTTACAAGCTCTTTGCGGGGTTTCAAG
GTGCGTATTCTGGAGCTCCCAAGCAAGTGCTCAAGAAGCCTGTCCTCCGCACGGCAACAGTGCAGGGTCCA
AGCCTTGATTTTGCCCTATCCTTGCTGAGGAGGAACATCAGGCAAGTCCAAACAGACCAAGGGCATTTTAC
CATGTTGGGTGTCAGGGATCGCCTGGCTGTTCTCCCGCGGCACTCACAGCCCGGGAAGACTATTTGGGTGG
AACACAAACTTGTGAACATCCTTGATGCAGTCGAGCTGGTGGACGAGCAGGGCGTTAATTTGGAACTCACA
TTGGTGACACTAGATATTAATGAAAAATTTAGAGATATCACCAAGTTCATTCCAGAGACCATTAGCGGCGC
TAGTGATGCAACTCTAGTGATCAACACAGAACATATGCCGTCAATGTTTGTCCCTGTGGGGGACGTCGTGC
AGTACGGGTTCTTGAACCTCAGTGGAAAGCCAACACATAGGACCATGATGTACAATTTCCCTACAAAAGCA
GGACAGTGTGGAGGCGTGGTTACATCAGTCGGTAAGATTGTTGGTATTCACATTGGTGGCAACGGGCGCCA
AGGGTTCTGCGCTGGTTTGAAGAGGAGCTACTTTGCGAGTATGCAAGGTGAGATCCAATGGGTGAAGCCTA
ACAAGGAAACTGGCAGACTAAACATCAATGGACCAACTCGCACTAAGTTGGAGCCTAGTGTATTTCATGAT
GTGTTTGAAGGCAACAAGGAACCAGCAGTTTTAACAAGTAAAGACCCTAGATTGGAGGTCGACTTTGAACA
AGCCCTGTTTTCCAAGTATGTGGGCAATGTTTTACACGAGCCCGATGAATATGTGACTCAAGCTGCCCTCC
ACTATGCGAATCAACTTAAACAATTGGACATAAACACTAGCAAGATGAGCATGGAGGAAGCGTGCTATGGC
ACTGAAAACCTGGAAGCAATAGACCTCTGCACTAGTGCTGGGTATCCATACAGTGCCCTTGGTATCAAGAA
AAGAGACATTCTCGACCCCATAACCAGGGATGTGTCTAAGATGAAATTCTACATGGATAAATACGGACTAG
ATCTGCCATACTCTACCTATGTGAAGGATGAACTTAGATCTCTGGATAAAATCAAGAAAGGAAAGTCACGC
CTGATAGAGGCCAGCAGCTTGAATGACTCTGTCTACCTCAGAATGACTTTTGGGCACCTTTACGAGGTGTT
TCATGCTAACCCTGGTACTGTGACTGGCTCAGCAGTAGGTTGCAACCCAGACGTGTTTTGGAGTAAACTAC
CGATTCTGCTGCCTGGGTCACTCTTTGCCTTTGACTACTCAGGATATGATGCTAGTCTCAGCCCGGTATGG
TTCAGGGCTCTAGAAGTTGTGTTACGGGAGATTGGGTATTCAGAGGAGGCCGTGTCCCTAATAGAAGGAAT
CAACCACACCCACCATGTGTACCGGAATAAAACATACTGTGTACTTGGTGGGATGCCCTCAGGGTGCTCTG
GTACTTCCATCTTCAATTCAATGATCAACAACATCATCATTAGAACCCTTTTGATCAAAACCTTTAAGGGA
ATAGACCTGGATGAGTTGAACATGGTGGCCTATGGGGACGATGTGCTGGCCAGTTACCCTTTTCCTATTGA
TTGCCTTGAATTGGCTAAGACTGGCAAAGAGTATGGTTTGACCATGACTCCTGCAGACAAATCACCCTGTT
TCAATGAAGTAACATGGGAGAATGCTACCTTCCTGAAGAGAGGGTTCTTGCCAGACCACCAATTTCCATTC
TTAATTCACCCTACGATGCCCATGAGAGAGATCCATGAGTCCATTCGATGGACTAAGGACGCGCGTAACAC
CCAGGATCACGTGCGCTCCCTGTGTCTATTGGCATGGCACAATGGTAAGGATGAATATGAAAAGTTTGTGA
GTGCAATTAGATCAGTTCCAGTTGGAAAAGCGTTGGCCATTCCTAACTTTGAGAATCTGAGAAGAAATTGG
CTCGAATTGTTTTAATATTACAGTTTAAAGCTGAACCCCACTAGAAATCTGGTCGTGTTAATGACTAGTGG
GGGTAAATTTGTTATAACCGGAATAGC

>EV-A71/Hap1
TTAAAACAGCTGTGGGTTGTACCCACTCACAGGGCCCACGTGGCGCTAGCACTCTGGTTCTGCGGAACCTT
TGTGCGCCTGTTTTACGCCCCCCCCCCAATTTGCAACTTAGAAGCAATACACAACACTGATCAACAGCAGG
CATGGCGCACCAGCTATGTCTTGATCAAGCACTTCTGTTTCCCCGGGCCGAGTATCAATAGACTGTTCACG
CGGTTGAAGGAGAAAGCGCCCGTTATCCGGCTAACTACTTCGAGAAACCTAGTAGCACCATTGAAGCTGCA
GAGTGTTTCGCTCGGCACTTCCCCTGTGTAGATCAGGTCGATGAGTCACTGCAATCCCCACGGGCGACCGT

GGCAGTGGCTGCGCTGGCGGCCTGCCTATGGGGCAACCCATAGGACGCTCTAATGTGGACATGGTGCGAAG
AGTCTATTGAGCTAGTTAGTAGTCCTCCGGCCCCTGAATGCGGCTAATCCTAACTGTGGAGCACATGCCTT
CAATCCAGAGGGTAGTGTGTCGTAATGGGCAACTCTGCAGCGGAACCGACTACTTTGGGTGTCCGTGTTTC
CTTTTATCTTTACATTGGCTGCTTATGGTGACGATTATAGAATTGTTACCATATAGCTATTGGATTGGCCA
TCCGGTGTGCAATAGAGCTATTATATACCTGTTTGTTGGCTTTGTACCACTAACCTTAAAATCTATAACCA
CCCTCGATTTTATATTAACCCTCAATACAATCAAACATGGGCTCACAGGTGTCTACTCAGCGATCCGGCTC
CCACGAGAACTCCAATTCAGCTACAGAAGGCTCCACCATTAATTACACTACCATCAACTATTACAAAGACT
CCTATGCTGCGACAGCGGGCAAACAGAGCCTCAAGCAAGACCCTGATAAATTTGCTAACCCTGTCAAGGAC
ATTTTCACTGAAATGGCTGCACCACTGAAGTCTCCATCCGCTGAGGCTTGTGGTTACAGTGATCGCGTGGC
ACAACTCACCATTGGAAACTCCACCATCACTACACAGGAGGCGGCGAATATCATAGTCGGTTATGGTGAGT
GGCCCTCATACTGCTCTGATGACGATGCTACAGCGGTGGACAAGCCAACGCGCCCAGATGTTTCAGTGAAT
AGGTTTTATACGTTGGATACTAAATTGTGGGAAAAGTCATCCAAGGGGTGGTATTGGAAGTTTCCTGATGT
ACTGACTGAGACCGGAGTCTTTGGCCAGAATGCACAGTTTCACTATTTATATAGGTCAGGATTTTGCATTC
ATGTGCAATGTAATGCTAGCAAGTTCCATCAAGGAGCGTTGTTAGTCGCCATACTTCCAGAGTATGTTATA
GGGACAGTGGCAGGCGGCACAGGAACTGAGGACAGCCACCCTCCTTACAAACAAACACAACCTGGCGCCGA
TGGTTTTGAGTTGCAGCACCCGTACGTACTCGATGCTGGGATTCCTATATCACAATTAACAGTGTGCCCCC
ACCAATGGATTAACCTACGGACCAATAACTGTGCCACAATAATAGTGCCGTATATGAACACACTGCCTTTC
GACTCTGCCCTGAACCATTGCAACTTTGGGCTGTTGGTGGTGCCCATTAGCCCACTAGATTTTGACCAAGG
GGCAACTCCGGTTATCCCTATTACAATCACTCTAGCTCCAATGTGCTCTGAGTTTGCAGGTCTCAGACAGG
CGGTCACTCAAGGTTTTCCCACCGAGCCAAAACCAGGAACGAATCAATTTTTGACCACCGATGACGGTGTC
TCAGCACCCATTTTACCAAATTTCCACCCCACACCATGTATTCACATACCCGGTGAAGTCAGAAACCTGCT
TGAGTTGTGTCAAGTGGAGACCATTCTTGAGGTTAACAATGTACCCACCAATGCCACCAGTCTGATGGAAA
GGCTACGATTCCCGGTGTCCGCGCAAGCGGGAAAAGGTGAATTGTGTGCCGTGTTTAGGGCCGACCCTGGA
AGAGACGGTCCATGGCAATCAACAATGCTGGGCCAGTTGTGTGGATATTACACCCAGTGGTCAGGATCACT
GGAGGTTACTTTTATGTTCACCGGGTCTTTCATGGCCACGGGTAAAATGCTCATAGCTTATACACCTCCTG
GTGGCCCCTTACCCAAAGATCGGGCCACAGCAATGCTGGGCACACATGTTATCTGGGATTTTGGGCTACAA
TCATCTGTCACCCTTGTAATACCATGGATTAGCAACACCCACTACAGAGCGCATGCCCGGGATGGAGTGTT
CGATTACTATACCACAGGACTGGTTAGTATCTGGTATCAAACAAACTACGTGGTTCCAATTGGGGCACCCA
ACACAGCTTACATAATAGCACTAGCGGCAGCCCAGAAGAATTTTACCATGAAACTGTGTAAAGACACCAGT
CACATATTACAGACAGCCTCTATTCAGGGAGATAGAGTGGCAGATGTGATAGAGAGCTCTATAGGAGATAG
TGTGAGTAGGGCACTTACCCAGGCCCTGCCAGCTCCAACAGGTCAGAACACGCAGGTGAGCAGTCATCGAC
TAGACACTGGTGAAGTTCCAGCGCTCCAAGCTGCTGAAATAGGGGCATCGTCAAATACTAGTGATGAGAGT
ATGATTGAGACACGATGCGTTCTTAATTCACACAGTACGGCAGAGACCACCCTGGACAGCTTCTTCAGTAG
GGCAGGCTTGGTAGGAGAGATAGATCTCCCTACTGAGGGTACCACTAATCCAAATGGTTATGCTAATTGGG
ATATAGACATAACTGGTTACGCACAAATGCGCAGGAAAGTGGAGCTGTTCACCTACATGCGCTTTGATGCG
GAATTCACTTTTGTTGCGTGCACTCCTACTGGTCAGGTTGTCCCACAATTACTTCAGTATATGTTTGTTCC
CCCTGGTGCTCCCAAACCAGAGTCTAGAGAATCACTTGCTTGGCAGACAGCCACAAACCCCTCAGTTTTTG
TCAAGTTGACTGATCCCCCGGCACAGGTCTCAGTTCCGTTCATGTCACCCGCGAGCGCTTACCAGTGGTTT
TACGACGGGTACCCCACGTTTGGAGAACACAAACAGGAGAAAGACCTTGAGTATGGAGCGTGCCCTAATAA
TATGATGGGCACTTTCTCGGTGCGAACTGTGGGTTCATCAAAGTCCAAGTATTCTTTGGTTGTCAGGATAT
ATATGAGAATGAAGCATGTCAGGGCGTGGATACCTCGCCCGATGCGCAACCAAAACTACCTGTTTAAAGCC
AATCCAAACTATGCCGGTAACTCCATCAAACCGACCGGCACTAGTCGTGCTGCCATTACTACCCTTGGAAA
GTTCGGCCAGCAATCTGGGGCCATCTACGTGGGCAACTTCAGAGTGGTTAATCGTCACCTCGCTACTCATA
ATGACTGGGCGAACCTCGTCTGGGAAGATAGCTCCCGCGACCTATTAGTGTCGTCTACCACCGCCCAGGGC
TGTGATACAATTGCACGTTGTGACTGTCAAACAGGAGTGTACTATTGTAATTCCAAAAGAAAGCACTATCC
AGTCAGCTTCTCCAAACCCAGCCTCATATATGTGGAGGCTAGCGAGTATTACCCTGCTAGATACCAATCGC
ACCTGATGCTTGCAGCAGGCCACTCTGAGCCCGGCGACTGCGGGGGCATCTTAAGGTGTCAACATGGTGTA
GTTGGTATAGTGTCCACGGGTGGCAACGGGCTCGTTGGTTTTGCTGATGTGAGGGATCTCTTGTGGTTGGA
TGAAGAGGCCATGGAGCAAGGTGTGTCTGACTACATTAAGGGGCTCGGTGACGCATTTGGAACAGGTTTCA
CTGATGCTGTATCCAGGGAAGTTGAAGCCCTCAGGAACCACCTCATAGGATCTGATGGAGCAGTGGAAAAA
ATCCTAAAGAACCTTATTAAGCTGATTTCAGCGTTAGTAATTGTGATTAGGAGCGATTATGATATGGTCAC
CCTCACAGCAACTTTAGCCCTGATTGGTTGTCATGGAAGTCCCTGGGCTTGGATTAAAGCCAAAACAGCAT
CCATTTTAGGTATCCCCATCGCCCAGAAGCAGAGCGCTTCTTGGCTAAAGAAATTTAATGATATGGCGAGT
GCTGCCAAGGGTTTAGAATGGATATCCAACAAAATTAGTAAGTTCATTGACTGGCTCAGGGAGAAGATTGT
TCCAGCAGCTAAAGAGAAAGCAGAATTTTTAACCAATTTGAAGCAATTACCACTATTAGAGAACCAGATCA
CGAACTTGGAGCAGTCCGCTGCCTCGCAAGAGGACCTTGAAGCTATGTTTGGGAATGTGTCATACCTCGCC
CATTTCTGTCGCAAGTTCCAACCATTATACGCCACGGAGGCCAAGCGAGTCTATGTTCTAGAGAAGAGAAT
GAACAATTACATGCAGTTCAAGAGCAAACACCGTATTGAACCTGTATGTCTCATCATTAGAGGCTCACCAG

GCACTGGAAAGTCCCTTGCGACCGGCATCATTGCCCGGGCCATAGCAGACAAGTACCACTCTAGTGTGTAC
TCACTCCCACCGGATCCTGACCATTTTGACGGGTACAAACAGCAAGTGGTTACAGTTATGGATGACCTGTG
CCAGAATCCTGACGGCAAAGACATGTCATTATTTTGCCAGATGGTATCCACCGTGGATTTTATTCCACCAA
TGGCTTCTCTCGAAGAAAAGGGAGTTTCTTTCACATCTAAATTTGTTATCGCATCCACCAACGCCAGCAAC
ATTATAGTGCCCACAGTGTCTGACTCTGACGCCATTCGTCGCAGGTTCTACATGGATTGCGACATTGAGGT
CACAGACTCATACAAAACAGACTTGGGTAGACTAGACGCTGGGCGGGCTGCTAAGTTATGCTCTGAAAACA
ACACCGCAAATTTCAAACGATGCAGCCCACTAGTGTGTGGGAAAGCTATTCAACTTAGAGACAGGAAATCC
AAGGTCAGGTATAGCGTGGACACAGTGGTCTCTGAACTTATTAGAGAATACAATAGCAGATCCGCTATTGG
TAACACAATTGAAGCATTATTCCAAGGCCCACCCAAGTTCAGGCCAATAAGGATCAGTCTTGAGGAGAAGC
CAGCCCCAGACGCTATTAGCGATCTCCTTGCTAGTGTGGATAGCGAGGAAGTGCGCCAATACTGTAGGGAA
CAAGGCTGGATTATCCCTGAAACTCCCACCAATGTTGAACGACATCTTAATAGAGCAGTGCTAGTCGTGCA
ATCCATCACTACTGTGGTGGCAGTCGTCTCACTGGTGTACGTCATTTACAAGCTCTTTGCGGGGTTTCAAG
GTGCGTATTCTGGAGCTCCCAAGCAAGTGCTCAAGAAGCCTGTCCTCCGCACGGCAACAGTGCAGGGTCCA
AGCCTTGATTTTGCCCTATCCTTGCTGAGGAGGAACATCAGGCAAGTCCAAACAGACCAAGGGCATTTTAC
CATGTTGGGTGTCAGGGATCGCCTGGCTGTTCTCCCGCGGCACTCACAGCCCGGGAAGACTATTTGGGTGG
AACACAAACTTGTGAACATCCTTGATGCAGTCGAGCTGGTGGACGAGCAGGGCGTTAATTTGGAACTCACA
TTGGTGACACTAGATATTAATGAAAAATTTAGAGATATCACCAAGTTCATTCCAGAGACCATTAGCGGCGC
TAGTGATGCAACTCTAGTGATCAACACAGAACATATGCCGTCAATGTTTGTCCCTGTGGGGGACGTCGTGC
AGTACGGGTTCTTGAACCTCAGTGGAAAGCCAACACATAGGACCATGATGTACAATTTCCCTACAAAAGCA
GGACAGTGTGGAGGCGTGGTTACATCAGTCGGTAAGATTGTTGGTATTCACATTGGTGGCAACGGGCGCCA
AGGGTTCTGCGCTGGTTTGAAGAGGAGCTACTTTGCGAGTATGCAAGGTGAGATCCAATGGGTGAAGCCTA
ACAAGGAAACTGGCAGACTAAACATCAATGGACCAACTCGCACTAAGTTGGAGCCTAGTGTATTTCATGAT
GTGTTTGAAGGCAACAAGGAACCAGCAGTTTTAACAAGTAAAGACCCTAGATTGGAGGTCGACTTTGAACA
AGCCCTGTTTTCCAAGTATGTGGGCAATGTTTTACACGAGCCCGATGAATATGTGACTCAAGCTGCCCTCC
ACTATGCGAATCAACTTAAACAATTGGACATAAACACTAGCAAGATGAGCATGGAGGAAGCGTGCTATGGC
ACTGAAAACCTGGAAGCAATAGACCTCTGCACTAGTGCTGGGTATCCATACAGTGCCCTTGGTATCAAGAA
AAGAGACATTCTCGACCCCATAACCAGGGATGTGTCTAAGATGAAATTCTACATGGATAAATACGGACTAG
ATCTGCCATACTCTACCTATGTGAAGGATGAACTTAGATCTCTGGATAAAATCAAGAAAGGAAAGTCACGC
CTGATAGAGGCCAGCAGCTTGAATGACTCTGTCTACCTCAGAATGACTTTTGGGCACCTTTACGAGGTGTT
TCATGCTAACCCTGGTACTGTGACTGGCTCAGCAGTAGGTTGCAACCCAGACGTGTTTTGGAGTAAACTAC
CGATTCTGCTGCCTGGGTCACTCTTTGCCTTTGACTACTCAGGATATGATGCTAGTCTCAGCCCGGTATGG
TTCAGGGCTCTAGAAGTTGTGTTACGGGAGATTGGGTATTCAGAGGAGGCCGTGTCCCTAATAGAAGGAAT
CAACCACACCCACCATGTGTACCGGAATAAAACATACTGTGTACTTGGTGGGATGCCCTCAGGGTGCTCTG
GTACTTCCATCTTCAATTCAATGATCAACAACATCATCATTAGAACCCTTTTGATCAAAACCTTTAAGGGA
ATAGACCTGGATGAGTTGAACATGGTGGCCTATGGGGACGATGTGCTGGCCAGTTACCCTTTTCCTATTGA
TTGCCTTGAATTGGCTAAGACTGGCAAAGAGTATGGTTTGACCATGACTCCTGCAGACAAATCACCCTGTT
TCAATGAAGTAACATGGGAGAATGCTACCTTCCTGAAGAGAGGGTTCTTGCCAGACCACCAATTTCCATTC
TTAATTCACCCTACGATGCCCATGAGAGAGATCCATGAGTCCATTCGATGGACTAAGGACGCGCGTAACAC
CCAGGATCACGTGCGCTCCCTGTGTCTATTGGCATGGCACAATGGTAAGGATGAATATGAAAAGTTTGTGA
GTGCAATTAGATCAGTTCCAGTTGGAAAAGCGTTGGCCATTCCTAACTTTGAGAATCTGAGAAGAAATTGG
CTCGAATTGTTTTAATATTACAGTTTAAAGCTGAACCCCACTAGAAATCTGGTCGTGTTAATGACTAGTGG
GGGTAAATTTGTTATAACCGGAATAGC

>EV-A71/Hap2
TTAAAACAGCTGTGGGTTGTACCCACTCACAGGGCCCACGTGGCGCTAGCACTCTGGTTCTGCGGAACCTT
TGTGCGCCTGTTTTACGCCCCCCCCCCAATTTGCAACTTAGAAGCAATACACAACACTGATCAACAGCAGG
CATGGCGCACCAGCTATGTCTTGATCAAGCACTTCTGTTTCCCCGGGCCGAGTATCAATAGACTGTTCACG
CGGTTGAAGGAGAAAGCGCCCGTTATCCGGCTAACTACTTCGAGAAACCTAGTAGCACCATTGAAGCTGCA
GAGTGTTTCGCTCGGCACTTCCCCCGTGTAGATCAGGTCGATGAGTCACTGCAATCCCCACGGGCGACCGT
GGCAGTGGCTGCGCTGGCGGCCTGCCTATGGGGCAACCCATAGGACGCTCTAATGTGGACATGGTGCGAAG
AGTCTATTGAGCTAGTTAGTAGTCCTCCGGCCCCTGAATGCGGCTAATCCTAACTGTGGAGCACATGCCTT
CAATCCAGAGGGTAGTGTGTCGTAATGGGCAACTCTGCAGCGGAACCGACTACTTTGGGTGTCCGTGTTTC
CTTTTATCTTTACATTGGCTGCTTATGGTGACGATTATAGAATTGTTACCATATAGCTATTGGATTGGCCA
TCCGGTGTGCAATAGAGCTATTATATACCTGTTTGTTGGCTTTGTACCACTAACCTTAAAATCTATAACCA
CCCTCGATTTTATATTAACCCTCAATACAATCAAACATGGGCTCACAGGTGTCTACTCAGCGATCCGGCTC
CCACGAGAACTCCAATTCGGCTACAGAAGGCTCCACCATTAATTACACTACCATCAACTATTACAAAGACT
CCTATGCTGCGACAGCGGGCAAACAGAGCCTCAAGCAAGACCCTGATAAATTTGCTAACCCTGTCAAGGAC
ATTTTCACTGAAATGGCTGCACCACTGAAGTCTCCATCCGCTGAGGCTTGTGGTTACAGTGATCGCGTGGC

ACAACTCACCATTGGAAACTCCACCATCACTACACAGGAGGCGGCGAATATCATAGTCGGTTATGGTGAGT
GGCCCTCATACTGCTCTGATGACGATGCTACAGCGGTGGACAAGCCAACGCGCCCAGATGTTTCAGTGAAT
AGGTTTTATACGTTGGATACTAAATTGTGGGAAAAGTCATCCAAGGGGTGGTATTGGAAGTTTCCTGATGT
ACTGACTGAGACCGGAGTCTTTGGCCAGAATGCACAGTTTCACTATTTATATAGGTCAGGATTTTGCATTC
ATGTGCAATGTAATGCTAGCAAGTTCCATCAAGGAGCGTTGTTAGTCGCCATACTTCCAGAGTATGTTATA
GGGACAGTGGCAGGCGGCACAGGAACTGAGGACAGCCACCCTCCTTACAAACAAACACAACCTGGCGCCGA
TGGTTTTGAGTTGCAGCACCCGTACGTACTCGATGCTGGGATTCCTATATCACAATTAACAGTGTGCCCCC
ACCAATGGATTAACCTACGGACCAATAACTGTGCCACAATAATAGTGCCGTATATGAACACACTGCCTTTC
GACTCTGCCCTGAACCATTGCAACTTTGGGCTGTTGGTGGTGCCCATTAGCCCACTAGATTTTGACCAAGG
GGCAACTCCGGTTATCCCTATTACAATCACTCTAGCTCCAATGTGCTCTGAGTTTGCAGGTCTCAGACAGG
CGGTCACTCAAGGTTTTCCCACCGAGCCAAAACCAGGAACGAATCAATTTTTGACCACCGATGACGGTGTC
TCAGCACCCATTTTACCAAATTTCCACCCCACACCATGTATTCACATACCCGGTGAAGTCAGAAACCTGCT
TGAGTTGTGTCAAGTGGAGACCATTCTTGAGGTTAACAATGTACCCACCAATGCCACCAGTCTGATGGAAA
GGCTACGATTCCCGGTGTCCGCGCAAGCGGGAAAAGGTGAATTGTGTGCCGTGTTTAGGGCCGACCCTGGA
AGAGACGGTCCATGGCAATCAACAATGCTGGGCCAGTTGTGTGGATATTACACCCAGTGGTCAGGATCACT
GGAGGTTACTTTTATGTTCACCGGGTCTTTCATGGCCACGGGTAAAATGCTCATAGCTTATACACCTCCTG
GTGGCCCCTTACCCAAAGATCGGGCCACAGCAATGCTGGGCACACATGTTATCTGGGATTTTGGGCTACAA
TCATCTGTCACCCTTGTAATACCATGGATTAGCAACACCCACTACAGAGCGCATGCCCGGGATGGAGTGTT
CGATTACTATACCACAGGACTGGTTAGTATCTGGTATCAAACAAACTACGTGGTTCCAATTGGGGCACCCA
ACACAGCTTACATAATAGCACTAGCGGCAGCCCAGAAGAATTTTACCATGAAACTGTGTAAAGACACCAGT
CACATATTACAGACAGCCTCTATTCAGGGAGATAGAGTGGCAGATGTGATAGAGAGCTCTATAGGAGATAG
TGTGAGTAGGGCACTTACCCAGGCCCTGCCAGCTCCAACAGGTCAGAACACGCAGGTGAGCAGTCATCGAC
TAGACACTGGTGAAGTTCCAGCGCTCCAAGCTGCTGAAATAGGGGCATCGTCAAATACTAGTGATGAGAGT
ATGATTGAGACACGATGCGTTCTTAATTCACACAGTACGGCAGAGACCACCCTGGACAGCTTCTTCAGTAG
GGCAGGCTTGGTAGGAGAGATAGATCTCCCTCTTGAGGGTACCACTAATCCAAGTGGTTATGCTAATTGGG
ATATAGACATAACTGGTTACGCACAAATGCGCAGGAAAGTGGAGCTGTTCACCTACATGCGCTTTGATGCG
GAATTCACTTTTGTTGCGTGCACTCCTACTGGTCAGGTTGTCCCACAATTACTTCAGTATATGTTTGTTCC
CCCTGGTGCTCCCAAACCAGAGTCTAGAGAATCACTTGCTTGGCAGACAGCCACAAACCCCTCAGTTTTTG
TCAAGTTGACTGATCCCCCGGCACAGGTCTCAGTTCCGTTCATGTCACCCGCGAGCGCTTACCAGTGGTTT
TACGACGGGTACCCCACGTTTGGAGAACACAAACAGGAGAAAGACCTTGAGTATGGAGCGTGCCCTAATAA
TATGATGGGCACTTTCTCGGTGCGAAATGTGGGTTCATCAAAGTCCAAGTATCCTTTGGTTGTCAGGATAT
ATATGAGAATGAAGCATGTCAGGGCGTGGATACCTCGCCCGATGCGCAACCAAAACTACCTGTTTAAAGCC
AATCCAAACTATGCCGGTGACTCCATCAAACCGACCGGCACTAGTCGTACTGCCATTACTACCCTTGGAAA
GTTCGGCCAGCAATCTGGGGCCATCTACGTGGGCAACTTCAGAGTGGTTAATCGTCACCTCGCTACTCATA
ATGACTGGGCGAACCTCGTCTGGGAAGATAGCTCCCGCGACCTATTAGTGTCGTCTACCACCGCCCAGGGC
TGTGATACAATTGCACGTTGTGACTGTCAAACAGGAGTGTACTATTGTAATTCCAAAAGAAAGCACTATCC
AGTCAGCTTCTCCAAACCCAGCCTCATATATGTGGAGGCTAGCGAGTATTACCCTGCTAGATACCAATCGC
ACCTGATGCTTGCAGCAGGCCACTCTGAGCCCGGCGACTGCGGGGGCATCTTAAGGTGTCAACATGGTGTA
GTTGGTATAGTGTCCACGGGTGGCAACGGGCTCGTTGGTTTTGCTGATGTGAGGGATCTCTTGTGGTTGGA
TGAAGAGGCCATGGAGCAAGGTGTGTCTGACTACATTAAGGGGCTCGGTGACGCATTTGGAACAGGTTTCA
CTGATGCTGTATCCAGGGAAGTTGAAGCCCTCAGGAACCACCTCATAGGATCTGATGGAGCAGTGGAAAAA
ATCCTAAAGAACCTTATTAAGCTGATTTCAGCGTTAGTAATTGTGATTAGGAGCGATTATGATATGGTCAC
CCTCACAGCAACTTTAGCCCTGATTGGTTGTCATGGAAGTCCCTGGGCTTGGATTAAAGCCAAAACAGCAT
CCATTTTAGGTATCCCCATCGCCCAGAAGCAGAGCGCTTCTTGGCTAAAGAAATTTAATGATATGGCGAGT
GCTGCCAAGGGTTTAGAATGGATATCCAACAAAATTAGTAAGTTCATTGACTGGCTCAGGGAGAAGATTGT
TCCAGCAGCTAAAGAGAAAGCAGAATTTTTAACCAATTTGAAGCAATTACCACTATTAGAGAACCAGATCA
CGAACTTGGAGCAGTCCGCTGCCTCGCAAGAGGACCTTGAAGCTATGTTTGGGAATGTGTCATACCTCGCC
CATTTCTGTCGCAAGTTCCAACCATTATACGCCACGGAGGCCAAGCGAGTCTATGTTCTAGAGAAGAGAAT
GAACAATTACATGCAGTTCAAGAGCAAACACCGTATTGAACCTGTATGTCTCATCATTAGAGGCTCACCAG
GCACTGGAAAGTCCCTTGCGACCGGCATCATTGCCCGGGCCATAGCAGACAAGTACCACTCTAGTGTGTAC
TCACTCCCACCGGATCCTGACCATTTTGACGGGTACAAACAGCAAGTGGTTACAGTTATGGATGACCTGTG
CCAGAATCCTGACGGCAAAGACATGTCATTATTTTGCCAGATGGTATCCACCGTGGATTTTATTCCACCAA
TGGCTTCTCTCGAAGAAAAGGGAGTTTCTTTCACATCTAAATTTGTTATCGCATCCACCAACGCCAGCAAC
ATTATAGTGCCCACAGTGTCTGACTCTGACGCCATTCGTCGCAGGTTCTACATGGATTGCGACATTGAGGT
CACAGACTCATACAAAACAGACTTGGGTAGACTAGACGCTGGGCGGGCTGCTAAGTTATGCTCTGAAAACA
ACACCGCAAATTTCAAACGATGCAGCCCACTAGTGTGTGGGAAAGCTATTCAACTTAGAGACAGGAAATCC
AAGGTCAGGTATAGCGTGGACACAGTGGTCTCTGAACTTATTAGAGAATACAATAGCAGATCCGCTATTGG
TAACACAATTGAAGCATTATTCCAAGGCCCACCCAAGTTCAGGCCAATAAGGATCAGTCTTGAGGAGAAGC

CAGCCCCAGACGCTATTAGCGATCTCCTTGCTAGTGTGGATAGCGAGGAAGTGCGCCAATACTGTAGGGAA
CAAGGCTGGATTATCCCTGAAACTCCCACCAATGTTGAACGACATCTTAATAGAGCAGTGCTAGTCGTGCA
ATCCATCACTACTGTGGTGGCAGTCGTCTCACTGGTGTACGTCATTTACAAGCTCTTTGCGGGGTTTCAAG
GTGCGTATTCTGGAGCTCCCAAGCAAGTGCTCAAGAAGCCTGTCCTCCGCACGGCAACAGTGCAGGGTCCA
AGCCTTGATTTTGCCCTATCCTTGCTGAGGAGGAACATCAGGCAAGTCCAAACAGACCAAGGGCATTTTAC
CATGTTGGGTGTCAGGGATCGCCTGGCTGTTCTCCCGCGGCACTCACAGCCCGGGAAGACTATTTGGGTGG
AACACAAACTTGTGAACATCCTTGATGCAGTCGAGCTGGTGGACGAGCAGGGCGTTAATTTGGAACTCACA
TTGGTGACACTAGATATTAATGAAAAATTTAGAGATATCACCAAGTTCATTCCAGAGACCATTAGCGGCGC
TAGTGATGCAACTCTAGTGATCAACACAGAACATATGCCGTCAATGTTTGTCCCTGTGGGGGACGTCGTGC
AGTACGGGTTCTTGAACCTCAGTGGAAAGCCAACACATAGGACCATGATGTACAATTTCCCTACAAAAGCA
GGACAGTGTGGAGGCGTGGTTACATCAGTCGGTAAGATTGTTGGTATTCACATTGGTGGCAACGGGCGCCA
AGGGTTCTGCGCTGGTTTGAAGAGGAGCTACTTTGCGAGTATGCAAGGTGAGATCCAATGGGTGAAGCCTA
ACAAGGAAACTGGCAGACTAAACATCAATGGACCAACTCGCACTAAGTTGGAGCCTAGTGTATTTCATGAT
GTGTTTGAAGGCAACAAGGAACCAGCAGTTTTAACAAGTAAAGACCCTAGATTGGAGGTCGACTTTGAACA
AGCCCTGTTTTCCAAGTATGTGGGCAATGTTTTACACGAGCCCGATGAATATGTGACTCAAGCTGCCCTCC
ACTATGCGAATCAACTTAAACAATTGGACATAAACACTAGCAAGATGAGCATGGAGGAAGCGTGCTATGGC
ACTGAAAACCTGGAAGCAATAGACCTCTGCACTAGTGCTGGGTATCCATACAGTGCCCTTGGTATCAAGAA
AAGAGACATTCTCGACCCCATAACCAGGGATGTGTCTAAGATGAAATTCTACATGGATAAATACGGACTAG
ATCTGCCATACTCTACCTATGTGAAGGATGAACTTAGATCTCTGGATAAAATCAAGAAAGGAAAGTCACGC
CTGATAGAGGCCAGCAGCTTGAATGACTCTGTCTACCTCAGAATGACTTTTGGGCACCTTTACGAGGTGTT
TCATGCTAACCCTGGTACTGTGACTGGCTCAGCAGTAGGTTGCAACCCAGACGTGTTTTGGAGTAAACTAC
CGATTCTGCTGCCTGGGTCACTCTTTGCCTTTGACTACTCAGGATATGATGCTAGTCTCAGCCCGGTATGG
TTCAGGGCTCTAGAAGTTGTGTTACGGGAGATTGGGTATTCAGAGGAGGCCGTGTCCCTAATAGAAGGAAT
CAACCACACCCACCATGTGTACCGGAATAAAACATACTGTGTACTTGGTGGGATGCCCTCAGGGTGCTCTG
GTACTTCCATCTTCAATTCAATGATCAACAACATCATCATTAGAACCCTTTTGATCAAAACCTTTAAGGGA
ATAGACCTGGATGAGTTGAACATGGTGGCCTATGGGGACGATGTGCTGGCCAGTTACCCTTTTCCTATTGA
TTGCCTTGAATTGGCTAAGACTGGCAAAGAGTATGGTTTGACCATGACTCCTGCAGACAAATCACCCTGTT
TCAATGAAGTAACATGGGAGAATGCTACCTTCCTGAAGAGAGGGTTCTTGCCAGACCACCAATTTCCATTC
TTAATTCACCCTACGATGCCCATGAGAGAGATCCATGAGTCCATTCGATGGACTAAGGACGCGCGTAACAC
CCAGGATCACGTGCGCTCCCTGTGTCTATTGGCATGGCACAATGGTAAGGATGAATATGAAAAGTTTGTGA
GTGCAATTAGATCAGTTCCAGTTGGAAAAGCGTTGGCCATTCCTAACTTTGAGAATCTGAGAAGAAATTGG
CTCGAATTGTTTTAATATTACAGTTTAAAGCTGAACCCCACTAGAAATCTGGTCGTGTTAATGACTAGTGG
GGGTAAATTTGTTATAACCGGAATAGC

>EV-A71/Hap3
TTAAAACAGCTGTGGGTTGTACCCACTCACAGGGCCCACGTGGCGCTAGCACTCTGGTTCTGCGGAACCTT
TGTGCGCCTGTTTTACGCCCCCCCCCCAATTTGCAACTTAGAAGCAATACACAACACTGATCAACAGCAGG
CATGGCGCACCAGCTATGTCTTGATCAAGCACTTCTGTTTCCCCGGGCCGAGTATCAATAGACTGTTCACG
CGGTTGAAGGAGAAAGCGCCCGTTATCCGGCTAACTACTTCGAGAAACCTAGTAGCACCATTGAAGCTGCA
GAGTGCTTCGCTCGGCACTTCCCCCGTGTAGATCAGGTCGATGAGTCACTGCAATCCCCACGGGCGACCGT
GGCAGTGGCTGCGCTGGCGGCCTGCCTATGGGGCAACCCATAGGACGCTCTAATGTGGACATGGTGCGAAG
AGTCTATTGAGCTAGTTAGTAGTCCTCCGGCCCCTGAATGCGGCTAATCCTAACTGTGGAGCACATGCCTT
CAATCCAGAGGGTAGTGTGTCGTAATGGGCAACTCTGCAGCGGAACCGACTACTTTGGGTGTCCGTGTTTC
CTTTTATCTTTACATTGGCTGCTTATGGTGACGATTATAGAATTGTTACCATATAGCTATTGGATTGGCCA
TCCGGTGTGCAATAGAGCTATTATATACCTGTTTGTTGGCTTTGTACCACTAACCTTAAAATCTATAACCA
CCCTCGATTTTATATTAACCCTCAATACAATCAAACATGGGCTCACAGGTGTCTACTCAGCGATCCGGCTC
CCACGAGAACTCCAATTCAGCTACAGAAGGCTCCACCATTAATTACACTACCATCAACTATTACAAAGACT
CCTATGCTGCGACAGCGGGCAAACAGAGCCTCAAGCAAGACCCTGATAAATTTGCTAACCCTGTCAAGGAC
ATTTTCACTGAAATGGCTGCACCACTGAAGTCTCCATCCGCTGAGGCTTGTGGTTACAGTGATCGCGTGGC
ACAACTCACCATTGGAAACTCCACCATCACTACACAGGAGGCGGCGAATATCATAGTCGGTTATGGTGAGT
GGCCCTCATACTGCTCTGATGACGATGCTACAGCGGTGGACAAGCCAACGCGCCCAGATGTTTCAGTGAAT
AGGTTTTATACGTTGGATACTAAATTGTGGGAAAAGTCATCCAAGGGGTGGTATTGGAAGTTTCCTGATGT
ACTGACTGAGACCGGAGTCTTTGGCCAGAATGCACAGTTTCACTATTTATATAGGTCAGGATTTTGCATTC
ATGTGCAATGTAATGCTAGCAAGTTCCATCAAGGAGCGTTGTTAGTCGCCATACTTCCAGAGTATGTTATA
GGGACAGTGGCAGGCGGCACAGGAACTGAGGACAGCCACCCTCCTTACAAACAAACACAACCTGGCGCCGA
TGGTTTTGAGTTGCAGCACCCGTACGTACTCGATGCTGGGATTCCTATATCACAATTAACAGTGTGCCCCC
ACCAATGGATTAACCTACGGACCAATAACTGTGCCACAATAATAGTGCCGTATATGAACACACTGCCTTTC
GACTCTGCCCTGAACCATTGCAACTTTGGGCTGTTGGTGGTGCCCATTAGCCCACTAGATTTTGACCAAGG

GGCAACTCCGGTTATCCCTATTACAATCACTCTAGCTCCAATGTGCTCTGAGTTTGCAGGTCTCAGACAGG
CGGTCACTCAAGGTTTTCCCACCGAGCCAAAACCAGGAACGAATCAATTTTTGACCACCGATGACGGTGTC
TCAGCACCCATTTTACCAAATTTCCACCCCACACCATGTATTCACATACCCGGTGAAGTCAGAAACCTGCT
TGAGTTGTGTCAAGTGGAGACCATTCTTGAGGTTAACAATGTACCCACCAATGCCACCAGTCTGATGGAAA
GGCTACGATTCCCGGTGTCCGCGCAAGCGGGAAAAGGTGAATTGTGTGCCGTGTTTAGGGCCGACCCTGGA
AGAGACGGTCCATGGCAATCAACAATGCTGGGCCAGTTGTGTGGATATTACACCCAGTGGTCAGGATCACT
GGAGGTTACTTTTATGTTCACCGGGTCTTTCATGGCCACGGGTAAAATGCTCATAGCTTATACACCTCCTG
GTGGCCCCTTACCCAAAGATCGGGCCACAGCAATGCTGGGCACACATGTTATCTGGGATTTTGGGCTACAA
TCATCTGTCACCCTTGTAATACCATGGATTAGCAACACCCACTACAGAGCGCATGCCCGGGATGGAGTGTT
CGATTACTATACCACAGGACTGGTTAGTATCTGGTATCAAACAAACTACGTGGTTCCAATTGGGGCACCCA
ACACAGCTTACATAATAGCACTAGCGGCAGCCCAGAAGAATTTTACCATGAAACTGTGTAAAGACACCAGT
CACATATTACAGACAGCCTCTATTCAGGGAGATAGAGTGGCAGATGTGATAGAGAGCTCTATAGGAGATAG
TGTGAGTAGGGCACTTACCCAGGCCCTGCCAGCTCCAACAGGTCAGAACACGCAGGTGAGCAGTCATCGAC
TAGACACTGGTGAAGTTCCAGCGCTCCAAGCTGCTGAAATAGGGGCATCGTCAAATACTAGTGATGAGAGT
ATGATTGAGACACGATGCGTTCTTAATTCACACAGTACGGCAGAGACCACCCTGGACAGCTTCTTCAGTAG
GGCAGGCTTGGTAGGAGAGATAGATCTCCCTACTGAGGGTACCACTAATCCAAATGGTTATGCTAATTGGG
ATATAGACATAACTGGTTACGCACAAATGCGCAGGAAAGTGGAGCTGTTCACCTACATGCGCTTTGATGCG
GAATTCACTTTTGTTGCGTGCACTCCTACTGGTCAGGTTGTCCCACAATTACTTCAGTATATGTTTGTTCC
CCCTGGTGCTCCCAAACCAGAGTCTAGAGAATCACTTGCTTGGCAGACAGCCACAAACCCCTCAGTTTTTG
TCAAGTTGACTGATCCCCCGGCACAGGTCTCAGTTCCGTTCATGTCACCCGCGAGCGCTTACCAGTGGTTT
TACGACGGGTACCCCACGTTTGGAGAACACAAACAGGAGAAAGACCTTGAGTATGGAGCGTGCCCTAATAA
TATGATGGGCACTTTCTCGGTGCGAACTGTGGGTTCATCAAAGTCCAAGTATTCTTTGGTTGTCAGGATAT
ATATGAGAATGAAGCATGTCAGGGCGTGGATACCTCGCCCGATGCGCAACCAAAACTACCTGTTTAAAGCC
AATCCAAACTATGCCGGTAACTCCATCAAACCGACCGGCACTAGTCGTGCTGCCATTACTACCCTTGGAAA
GTTCGGCCAGCAATCTGGGGCCATCTACGTGGGCAACTTCAGAGTGGTTAATCGTCACCTCGCTACTCATA
ATGACTGGGCGAACCTCGTCTGGGAAGATAGCTCCCGCGACCTATTAGTGTCGTCTACCACCGCCCAGGGC
TGTGATACAATTGCACGTTGTGACTGTCAAACAGGAGTGTACTATTGTAATTCCAAAAGAAAGCACTATCC
AGTCAGCTTCTCCAAACCCAGCCTCATATATGTGGAGGCTAGCGAGTATTACCCTGCTAGATACCAATCGC
ACCTGATGCTTGCAGCAGGCCACTCTGAGCCCGGCGACTGCGGGGGCATCTTAAGGTGTCAACATGGTGTA
GTTGGTATAGTGTCCACGGGTGGCAACGGGCTCGTTGGTTTTGCTGATGTGAGGGATCTCTTGTGGTTGGA
TGAAGAGGCCATGGAGCAAGGTGTGTCTGACTACATTAAGGGGCTCGGTGACGCATTTGGAACAGGTTTCA
CTGATGCTGTATCCAGGGAAGTTGAAGCCCTCAGGAACCACCTCATAGGATCTGATGGAGCAGTGGAAAAA
ATCCTAAAGAACCTTATTAAGCTGATTTCAGCGTTAGTAATTGTGATTAGGAGCGATTATGATATGGTCAC
CCTCACAGCAACTTTAGCCCTGATTGGTTGTCATGGAAGTCCCTGGGCTTGGATTAAAGCCAAAACAGCAT
CCATTTTAGGTATCCCCATCGCCCAGAAGCAGAGCGCTTCTTGGCTAAAGAAATTTAATGATATGGCGAGT
GCTGCCAAGGGTTTAGAATGGATATCCAACAAAATTAGTAAGTTCATTGACTGGCTCAGGGAGAAGATTGT
TCCAGCAGCTAAAGAGAAAGCAGAATTTTTAACCAATTTGAAGCAATTACCACTATTAGAGAACCAGATCA
CGAACTTGGAGCAGTCCGCTGCCTCGCAAGAGGACCTTGAAGCTATGTTTGGGAATGTGTCATACCTCGCC
CATTTCTGTCGCAAGTTCCAACCATTATACGCCACGGAGGCCAAGCGAGTCTATGTTCTAGAGAAGAGAAT
GAACAATTACATGCAGTTCAAGAGCAAACACCGTATTGAACCTGTATGTCTCATCATTAGAGGCTCACCAG
GCACTGGAAAGTCCCTTGCGACCGGCATCATTGCCCGGGCCATAGCAGACAAGTACCACTCTAGTGTGTAC
TCACTCCCACCGGATCCTGACCATTTTGACGGGTACAAACAGCAAGTGGTTACAGTTATGGATGACCTGTG
CCAGAATCCTGACGGCAAAGACATGTCATTATTTTGCCAGATGGTATCCACCGTGGATTTTATTCCACCAA
TGGCTTCTCTCGAAGAAAAGGGAGTTTCTTTCACATCTAAATTTGTTATCGCATCCACCAACGCCAGCAAC
ATTATAGTGCCCACAGTGTCTGACTCTGACGCCATTCGTCGCAGGTTCTACATGGATTGCGACATTGAGGT
CACAGACTCATACAAAACAGACTTGGGTAGACTAGACGCTGGGCGGGCTGCTAAGTTATGCTCTGAAAACA
ACACCGCAAATTTCAAACGATGCAGCCCACTAGTGTGTGGGAAAGCTATTCAACTTAGAGACAGGAAATCC
AAGGTCAGGTATAGCGTGGACACAGTGGTCTCTGAACTTATTAGAGAATACAATAGCAGATCCGCTATTGG
TAACACAATTGAAGCATTATTCCAAGGCCCACCCAAGTTCAGGCCAATAAGGATCAGTCTTGAGGAGAAGC
CAGCCCCAGACGCTATTAGCGATCTCCTTGCTAGTGTGGATAGCGAGGAAGTGCGCCAATACTGTAGGGAA
CAAGGCTGGATTATCCCTGAAACTCCCACCAATGTTGAACGACATCTTAATAGAGCAGTGCTAGTCGTGCA
ATCCATCACTACTGTGGTGGCAGTCGTCTCACTGGTGTACGTCATTTACAAGCTCTTTGCGGGGTTTCAAG
GTGCGTATTCTGGAGCTCCCAAGCAAGTGCTCAAGAAGCCTGTCCTCCGCACGGCAACAGTGCAGGGTCCA
AGCCTTGATTTTGCCCTATCCTTGCTGAGGAGGAACATCAGGCAAGTCCAAACAGACCAAGGGCATTTTAC
CATGTTGGGTGTCAGGGATCGCCTGGCTGTTCTCCCGCGGCACTCACAGCCCGGGAAGACTATTTGGGTGG
AACACAAACTTGTGAACATCCTTGATGCAGTCGAGCTGGTGGACGAGCAGGGCGTTAATTTGGAACTCACA
TTGGTGACACTAGATATTAATGAAAAATTTAGAGATATCACCAAGTTCATTCCAGAGACCATTAGCGGCGC
TAGTGATGCAACTCTAGTGATCAACACAGAACATATGCCGTCAATGTTTGTCCCTGTGGGGGACGTCGTGC

AGTACGGGTTCTTGAACCTCAGTGGAAAGCCAACACATAGGACCATGATGTACAATTTCCCTACAAAAGCA
GGACAGTGTGGAGGCGTGGTTACATCAGTCGGTAAGATTGTTGGTATTCACATTGGTGGCAACGGGCGCCA
AGGGTTCTGCGCTGGTTTGAAGAGGAGCTACTTTGCGAGTATGCAAGGTGAGATCCAATGGGTGAAGCCTA
ACAAGGAAACTGGCAGACTAAACATCAATGGACCAACTCGCACTAAGTTGGAGCCTAGTGTATTTCATGAT
GTGTTTGAAGGCAACAAGGAACCAGCAGTTTTAACAAGTAAAGACCCTAGATTGGAGGTCGACTTTGAACA
AGCCCTGTTTTCCAAGTATGTGGGCAATGTTTTACACGAGCCCGATGAATATGTGACTCAAGCTGCCCTCC
ACTATGCGAATCAACTTAAACAATTGGACATAAACACTAGCAAGATGAGCATGGAGGAAGCGTGCTATGGC
ACTGAAAACCTGGAAGCAATAGACCTCTGCACTAGTGCTGGGTATCCATACAGTGCCCTTGGTATCAAGAA
AAGAGACATTCTCGACCCCATAACCAGGGATGTGTCTAAGATGAAATTCTACATGGATAAATACGGACTAG
ATCTGCCATACTCTACCTATGTGAAGGATGAACTTAGATCTCTGGATAAAATCAAGAAAGGAAAGTCACGC
CTGATAGAGGCCAGCAGCTTGAATGACTCTGTCTACCTCAGAATGACTTTTGGGCACCTTTACGAGGTGTT
TCATGCTAACCCTGGTACTGTGACTGGCTCAGCAGTAGGTTGCAACCCAGACGTGTTTTGGAGTAAACTAC
CGATTCTGCTGCCTGGGTCACTCTTTGCCTTTGACTACTCAGGATATGATGCTAGTCTCAGCCCGGTATGG
TTCAGGGCTCTAGAAGTTGTGTTACGGGAGATTGGGTATTCAGAGGAGGCCGTGTCCCTAATAGAAGGAAT
CAACCACACCCACCATGTGTACCGGAATAAAACATACTGTGTACTTGGTGGGATGCCCTCAGGGTGCTCTG
GTACTTCCATCTTCAATTCAATGATCAACAACATCATCATTAGAACCCTTTTGATCAAAACCTTTAAGGGA
ATAGACCTGGATGAGTTGAACATGGTGGCCTATGGGGACGATGTGCTGGCCAGTTACCCTTTTCCTATTGA
TTGCCTTGAATTGGCTAAGACTGGCAAAGAGTATGGTTTGACCATGACTCCTGCAGACAAATCACCCTGTT
TCAATGAAGTAACATGGGAGAATGCTACCTTCCTGAAGAGAGGGTTCTTGCCAGACCACCAATTTCCATTC
TTAATTCACCCTACGATGCCCATGAGAGAGATCCATGAGTCCATTCGATGGACTAAGGACGCGCGTAACAC
CCAGGATCACGTGCGCTCCCTGTGTCTATTGGCATGGCACAATGGTAAGGATGAATATGAAAAGTTTGTGA
GTGCAATTAGATCAGTTCCAGTTGGAAAAGCGTTGGCCATTCCTAACTTTGAGAATCTGAGAAGAAATTGG
CTCGAATTGTTTTAATATTACAGTTTAAAGCTGAACCCCACTAGAAATCTGGTCGTGTTAATGACTAGTGG
GGGTAAATTTGTTATAACCGGAATAGC

>EV-A71/Hap4
TTAAAACAGCTGTGGGTTGTACCCACTCACAGGGCCCACGTGGCGCTAGCACTCTGGTTCTGCGGAACCTT
TGTGCGCCTGTTTTACGCCCCCCCCCCAATTTGCAACTTAGAAGCAATACACAACACTGATCAACAGCAGG
CATGGCGCACCAGCTATGTCTTGATCAAGCACTTCTGTTTCCCCGGGCCGAGTATCAATAGACTGTTCACG
CGGTTGAAGGAGAAAGCGCCCGTTATCCGGCTAACTACTTCGAGAAACCTAGTAGCACCATTGAAGCTGCA
GAGTGTTTCGCTCGGCACTTCCCCCGTGTAGATCAGGTCGATGAGTCACTGCAATCCCCACGGGCGACCGT
GGCAGTGGCTGCGCTGGCGGCCTGCCTATGGGGCAACCCATAGGACGCTCTAATGTGGACATGGTGCGAAG
AGTCTATTGAGCTAGTTAGTAGTCCTCCGGCCCCTGAATGCGGCTAATCCTAACTGTGGAGCACATGCCTT
CAATCCAGAGGGTAGTGTGTCGTAATGGGCAACTCTGCAGCGGAACCGACTACTTTGGGTGTCCGTGTTTC
CTTTTATCTTTACATTGGCTGCTTATGGTGACGATTATAGAATTGTTACCATATAGCTATTGGATTGGCCA
TCCGGTGTGCAATAGAGCTATTATATACCTGTTTGTTGGCTTTGTACCACTAACCTTAAAATCTATAACCA
CCCTCGATTTTATATTAACCCTCAATACAATCAAACATGGGCTCACAGGTGTCTACTCAGCGATCCGGCTC
CCACGAGAACTCCAATTCGGCTACAGAAGGCTCCACCATTAATTACACTACCATCAACTATTACAAAGACT
CCTATGCTGCGACAGCGGGCAAACAGAGCCTCAAGCAAGACCCTGATAAATTTGCTAACCCTGTCAAGGAC
ATTTTCACTGAAATGGCTGCACCACTGAAGTCTCCATCCGCTGAGGCTTGTGGTTACAGTGATCGCGTGGC
ACAACTCACCATTGGAAACTCCACCATCACTACACAGGAGGCGGCGAATATCATAGTCGGTTATGGTGAGT
GGCCCTCATACTGCTCTGATGACGATGCTACAGCGGTGGACAAGCCAACGCGCCCAGATGTTTCAGTGAAT
AGGTTTTATACGTTGGATACTAAATTGTGGGAAAAGTCATCCAAGGGGTGGTATTGGAAGTTTCCTGATGT
ACTGACTGAGACCGGAGTCTTTGGCCAGAATGCACAGTTTCACTATTTATATAGGTCAGGATTTTGCATTC
ATGTGCAATGTAATGCTAGCAAGTTCCATCAAGGAGCGTTGTTAGTCGCCATACTTCCAGAGTATGTTATA
GGGACAGTGGCAGGCGGCACAGGAACTGAGGACAGCCACCCTCCTTACAAACAAACACAACCTGGCGCCGA
TGGTTTTGAGTTGCAGCACCCGTACGTACTCGATGCTGGGATTCCTATATCACAATTAACAGTGTGCCCCC
ACCAATGGATTAACCTACGGACCAATAACTGTGCCACAATAATAGTGCCGTATATGAACACACTGCCTTTC
GACTCTGCCCTGAACCATTGCAACTTTGGGCTGTTGGTGGTGCCCATTAGCCCACTAGATTTTGACCAAGG
GGCAACTCCGGTTATCCCTATTACAATCACTCTAGCTCCAATGTGCTCTGAGTTTGCAGGTCTCAGACAGG
CGGTCACTCAAGGTTTTCCCACCGAGCCAAAACCAGGAACGAATCAATTTTTGACCACCGATGACGGTGTC
TCAGCACCCATTTTACCAAATTTCCACCCCACACCATGTATTCACATACCCGGTGAAGTCAGAAACCTGCT
TGAGTTGTGTCAAGTGGAGACCATTCTTGAGGTTAACAATGTACCCACCAATGCCACCAGTCTGATGGAAA
GGCTACGATTCCCGGTGTCCGCGCAAGCGGGAAAAGGTGAATTGTGTGCCGTGTTTAGGGCCGACCCTGGA
AGAGACGGTCCATGGCAATCAACAATGCTGGGCCAGTTGTGTGGATATTACACCCAGTGGTCAGGATCACT
GGAGGTTACTTTTATGTTCACCGGGTCTTTCATGGCCACGGGTAAAATGCTCATAGCTTATACACCTCCTG
GTGGCCCCTTACCCAAAGATCGGGCCACAGCAATGCTGGGCACACATGTTATCTGGGATTTTGGGCTACAA
TCATCTGTCACCCTTGTAATACCATGGATTAGCAACACCCACTACAGAGCGCATGCCCGGGATGGAGTGTT

CGATTACTATACCACAGGACTGGTTAGTATCTGGTATCAAACAAACTACGTGGTTCCAATTGGGGCACCCA
ACACAGCTTACATAATAGCACTAGCGGCAGCCCAGAAGAATTTTACCATGAAACTGTGTAAAGACACCAGT
CACATATTACAGACAGCCTCTATTCAGGGAGATAGAGTGGCAGATGTGATAGAGAGCTCTATAGGAGATAG
TGTGAGTAGGGCACTTACCCAGGCCCTGCCAGCTCCAACAGGTCAGAACACGCAGGTGAGCAGTCATCGAC
TAGACACTGGTGAAGTTCCAGCGCTCCAAGCTGCTGAAATAGGGGCATCGTCAAATACTAGTGATGAGAGT
ATGATTGAGACACGATGCGTTCTTAATTCACACAGTACGGCAGAGACCACCCTGGACAGCTTCTTCAGTAG
GGCAGGCTTGGTAGGAGAGATAGATCTCCCTATTGAGGGTACCACTAATCCAAATGGTTATGCTAATTGGG
ATATAGACATAACTGGTTACGCACAAATGCGCAGGAAAGTGGAGCTGTTCACCTACATGCGCTTTGATGCG
GAATTCACTTTTGTTGCGTGCACTCCTACTGGTCAGGTTGTCCCACAATTACTTCAGTATATGTTTGTTCC
CCCTGGTGCTCCCAAACCAGAGTCTAGAGAATCACTTGCTTGGCAGACAGCCACAAACCCCTCAGTTTTTG
TCAAGTTGACTGATCCCCCGGCACAGGTCTCAGTTCCGTTCATGTCACCCGCGAGCGCTTACCAGTGGTTT
TACGACGGGTACCCCACGTTTGGAGAACACAAACAGGAGAAAGACCTTGAGTATGGAGCGTGCCCTAATAA
TATGATGGGCACTTTCTCGGTGCGAACTGTGGGTTCATCAAAGTCCAAGTATTCTTTGGTTGTCAGGATAT
ATATGAGAATGAAGCATGTCAGGGCGTGGATACCTCGCCCGATGCGCAACCAAAACTACCTGTTTAAAGCC
AATCCAAACTATGCCGGTAACTCCATCAAACCGACCGGCACTAGTCGTACTGCCATTACTACCCTTGGAAA
GTTCGGCCAGCAATCTGGGGCCATCTACGTGGGCAACTTCAGAGTGGTTAATCGTCACCTCGCTACTCATA
ATGACTGGGCGAACCTCGTCTGGGAAGATAGCTCCCGCGACCTATTAGTGTCGTCTACCACCGCCCAGGGC
TGTGATACAATTGCACGTTGTGACTGTCAAACAGGAGTGTACTATTGTAATTCCAAAAGAAAGTACTATCC
AGTCAGCTTCTCCAAACCCAGCCTCATATATGTGGAGGCTAGCGAGTATTACCCTGCTAGATACCAATCGC
ACCTGATGCTTGCAGCAGGCCACTCTGAGCCCGGCGACTGCGGGGGCATCTTAAGGTGTCAACATGGTGTA
GTTGGTATAGTGTCCACGGGTGGCAACGGGCTCGTTGGTTTTGCTGATGTGAGGGATCTCTTGTGGTTGGA
TGAAGAGGCCATGGAGCAAGGTGTGTCTGACTACATTAAGGGGCTCGGTGACGCATTTGGAACAGGTTTCA
CTGATGCTGTATCCAGGGAAGTTGAAGCCCTCAGGAACCACCTCATAGGATCTGATGGAGCAGTGGAAAAA
ATCCTAAAGAACCTTATTAAGCTGATTTCAGCGTTAGTAATTGTGATTAGGAGCGATTATGATATGGTCAC
CCTCACAGCAACTTTAGCCCTGATTGGTTGTCATGGAAGTCCCTGGGCTTGGATTAAAGCCAAAACAGCAT
CCATTTTAGGTATCCCCATCGCCCAGAAGCAGAGCGCTTCTTGGCTAAAGAAATTTAATGATATGGCGAGT
GCTGCCAAGGGTTTAGAATGGATATCCAACAAAATTAGTAAGTTCATTGACTGGCTCAGGGAGAAGATTGT
TCCAGCAGCTAAAGAGAAAGCAGAATTTTTAACCAATTTGAAGCAATTACCACTATTAGAGAACCAGATCA
CGAACTTGGAGCAGTCCGCTGCCTCGCAAGAGGACCTTGAAGCTATGTTTGGGAATGTGTCATACCTCGCC
CATTTCTGTCGCAAGTTCCAACCATTATACGCCACGGAGGCCAAGCGAGTCTATGTTCTAGAGAAGAGAAT
GAACAATTACATGCAGTTCAAGAGCAAACACCGTATTGAACCTGTATGTCTCATCATTAGAGGCTCACCAG
GCACTGGAAAGTCCCTTGCGACCGGCATCATTGCCCGGGCCATAGCAGACAAGTACCACTCTAGTGTGTAC
TCACTCCCACCGGATCCTGACCATTTTGACGGGTACAAACAGCAAGTGGTTACAGTTATGGATGACCTGTG
CCAGAATCCTGACGGCAAAGACATGTCATTATTTTGCCAGATGGTATCCACCGTGGATTTTATTCCACCAA
TGGCTTCTCTCGAAGAAAAGGGAGTTTCTTTCACATCTAAATTTGTTATCGCATCCACCAACGCCAGCAAC
ATTATAGTGCCCACAGTGTCTGACTCTGACGCCATTCGTCGCAGGTTCTACATGGATTGCGACATTGAGGT
CACAGACTCATACAAAACAGACTTGGGTAGACTAGACGCTGGGCGGGCTGCTAAGTTATGCTCTGAAAACA
ACACCGCAAATTTCAAACGATGCAGCCCACTAGTGTGTGGGAAAGCTATTCAACTTAGAGACAGGAAATCC
AAGGTCAGGTATAGCGTGGACACAGTGGTCTCTGAACTTATTAGAGAATACAATAGCAGATCCGCTATTGG
TAACACAATTGAAGCATTATTCCAAGGCCCACCCAAGTTCAGGCCAATAAGGATCAGTCTTGAGGAGAAGC
CAGCCCCAGACGCTATTAGCGATCTCCTTGCTAGTGTGGATAGCGAGGAAGTGCGCCAATACTGTAGGGAA
CAAGGCTGGATTATCCCTGAAACTCCCACCAATGTTGAACGACATCTTAATAGAGCAGTGCTAGTCGTGCA
ATCCATCACTACTGTGGTGGCAGTCGTCTCACTGGTGTACGTCATTTACAAGCTCTTTGCGGGGTTTCAAG
GTGCGTATTCTGGAGCTCCCAAGCAAGTGCTCAAGAAGCCTGTCCTCCGCACGGCAACAGTGCAGGGTCCA
AGCCTTGATTTTGCCCTATCCTTGCTGAGGAGGAACATCAGGCAAGTCCAAACAGACCAAGGGCATTTTAC
CATGTTGGGTGTCAGGGATCGCCTGGCTGTTCTCCCGCGGCACTCACAGCCCGGGAAGACTATTTGGGTGG
AACACAAACTTGTGAACATCCTTGATGCAGTCGAGCTGGTGGACGAGCAGGGCGTTAATTTGGAACTCACA
TTGGTGACACTAGATATTAATGAAAAATTTAGAGATATCACCAAGTTCATTCCAGAGACCATTAGCGGCGC
TAGTGATGCAACTCTAGTGATCAACACAGAACATATGCCGTCAATGTTTGTCCCTGTGGGGGACGTCGTGC
AGTACGGGTTCTTGAACCTCAGTGGAAAGCCAACACATAGGACCATGATGTACAATTTCCCTACAAAAGCA
GGACAGTGTGGAGGCGTGGTTACATCAGTCGGTAAGATTGTTGGTATTCACATTGGTGGCAACGGGCGCCA
AGGGTTCTGCGCTGGTTTGAAGAGGAGCTACTTTGCGAGTATGCAAGGTGAGATCCAATGGGTGAAGCCTA
ACAAGGAAACTGGCAGACTAAACATCAATGGACCAACTCGCACTAAGTTGGAGCCTAGTGTATTTCATGAT
GTGTTTGAAGGCAACAAGGAACCAGCAGTTTTAACAAGTAAAGACCCTAGATTGGAGGTCGACTTTGAACA
AGCCCTGTTTTCCAAGTATGTGGGCAATGTTTTACACGAGCCCGATGAATATGTGACTCAAGCTGCCCTCC
ACTATGCGAATCAACTTAAACAATTGGACATAAACACTAGCAAGATGAGCATGGAGGAAGCGTGCTATGGC
ACTGAAAACCTGGAAGCAATAGACCTCTGCACTAGTGCTGGGTATCCATACAGTGCCCTTGGTATCAAGAA
AAGAGACATTCTCGACCCCATAACCAGGGATGTGTCTAAGATGAAATTCTACATGGATAAATACGGACTAG

ATCTGCCATACTCTACCTATGTGAAGGATGAACTTAGATCTCTGGATAAAATCAAGAAAGGAAAGTCACGC
CTGATAGAGGCCAGCAGCTTGAATGACTCTGTCTACCTCAGAATGACTTTTGGGCACCTTTACGAGGTGTT
TCATGCTAACCCTGGTACTGTGACTGGCTCAGCAGTAGGTTGCAACCCAGACGTGTTTTGGAGTAAACTAC
CGATTCTGCTGCCTGGGTCACTCTTTGCCTTTGACTACTCAGGATATGATGCTAGTCTCAGCCCGGTATGG
TTCAGGGCTCTAGAAGTTGTGTTACGGGAGATTGGGTATTCAGAGGAGGCCGTGTCCCTAATAGAAGGAAT
CAACCACACCCACCATGTGTACCGGAATAAAACATACTGTGTACTTGGTGGGATGCCCTCAGGGTGCTCTG
GTACTTCCATCTTCAATTCAATGATCAACAACATCATCATTAGAACCCTTTTGATCAAAACCTTTAAGGGA
ATAGACCTGGATGAGTTGAACATGGTGGCCTATGGGGACGATGTGCTGGCCAGTTACCCTTTTCCTATTGA
TTGCCTTGAATTGGCTAAGACTGGCAAAGAGTATGGTTTGACCATGACTCCTGCAGACAAATCACCCTGTT
TCAATGAAGTAACATGGGAGAATGCTACCTTCCTGAAGAGAGGGTTCTTGCCAGACCACCAATTTCCATTC
TTAATTCACCCTACGATGCCCATGAGAGAGATCCATGAGTCCATTCGATGGACTAAGGACGCGCGTAACAC
CCAGGATCACGTGCGCTCCCTGTGTCTATTGGCATGGCACAATGGTAAGGATGAATATGAAAAGTTTGTGA
GTGCAATTAGATCAGTTCCAGTTGGAAAAGCGTTGGCCATTCCTAACTTTGAGAATCTGAGAAGAAATTGG
CTCGAATTGTTTTAATATTACAGTTTAAAGCTGAACCCCACTAGAAATCTGGTCGTGTTAATGACTAGTGG
GGGTAAATTTGTTATAACCGGAATAGC

>EV-A71/Hap5
TTAAAACAGCTGTGGGTTGTACCCACTCACAGGGCCCACGTGGCGCTAGCACTCTGGTTCTGCGGAACCTT
TGTGCGCCTGTTTTACGCCCCCCCCCCAATTTGCAACTTAGAAGCAATACACAACACTGATCAACAGCAGG
CATGGCGCACCAGCTATGTCTTGATCAAGCACTTCTGTTTCCCCGGGCCGAGTATCAATAGACTGTTCACG
CGGTTGAAGGAGAAAGCGCCCGTTATCCGGCTAACTACTTCGAGAAACCTAGTAGCACCATTGAAGCTGCA
GAGTGTTTCGCTCGGCACTTCCCCCGTGTAGATCAGGTCGATGAGTCACTGCAATCCCCACGGGCGACCGT
GGCAGTGGCTGCGCTGGCGGCCTGCCTATGGGGCAACCCATAGGACGCTCTAATGTGGACATGGTGCGAAG
AGTCTATTGAGCTAGTTAGTAGTCCTCCGGCCCCTGAATGCGGCTAATCCTAACTGTGGAGCACATGCCTT
CAATCCAGAGGGTAGTGTGTCGTAATGGGCAACTCTGCAGCGGAACCGACTACTTTGGGTGTCCGTGTTTC
CTTTTATCTTTACATTGGCTGCTTATGGTGACGATTATAGAATTGTTACCATATAGCTATTGGATTGGCCA
TCCGGTGTGCAATAGAGCTATTATATACCTGTTTGTTGGCTTTGTACCACTAACCTTAAAATCTATAACCA
CCCTCGATTTTATATTAACCCTCAATACAATCAAACATGGGCTCACAGGTGTCTACTCAGCGATCCGGCTC
CCACGAGAACTCCAATTCGGCTACAGAAGGCTCCACCATTAATTACACTACCATCAACTATTACAAAGACT
CCTATGCTGCGACAGCGGGCAAACAGAGCCTCAAGCAAGACCCTGATAAATTTGCTAACCCTGTCAAGGAC
ATTTTCACTGAAATGGCTGCACCACTGAAGTCTCCATCCGCTGAGGCTTGTGGTTACAGTGATCGCGTGGC
ACAACTCACCATTGGAAACTCCACCATCACTACACAGGAGGCGGCGAATATCATAGTCGGTTATGGTGAGT
GGCCCTCATACTGCTCTGATGACGATGCTACAGCGGTGGACAAGCCAACGCGCCCAGATGTTTCAGTGAAT
AGGTTTTATACGTTGGATACTAAATTGTGGGAAAAGTCATCCAAGGGGTGGTATTGGAAGTTTCCTGATGT
ACTGACTGAGACCGGAGTCTTTGGCCAGAATGCACAGTTTCACTATTTATATAGGTCAGGATTTTGCATTC
ATGTGCAATGTAATGCTAGCAAGTTCCATCAAGGAGCGTTGTTAGTCGCCATACTTCCAGAGTATGTTATA
GGGACAGTGGCAGGCGGCACAGGAACTGAGGACAGCCACCCTCCTTACAAACAAACACAACCTGGCGCCGA
TGGTTTTGAGTTGCAGCACCCGTACGTACTCGATGCTGGGATTCCTATATCACAATTAACAGTGTGCCCCC
ACCAATGGATTAACCTACGGACCAATAACTGTGCCACAATAATAGTGCCGTATATGAACACACTGCCTTTC
GACTCTGCCCTGAACCATTGCAACTTTGGGCTGTTGGTGGTGCCCATTAGCCCACTAGATTTTGACCAAGG
GGCAACTCCGGTTATCCCTATTACAATCACTCTAGCTCCAATGTGCTCTGAGTTTGCAGGTCTCAGACAGG
CGGTCACTCAAGGTTTTCCCACCGAGCCAAAACCAGGAACGAATCAATTTTTGACCACCGATGACGGTGTC
TCAGCACCCATTTTACCAAATTTCCACCCCACACCATGTATTCACATACCCGGTGAAGTCAGAAACCTGCT
TGAGTTGTGTCAAGTGGAGACCATTCTTGAGGTTAACAATGTACCCACCAATGCCACCAGTCTGATGGAAA
GGCTACGATTCCCGGTGTCCGCGCAAGCGGGAAAAGGTGAATTGTGTGCCGTGTTTAGGGCCGACCCTGGA
AGAGACGGTCCATGGCAATCAACAATGCTGGGCCAGTTGTGTGGATATTACACCCAGTGGTCAGGATCACT
GGAGGTTACTTTTATGTTCACCGGGTCTTTCATGGCCACGGGTAAAATGCTCATAGCTTATACACCTCCTG
GTGGCCCCTTACCCAAAGATCGGGCCACAGCAATGCTGGGCACACATGTTATCTGGGATTTTGGGCTACAA
TCATCTGTCACCCTTGTAATACCATGGATTAGCAACACCCACTACAGAGCGCATGCCCGGGATGGAGTGTT
CGATTACTATACCACAGGACTGGTTAGTATCTGGTATCAAACAAACTACGTGGTTCCAATTGGGGCACCCA
ACACAGCTTACATAATAGCACTAGCGGCAGCCCAGAAGAATTTTACCATGAAACTGTGTAAAGACACCAGT
CACATATTACAGACAGCCTCTATTCAGGGAGATAGAGTGGCAGATGTGATAGAGAGCTCTATAGGAGATAG
TGTGAGTAGGGCACTTACCCAGGCCCTGCCAGCTCCAACAGGTCAGAACACGCAGGTGAGCAGTCATCGAC
TAGACACTGGTGAAGTTCCAGCGCTCCAAGCTGCTGAAATAGGGGCATCGTCAAATACTAGTGATGAGAGT
ATGATTGAGACACGATGCGTTCTTAATTCACACAGTACGGCAGAGACCACCCTGGACAGCTTCTTCAGTAG
GGCAGGCTTGGTAGGAGAGATAGATCTCCCTACTGAGGGTACCACTAATCCAAATGGTTATGCTAATTGGG
ATATAGACATAACTGGTTACGCACAAATGCGCAGGAAAGTGGAGCTGTTCACCTACATGCGCTTTGATGCG
GAATTCACTTTTGTTGCGTGCACTCCTACTGGTCAGGTTGTCCCACAATTACTTCAGTATATGTTTGTTCC

CCCTGGTGCTCCCAAACCAGAGTCTAGAGAATCACTTGCTTGGCAGACAGCCACAAACCCCTCAGTTTTTG
TCAAGTTGACTGATCCCCCGGCACAGGTCTCAGTTCCGTTCATGTCACCCGCGAGCGCTTACCAGTGGTTT
TACGACGGGTACCCCACGTTTGGAGAACACAAACAGGAGAAAGACCTTGAGTATGGAGCGTGCCCTAATAA
TATGATGGGCACTTTCTCGGTGCGAACTGTGGGTTCATCAAAGTCCAAGTATTCTTTGGTTGTCAGGATAT
ATATGAGAATGAAGCATGTCAGGGCGTGGATACCTCGCCCGATGCGCAACCAAAACTACCTGTTTAAAGCC
AATCCAAACTATGCCGGTAACTCCATCAAACCGACCGGCACTAGTCGTGCTGCCATTACTACCCTTGGAAA
GTTCGGCCAGCAATCTGGGGCCATCTACGTGGGCAACTTCAGAGTGGTTAATCGTCACCTCGCTACTCATA
ATGACTGGGCGAACCTCGTCTGGGAAGATAGCTCCCGCGACCTATTAGTGTCGTCTACCACCGCCCAGGGC
TGTGATACAATTGCACGTTGTGACTGTCAAACAGGAGTGTACTATTGTAATTCCAAAAGAAAGCACTATCC
AGTCAGCTTCTCCAAACCCAGCCTCATATATGTGGAGGCTAGCGAGTATTACCCTGCTAGATACCAATCGC
ACCTGATGCTTGCAGCAGGCCACTCTGAGCCCGGCGACTGCGGGGGCATCTTAAGGTGTCAACATGGTGTA
GTTGGTATAGTGTCCACGGGTGGCAACGGGCTCGTTGGTTTTGCTGATGTGAGGGATCTCTTGTGGTTGGA
TGAAGAGGCCATGGAGCAAGGTGTGTCTGACTACATTAAGGGGCTCGGTGACGCATTTGGAACAGGTTTCA
CTGATGCTGTATCCAGGGAAGTTGAAGCCCTCAGGAACCACCTCATAGGATCTGATGGAGCAGTGGAAAAA
ATCCTAAAGAACCTTATTAAGCTGATTTCAGCGTTAGTAATTGTGATTAGGAGCGATTATGATATGGTCAC
CCTCACAGCAACTTTAGCCCTGATTGGTTGTCATGGAAGTCCCTGGGCTTGGATTAAAGCCAAAACAGCAT
CCATTTTAGGTATCCCCATCGCCCAGAAGCAGAGCGCTTCTTGGCTAAAGAAATTTAATGATATGGCGAGT
GCTGCCAAGGGTTTAGAATGGATATCCAACAAAATTAGTAAGTTCATTGACTGGCTCAGGGAGAAGATTGT
TCCAGCAGCTAAAGAGAAAGCAGAATTTTTAACCAATTTGAAGCAATTACCACTATTAGAGAACCAGATCA
CGAACTTGGAGCAGTCCGCTGCCTCGCAAGAGGACCTTGAAGCTATGTTTGGGAATGTGTCATACCTCGCC
CATTTCTGTCGCAAGTTCCAACCATTATACGCCACGGAGGCCAAGCGAGTCTATGTTCTAGAGAAGAGAAT
GAACAATTACATGCAGTTCAAGAGCAAACACCGTATTGAACCTGTATGTCTCATCATTAGAGGCTCACCAG
GCACTGGAAAGTCCCTTGCGACCGGCATCATTGCCCGGGCCATAGCAGACAAGTACCACTCTAGTGTGTAC
TCACTCCCACCGGATCCTGACCATTTTGACGGGTACAAACAGCAAGTGGTTACAGTTATGGATGACCTGTG
CCAGAATCCTGACGGCAAAGACATGTCATTATTTTGCCAGATGGTATCCACCGTGGATTTTATTCCACCAA
TGGCTTCTCTCGAAGAAAAGGGAGTTTCTTTCACATCTAAATTTGTTATCGCATCCACCAACGCCAGCAAC
ATTATAGTGCCCACAGTGTCTGACTCTGACGCCATTCGTCGCAGGTTCTACATGGATTGCGACATTGAGGTCAC
AGACTCATACAAAACAGACTTGGGTAGACTAGACGCTGGGCGGGCTGCTAAGTTATGCTCTGAAAACAACACCGC
AAATTTCAAACGATGCAGCCCACTAGTGTGTGGGAAAGCTATTCAACTTAGAGACAGGAAATCCAAGGTCAGGTA
TAGCGTGGACACAGTGGTCTCTGAACTTATTAGAGAATACAATAGCAGATCCGCTATTGGTAACACAATTGAAGC
ATTATTCCAAGGCCCACCCAAGTTCAGGCCAATAAGGATCAGTCTTGAGGAGAAGCCAGCCCCAGACGCTATTAG
CGATCTCCTTGCTAGTGTGGATAGCGAGGAAGTGCGCCAATACTGTAGGGAACAAGGCTGGATTATCCCTGAAAC
TCCCACCAATGTTGAACGACATCTTAATAGAGCAGTGCTAGTCGTGCAATCCATCACTACTGTGGTGGCAGTCGT
CTCACTGGTGTACGTCATTTACAAGCTCTTTGCGGGGTTTCAAGGTGCGTATTCTGGAGCTCCCAAGCAAGTGCT
CAAGAAGCCTGTCCTCCGCACGGCAACAGTGCAGGGTCCAAGCCTTGATTTTGCCCTATCCTTGCTGAGGAGGAA
CATCAGGCAAGTCCAAACAGACCAAGGGCATTTTACCATGTTGGGTGTCAGGGATCGCCTGGCTGTTCTCCCGCG
GCACTCACAGCCCGGGAAGACTATTTGGGTGGAACACAAACTTGTGAACATCCTTGATGCAGTCGAGCTGGTGGA
CGAGCAGGGCGTTAATTTGGAACTCACATTGGTGACACTAGATATTAATGAAAAATTTAGAGATATCACCAAGTT
CATTCCAGAGACCATTAGCGGCGCTAGTGATGCAACTCTAGTGATCAACACAGAACATATGCCGTCAATGTTTGT
CCCTGTGGGGGACGTCGTGCAGTACGGGTTCTTGAACCTCAGTGGAAAGCCAACACATAGGACCATGATGTACAA
TTTCCCTACAAAAGCAGGGCAGTGTGGAGGCGTGGTTACATCAGTCGGTAAGATTGTTGGTATTCACATTGGTGG
CAACGGGCGCCAAGGGTTCTGCGCTGGTTTGAAGAGGAGCTACTTTGCGAGTATGCAGGGTGAGATCCAATGGGT
GAAGCCTAACAAGGAAACTGGCAGACTAAACATCAATGGACCAACTCGCACTAAGTTGGAGCCTAGTGTATTTCA
TGATGTGTTTGAAGGCAACAAGGAACCAGCAGTTTTAACAAGTAAAGACCCTAGATTGGAGGTCGACTTTGAACA
AGCCCTGTTTTCCAAGTATGTGGGCAATGTTTTACACGAGCCCGATGAATATGTGACTCAAGCTGCCCTCCACTA
TGCGAATCAACTTAAACAATTGGACATAAACACTAGCAAGATGAGCATGGAGGAAGCGTGCTATGGCACTGAAAA
CCTGGAAGCAATAGACCTCTGCACTAGTGCTGGGTATCCATACAGTGCCCTTGGTATCAAGAAAAGAGACATTCT
CGACCCCATAACCAGGGATGTGTCTAAGATGAAATTCTACATGGATAAATACGGACTAGATCTGCCATACTCTAC
CTATGTGAAGGATGAACTTAGATCTCTGGATAAAATCAAGAAAGGAAAGTCACGCCTGATAGAGGCCAGCAGCTT
GAATGACTCTGTCTACCTCAGAATGACTTTTGGGCACCTTTACGAGGTGTTTCATGCTAACCCTGGTACTGTGAC
TGGCTCAGCAGTAGGTTGCAACCCAGACGTGTTTTGGAGTAAACTACCGATTCTGCTGCCTGGGTCACTCTTTGC
CTTTGACTACTCAGGATATGATGCTAGTCTCAGCCCGGTATGGTTCAGGGCTCTAGAAGTTGTGTTACGGGAGAT
TGGGTATTCAGAGGAGGCCGTGTCCCTAATAGAAGGAATCAACCACACCCACCATGTGTACCGGAATAAAACATA
CTGTGTACTTGGTGGGATGCCCTCAGGGTGCTCTGGTACTTCCATCTTCAATTCAATGATCAACAACATCATCAT
TAGAACCCTTTTGATCAAAACCTTTAAGGGAATAGACCTGGATGAGTTGAACATGGTGGCCTATGGGGACGATGT
GCTGGCCAGTTACCCTTTTCCTATTGATTGCCTTGAATTGGCTAAGACTGGCAAAGAGTATGGTTTGACCATGAC
TCCTGCAGACAAATCACCCTGTTTCAATGAAGTAACATGGGAGAATGCTACCTTCCTGAAGAGAGGGTTCTTGCC
AGACCACCAATTTCCATTCTTAATTCACCCTACGATGCCCATGAGAGAGATCCATGAGTCCATTCGATGGACTAA
GGACGCGCGTAACACCCAGGATCACGTGCGCTCCCTGTGTCTATTGGCATGGCACAATGGTAAGGATGAATATGA
AAAGTTTGTGAGTGCAATTAGATCAGTTCCAGTTGGAAAAGCGTTGGCCATTCCTAACTTTGAGAATCTGAGAAG

AAATTGGCTCGAATTGTTTTAATATTACAGTTTAAAGCTGAACCCCACTAGAAATCTGGTCGTGTTAATGACTAG
TGGGGGTAAATTTGTTATAACCGGAATAGC

>EV-A71/SP
TTAAAACAGCTGTGGGTTGTACCCACTCACAGGGCCCACGTGGCGCTAGCACTCTGGTTCTGCGGAACCTTTGTG
CGCCTGTTTTACGCCCCCCCCCCAATTTGCAACTTAGAAGCAATACACAACACTGATCAACAGCAGGCATGGCGC
ACCAGCTATGTCTTGATCAAGCACTTCTGTTTCCCCGGGCCGAGTATCAATAGACTGTTCACGCGGTTGAAGGAG
AAAGCGCCCGTTATCCGGCTAACTACTTCGAGAAACCTAGTAGCACCATTGAAGCTGCAGAGTGCTTCGCTCGGC
ACTTCCCCCGTGTAGATCAGGTCGATGAGTCACTGCAATCCCCACGGGCGACCGTGGCAGTGGCTGCGCTGGCGG
CCTGCCTATGGGGCAACCCATAGGACGCTCTAATGTGGACATGGTGCGAAGAGTCTATTGAGCTAGTTAGTAGTC
CTCCGGCCCCTGAATGCGGCTAATCCTAACTGTGGAGCACATGCCTTCAATCCAGAGGGTAGTGTGTCGTAATGG
GCAACTCTGCAGCGGAACCGACTACTTTGGGTGTCCGTGTTTCCTTTTATCTTTACATTGGCTGCTTATGGTGAC
GATTATAGAATTGTTACCATATAGCTATTGGATTGGCCATCCGGTGTGCAATAGAGCTATTATATACCTGTTTGT
TGGCTTTGTACCACTAACCTTAAAATCTATAACCACCCTCGATTTTATATTAACCCTCAATACAATCAAACATGG
GCTCACAGGTGTCTACTCAGCGATCCGGCTCCCACGAGAACTCCAATTCAGCTACAGAAGGCTCCACCATTAATT
ACACTACCATCAACTATTACAAAGACTCCTATGCTGCGACAGCGGGCAAACAGAGCCTCAAGCAAGACCCTGATA
AATTTGCTAACCCTGTCAAGGACATTTTCACTGAAATGGCTGCACCACTGAAGTCTCCATCCGCTGAGGCTTGTG
GTTACAGTGATCGCGTGGCACAACTCACCATTGGAAACTCCACCATCACTACACAGGAGGCGGCGAATATCATAG
TCGGTTATGGTGAGTGGCCCTCATACTGCTCTGATGACGATGCTACAGCGGTGGACAAGCCAACGCGCCCAGATG
TTTCAGTGAATAGGTTTTATACGTTGGATACTAAATTGTGGGAAAAGTCATCCAAGGGGTGGTATTGGAAGTTTC
CTGATGTACTGACTGAGACCGGAGTCTTTGGCCAGAATGCACAGTTTCACTATTTATATAGGTCAGGATTTTGCA
TTCATGTGCAATGTAATGCTAGCAAGTTCCATCAAGGAGCGTTGTTAGTCGCCATACTTCCAGAGTATGTTATAG
GGACAGTGGCAGGCGGCACAGGAACTGAGGACAGCCACCCTCCTTACAAACAAACACAACCTGGCGCCGATGGTT
TTGAGTTGCAGCACCCGTACGTACTCGATGCTGGGATTCCTATATCACAATTAACAGTGTGCCCCCACCAATGGA
TTAACCTACGGACCAATAACTGTGCCACAATAATAGTGCCGTATATGAACACACTGCCTTTCGACTCTGCCCTGA
ACCATTGCAACTTTGGGCTGTTGGTGGTGCCCATTAGCCCACTAGATTTTGACCAAGGGGCAACTCCGGTTATCC
CTATTACAATCACTCTAGCTCCAATGTGCTCTGAGTTTGCAGGTCTCAGACAGGCGGTCACTCAAGGTTTTCCCA
CCGAGCCAAAACCAGGAACGAATCAATTTTTGACCACCGATGACGGTGTCTCAGCACCCATTTTACCAAATTTCC
ACCCCACACCATGTATTCACATACCCGGTGAAGTCAGAAACCTGCTTGAGTTGTGTCAAGTGGAGACCATTCTTG
AGGTTAACAATGTACCCACCAATGCCACCAGTCTGATGGAAAGGCTACGATTCCCGGTGTCCGCGCAAGCGGGAA
AAGGTGAATTGTGTGCCGTGTTTAGGGCCGACCCTGGAAGAGACGGTCCATGGCAATCAACAATGCTGGGCCAGT
TGTGTGGATATTACACCCAGTGGTCAGGATCACTGGAGGTTACTTTTATGTTCACCGGGTCTTTCATGGCCACGG
GTAAAATGCTCATAGCTTATACACCTCCTGGTGGCCCCTTACCCAAAGATCGGGCCACAGCAATGCTGGGCACAC
ATGTTATCTGGGATTTTGGGCTACAATCATCTGTCACCCTTGTAATACCATGGATTAGCAACACCCACTACAGAG
CGCATGCCCGGGATGGAGTGTTCGATTACTATACCACAGGACTGGTTAGTATCTGGTATCAAACAAACTACGTGG
TTCCAATTGGGGCACCCAACACAGCTTACATAATAGCACTAGCGGCAGCCCAGAAGAATTTTACCATGAAACTGT
GTAAAGACACCAGTCACATATTACAGACAGCCTCTATTCAGGGAGATAGAGTGGCAGATGTGATAGAGAGCTCTA
TAGGAGATAGTGTGAGTAGGGCACTTACCCAGGCCCTGCCAGCTCCAACAGGCCAGAACACGCAGGTGAGCAGTC
ATCGACTAGACACTGGTGAAGTTCCAGCGCTCCAAGCTGCTGAAATAGGGGCATCGTCAAATACTAGTGATGAGA
GTATGATTGAGACACGATGCGTTCTTAATTCACACAGTACGGCAGAGACCACCCTGGACAGCTTCTTCAGTAGGG
CAGGCTTGGTAGGAGAGATAGATCTCCCTCTTGAGGGTACCACTAATCCAAGTGGTTATGCTAATTGGGATATAG
ACATAACTGGTTACGCACAAATGCGCAGGAAAGTGGAGCTGTTCACCTACATGCGCTTTGATGCGGAATTCACTT
TTGTTGCGTGCACTCCTACTGGTCAGGTTGTCCCACAATTACTTCAGTATATGTTTGTTCCCCCTGGTGCTCCCA
AACCAGAGTCTAGAGAATCACTTGCTTGGCAGACAGCCACAAACCCCTCAGTTTTTGTCAAGTTGACTGATCCCC
CGGCACAGGTCTCAGTTCCGTTCATGTCACCCGCGAGCGCTTACCAGTGGTTTTACGACGGGTACCCCACGTTTG
GAGAACACAAACAGGAGAAAGACCTTGAGTATGGAGCGTGCCCTAATAATATGATGGGCACTTTCTCGGTGCGAA
ATGTGGGTTCATCAAAGTCCAAGTATCCTTTGGTTGTCAGGATATATATGAGAATGAAGCATGTCAGGGCGTGGA
TACCTCGCCCGATGCGCAACCAAAACTACCTGTTTAAAGCCAATCCAAACTATGCCGGTGACTCCATCAAACCGA
CCGGCACTAGTCGTACTGCCATTACTACCCTTGGAAAGTTCGGCCAGCAATCTGGGGCCATCTACGTGGGCAACT
TCAGAGTGGTTAATCGTCACCTCGCTACTCATAATGACTGGGCGAACCTCGTCTGGGAAGATAGCTCCCGCGACC
TATTAGTGTCGTCTACCACCGCCCAGGGCTGTGATACAATTGCACGTTGTGACTGTCAAACAGGAGTGTACTATT
GTAATTCCAAAAGAAAGCACTATCCAGTCAGCTTCTCCAAACCCAGCCTCATATATGTGGAGGCTAGCGAGTATT
ACCCTGCTAGATACCAATCGCACCTGATGCTTGCAGCAGGCCACTCTGAGCCCGGCGACTGCGGGGGCATCTTAA
GGTGTCAACATGGTGTAGTTGGTATAGTGTCCACGGGTGGCAACGGGCTCGTTGGTTTTGCTGATGTGAGGGATC
TCTTGTGGTTGGATGAAGAGGCCATGGAGCAAGGTGTGTCTGACTACATTAAGGGGCTCGGTGACGCATTTGGAA
CAGGTTTCACTGATGCTGTATCCAGGGAAGTTGAAGCCCTCAGGAACCACCTCATAGGATCTGATGGAGCAGTGG
AAAAAATCCTAAAGAACCTTATTAAGCTGATTTCAGCGTTAGTAATTGTGATTAGGAGCGATTATGATATGGTCA
CCCTCACAGCAACTTTAGCCCTGATTGGTTGTCATGGAAGTCCCTGGGCTTGGATTAAAGCCAAAACAGCATCCA
TTTTAGGTATCCCCATCGCCCAGAAGCAGAGCGCTTCTTGGCTAAAGAAATTTAATGATATGGCGAGTGCTGCCA
AGGGTTTAGAATGGATATCCAACAAAATTACTAAGTTCATTGACTGGCTCAGGGAGAAGATTGTTCCAGCAGCTA
AAGAGAAAGCAGAATTTTTAACCAATTTGAAGCAATTACCACTATTAGAGAACCAGATCACGAACTTGGAGCAGT

CCGCTGCCTCGCAAGAGGACCTTGAAGCTATGTTTGGGAATGTGTCATACCTCGCCCATTTCTGTCGCAAGTTCC
AACCATTATACGCCACAGAGGCCAAGCGAGTCTATGTTCTAGAGAAGAGAATGAACAATTACATGCAGTTCAAGA
GCAAACACCGTATTGAACCTGTATGTCTCATCATTAGAGGCTCACCAGGCACTGGAAAGTCCCTTGCGACCGGCA
TCATTGCCCGGGCCATAGCAGACAAGTACCACTCTAGTGTGTACTCACTCCCACCGGATCCTGACCATTTTGACG
GGTACAAACAGCAAGTGGTTACAGTTATGGATGACCTGTGCCAGAATCCTGACGGCAAAGACATGTCATTATTTT
GCCAGATGGTATCCACCGTGGATTTTATTCCACCAATGGCTTCTCTCGAAGAAAAGGGAGTTTCTTTCACATCTA
AATTTGTTATCGCATCCACCAACGCCAGCAACATTATAGTGCCCACAGTGTCTGACTCTGACGCCATTCGTCGCA
GGTTCTACATGGATTGCGACATTGAGGTCACAGACTCATACAAAACAGACTTGGGTAGACTAGACGCTGGGCGGG
CTGCTAAGTTATGCTCTGAAAACAACACCGCAAATTTCAAACGATGCAGCCCACTAGTGTGTGGGAAAGCTATTC
AACTTAGAGACAGGAAATCCAAGGTCAGGTATAGCGTGGACACAGTGGTCTCTGAACTTATTAGAGAATACAATA
GCAGATCCGCTATTGGTAACACAATTGAAGCATTATTCCAAGGCCCACCCAAGTTCAGGCCAATAAGGATCAGTC
TTGAGGAGAAGCCAGCCCCAGACGCTATTAGCGATCTCCTTGCTAGTGTGGATAGCGAGGAAGTGCGCCAATACT
GTAGGGAACAAGGCTGGATTATCCCTGAAACTCCCACCAATGTTGAACGACATCTTAATAGAGCAGTGCTAGTCG
TGCAATCCATCACTACTGTGGTGGCAGTCGTCTCACTGGTGTACGTCATTTACAAGCTCTTTGCGGGGTTTCAAG
GTGCGTATTCTGGAGCTCCCAAGCAAGTGCTCAAGAAGCCTGTCCTCCGCACGGCAACAGTGCAGGGTCCAAGCC
TTGATTTTGCCCTATCCTTGCTGAGGAGGAACATCAGGCAAGTCCAAACAGACCAAGGGCATTTTACCATGTTGG
GTGTCAGGGATCGCCTGGCTGTTCTCCCGCGGCACTCACAGCCCGGGAAGACTATTTGGGTGGAACACAAACTTG
TGAACATCCTTGATGCAGTCGAGCTGGTGGACGAGCAGGGCGTTAATTTGGAACTCACATTGGTGACACTAGATA
TTAATGAAAAATTTAGAGATATCACCAAGTTCATTCCAGAGACCATTAGCGGCGCTAGTGATGCAACTCTAGTGA
TCAACACAGAACATATGCCGTCAATGTTTGTCCCTGTGGGGGACGTCGTGCAGTACGGGTTCTTGAACCTCAGTG
GAAAGCCAACACATAGGACCATGATGTACAATTTCCCTACAAAAGCAGGACAGTGTGGAGGCGTGGTTACATCAG
TCGGTAAGATTGTTGGTATTCACATTGGTGGCAACGGGCGCCAAGGGTTCTGCGCTGGTTTGAAGAGGAGCTACT
TTGCGAGTATGCAAGGTGAGATCCAATGGGTGAAGCCTAACAAGGAAACTGGCAGACTAAACATCAATGGACCAA
CTCGCACTAAGTTGGAGCCTAGTGTATTTCATGATGTGTTTGAAGGCAACAAGGAACCAGCAGTTTTAACAAGTA
AAGACCCTAGATTGGAGGTCGACTTTGAACAAGCCCTGTTTTCCAAGTATGTGGGCAATGTTTTACACGAGCCCG
ATGAATATGTGACTCAAGCTGCCCTCCACTATGCGAATCAACTTAAACAATTGGACATAAACACTAGCAAGATGA
GCATGGAGGAAGCGTGCTATGGCACTGAAAACCTGGAAGCAATAGACCTCTGCACTAGTGCTGGGTATCCATACA
GTGCCCTTGGTATCAAGAAAAGAGACATTCTCGACCCCATAACCAGGGATGTGTCTAAGATGAAATTCTACATGG
ATAAATACGGACTAGATCTGCCATACTCTACCTATGTGAAGGATGAACTTAGATCTCTGGATAAAATCAAGAAAG
GAAAGTCACGCCTGATAGAGGCCAGCAGCTTGAATGACTCTGTCTACCTCAGAATGACTTTTGGCACCTTTACG
AGGTGTTTCATGCTAACCCTGGTACTGTGACTGGCTCAGCAGTAGGTTGCAACCCAGACGTGTTTTGGAGTAAAC
TACCGATTCTGCTGCCTGGGCCACTCTTTGCCTTTGACTACTCAGGATATGATGCTAGTCTCAGCCCGGTATGGT
TCAGGGCTCTAGAAGTTGTGTTACGGGAGATTGGGTATTCAGAGGAGGCCGTGTCCCTAATAGAAGGAATCAACC
ACACCCACCATGTGTACCGGAATAAAACATACTGTGTACTTGGTGGGATGCCCTCAGGGTGCTCTGGTACTTCCA
TCTTCAATTCAATGATCAACAACATCATCATTAGAACCCTTTTGATCAAAACCTTTAAGGGAATAGACCTGGATG
AGTTGAACATGGTGGCCTATGGGGACGATGTGCTGGCCAGTTACCCTTTTCCTATTGATTGCCTTGAATTGGCTA
AGACTGGCAAAGAGTATGGTTTGACCATGACTCCTGCAGACAAATCACCCTGTTTCAATGAAGTAACATGGGAGA
ATGCTACCTTCCTGAAGAGAGGGTTCTTGCCAGACCACCAATTTCCATTCTTAATTCACCCTACGATGCCCATGA
GAGAGATCCATGAGTCCATTCGATGGACTAAGGACGCGCGTAACACCCAGGATCACGTGCGCTCCCTGTGTCTAT
TGGCATGGCACAATGGTAAGGATGAATATGAAAAGTTTGTGAGTGCAATTAGATCAGTTCCAGTTGGAAAAGCGT
TGGCCATTCCTAACTTTGAGAATCTGAGAAGAAATTGGCTCGAATTGTTTTAATATTACAGTTTAAAGCTGAACC
CCACTAGAAATCTGGTCGTGTTAATGACTAGTGGGGGTAAATTTGTTATAACCGGAATAGC

>EV-A71/MP
TTAAAACAGCTGTGGGTTGTACCCACTCACAGGGCCCACGTGGCGCTAGCACTCTGGTTCTGCGGAACCTTTGTG
CGCCTGTTTTACGCCCCCCCCCCAATTTGCAACTTAGAAGCAATACACAACACTGATCAACAGCAGGCATGGCGC
ACCAGCTATGTCTTGATCAAGCACTTCTGTTTCCCCGGGCCGAGTATCAATAGACTGTCCACGCGGTTGAAGGAG
AAAGCGCCCGTTATCCGGCTAACTACTTCGAGAAACCTAGTAGCACCATTGAAGCTGCAGAGTGCTTCGCTCGGC
ACTTCCCCCGTGTAGATCAGGTCGATGAGTCACTGCAATCCCCACGGGCGACCGTGGCAGTGGCTGCGCTGGCGG
CCTGCCTATGGGGCAACCCATAGGACGCTCTAATGTGGACATGGTGCGAAGAGTCTATTGAGCTAGTTAGTAGTC
CTCCGGCCCCTGAATGCGGCTAATCCTAACTGTGGAGCACATGCCTTCAATCCAGAGGGTAGTGTGTCGTAATGG
GCAACTCTGCAGCGGAACCGACTACTTTGGGTGTCCGTGTTTCCTTTTATCTTTACATTGGCTGCTTATGGTGAC
GATTATAGAATTGTTACCATATAGCTATTGGATTGGCCATCCGGTGTGCAATAGAGCTATTATATACCTGTTTGT
TGGCTTTGTACCACTAACCTTAAAATCTATAACCACCCTCGATTTTATATTAACCCTCAATACAATCAAACATGG
GCTCACAGGTGTCTACTCAGCGATCCGGCTCCCACGAGAACTCCAATTCAGCTACAGAAGGCTCCACCATTAATT
ACACTACCATCAACTATTACAAAGACTCCTATGCTGCGACAGCGGGCAAACAGAGCCTCAAGCAAGACCCTGATA
AATTTGCTAACCCTGTCAAGGACATTTTCACTGAAATGGCTGCACCACTGAAGTCTCCATCCGCTGAGGCTTGTG
GTTACAGTGATCGCGTGGCACAACTCACCATTGGAAACTCCACCATCACTACACAGGAGGCGGCGAATATCATAG
TCGGTTATGGTGAGTGGCCCTCATACTGCTCTGATGACGATGCTACAGCGGTGGACAAGCCAACGCGCCCAGATG
TTTCAGTGAATAGGTTTTATACGTTGGATACTAAATTGTGGGAAAAGTCATCCAAGGGGTGGTATTGGAAGTTTC
CTGATGTACTGACTGAGACCGGAGTCTTTGGCCAGAATGCACAGTTTCACTATTTATATAGGTCAGGATTTTGCA

TTCATGTGCAATGTAATGCTAGCAAGTTCCATCAAGGAGCGTTGTTAGTCGCCATACTTCCAGAGTATGTTATAG
GGACAGTGGCAGGCGGCACAGGAACTGAGGACAGCCACCCTCCTTACAAACAAACACAACCTGGCGCCGATGGTT
TTGAGTTGCAGCACCCGTACGTACTCGATGCTGGGATTCCTATATCACAATTAACAGTGTGCCCCCACCAATGGA
TTAACCTACGGACCAATAACTGTGCCACAATAATAGTGCCGTATATGAACACACTGCCTTTCGACTCTGCCCTGA
ACCATTGCAACTTTGGGCTGTTGGTGGTGCCCATTAGCCCACTAGATTTTGACCAAGGGGCAACTCCGGTTATCC
CTATTACAATCACTCTAGCTCCAATGTGCTCTGAGTTTGCAGGTCTCAGACAGGCGGTCACTCAAGGTTTTCCCA
CCGAGCCAAAACCAGGAACGAATCAATTTTTGACCACCGATGACGGTGTCTCAGCACCCATTTTACCAAATTTCC
ACCCCACACCATGTATTCACATACCCGGTGAAGTCAGAAACCTGCTTGAGTTGTGTCAAGTGGAGACCATTCTTG
AGGTTAACAATGTACCCACCAATGCCACCAGTCTGATGGAAAGGCTACGATTCCCGGTGTCCGCGCAAGCGGGAA
AAGGTGAATTGTGTGCCGTGTTTAGGGCCGACCCTGGAAGAGACGGTCCATGGCAATCAACAATGCTGGGCCAGT
TGTGTGGATATTACACCCAGTGGTCAGGATCACTGGAGGTTACTTTTATGTTCACCGGGTCTTTCATGGCCACGG
GTAAAATGCTCATAGCTTATACACCTCCTGGTGGCCCCTTACCCAAAGATCGGGCCACAGCAATGCTGGGCACAC
ATGTTATCTGGGATTTTGGGCTACAATCATCTGTCACCCTTGTAATACCATGGATTAGCAACACCCACTACAGAG
CGCATGCCCGGGATGGAGTGCTCGATTACTATACCACAGGACTGGTTAGTATCTGGTATCAAACAAACTACGTGG
TTCCAATTGGGGCACCCAACACAGCTTACATAATAGCACTAGCGGCAGCCCAGAAGAATTTTACCATGAAACTGT
GTAAAGACACCAGTCACATATTACAGACAGCCTCTATTCAGGGAGATAGAGTGGCAGATGTGATAGAGAGCTCTA
TAGGAGATAGTGTGAGTAGGGCACTTACCCAGGCCCTGCCAGCTCCAACAGGTCAGAACACGCAGGTGAGCAGTC
ATCGACTAGACACTGGTGAAGTTCCAGCGCTCCAAGCTGCTGAAATAGGGGCATCGTCAAATACTAGTGATGAGA
GTATGATTGAGACACGATGCGTTCTTAATTCACACAGTACGGCAGAGACCACCCTGGACAGCTTCTTCAGTAGGG
CAGGCTTGGTAGGAGAGATAGATCTCCCTCTTGAGGGTACCACTAATCCAAGTGGTTATGCTAATTGGGATATAG
ACATAACTGGTTACGCACAAATGCGCAGGAAAGTGGAGCTGTTCACCTACATGCGCTTTGATGCGGAATTCACTT
TTGTTGCGTGCACTCCTACTGGTCAGGTTGTCCCACAATTACTTCAGTATATGTTTGTTCCCCCTGGTGCTCCCA
AACCAGAGTCTAGAGAATCACTTGCTTGGCAGACAGCCACAAACCCCTCAGTTTTTGTCAAGTTGACTGATCCCC
CGGCACAGGTCTCAGTTCCGTTCATGTCACCCGCGAGCGCTTACCAGTGGTTTTACGACGGGTACCCCACGTTTG
GAGAACACAAACAGGAGAAAGACCTTGAGTATGGAGCGTGCCCTAATAATATGATGGGCACTTTCTCGGTGCGAA
ATGTGGGTTCATCAAAGTCCAAGTATCCTTTGGTTGTCAGGATATATATGAGAATGAAGCATGTCAGGGCGTGGA
TACCTCGCCCGATGCGCAACCAAAACTACCTGTTTAAAGCCAATCCAAACTATGCCGGTGACTCCATCAAACCGA
CCGGCACTAGTCGTACTGCCATTACTACCCTTGGAAAGTTCGGCCAGCAATCTGGGGCCATCTACGTGGGCAACT
TCAGAGTGGTTAATCGTCACCTCGCTACTCATAATGACTGGGCGAACCTCGTCTGGGAAGATAGCTCCCGCGACC
TATTAGTGTCGTCTACCACCGCCCAGGGCTGTGATACAATTGCACGTTGTGACTGTCAAACAGGAGTGTACTATT
GTAATTCCAAAAGAAAGCACTATCCAGTCAGCTTCTCCAAACCCAGCCTCATATATGTGGAGGCTAGCGAGTATT
ACCCTGCTAGATACCAATCGCACCTGATGCTTGCAGCAGGCCACTCTGAGCCCGGCGACTGCGGGGGCATCTTAA
GGTGTCAACATGGTGTAGTTGGTATAGTGTCCACGGGTGGCAACGGGCTCGTTGGTTTTGCTGATGTGAGGGATC
TCTTGTGGTTGGATGAAGAGGCCATGGAGCAAGGTGTGTCTGACTACATTAAGGGGCTCGGTGACGCATTTGGAA
CAGGTTTCACTGATGCTGTATCCAGGGAAGTTGAAGCCCTCAGGAACCACCTCATAGGATCTGATGGAGCAGTGG
AAAAAATCCTAAAGAACCTTATTAAGCTGATTTCAGCGTTAGTAATTGTGATTAGGAGCGATTATGATATGGTCA
CCCTCACAGCAACTTTAGCCCTGATTGGTTGTCATGGAAGTCCCTGGGCTTGGATTAAAGCCAAAACAGCATCCA
TTTTAGGTATCCCCATCGCCCAGAAGCAGAGCGCTTCTTGGCTAAAGAAATTTAATGATATGGCGAGTGCTGCCA
AGGGTTTAGAATGGATATCCAACAAAATTAGTAAGTTCATTGACTGGCTCAGGGAGAAGATTGTTCCAGCAGCTA
AAGAGAAAGCAGAATTTTTAACCAATTTGAAGCAATTACCACTATTAGAGAACCAGATCACGAACTTGGAGCAGT
CCGCTGCCTCGCAAGAGGACCTTGAAGCTATGTTTGGGAATGTGTCATACCTCGCCCATTTCTGTCGCAAGTTCC
AACCATTATACGCCACGGAGGCCAAGCGAGTCTATGTTCTAGAGAAGAGAATGAACAATTACATGCAGTTCAAGA
GCAAACACCGTATTGAACCTGTATGTCTCATCATTAGAGGCTCACCAGGCACTGGAAAGTCCCTTGCGACCGGCA
TCATTGCCCGGGCCATAGCAGACAAGTACCACTCTAGTGTGTACTCACTCCCACCGGATCCTGACCATTTTGACG
GGTACAAACAGCAAGTGGTTACAGTTATGGATGACCTGTGCCAGAATCCTGACGGCAAAGACATGTCATTATTTT
GCCAGATGGTATCCACCGTGGATTTTATTCCACCAATGGCTTCTCTCGAAGAAAAGGGAGTTTCTTTCACATCTA
AATTTGTTATCGCATCCACCAACGCCAGCAACATTATAGTGCCCACAGTGTCTGACTCTGACGCCATTCGTCGCA
GGTTCTACATGGATTGCGACATTGAGGTCACAGACTCATACAAAACAGACTTGGGTAGACTAGACGCTGGGCGGG
CTGCTAAGTTATGCTCTGAAAACAACACCGCAAATTTCAAACGATGCAGCCCACTAGTGTGTGGGAAAGCTATTC
AACTTAGAGACAGGAAATCCAAGGTCAGGTATAGCGTGGACACAGTGGTCTCTGAACTTATTAGAGAATACAATA
GCAGATCCGCTATTGGTAACACAATTGAAGCATTATTCCAAGGCCCACCCAAGTTCAGGCCAATAAGGATCAGTC
TTGAGGAGAAGCCAGCCCCAGACGCTATTAGCGATCTCCTTGCTAGTGTGGATAGCGAGGAAGTGCGCCAATACT
GTAGGGAACAAGGCTGGATTATCCCTGAAACTCCCACCAATGTTGAACGACATCTTAATAGAGCAGTGCTAGTCG
TGCAATCCATCACTACTGTGGTGGCAGTCGTCTCACTGGTGTACGTCATTTACAAGCTCTTTGCGGGGTTTCAAG
GTGCGTATTCTGGAGCTCCCAAGCAAGTGCTCAAGAAGCCTGTCCTCCGCACGGCAACAGTGCAGGGTCCAAGCC
TTGATTTTGCCCTATCCTTGCTGAGGAGGAACATCAGGCAAGTCCAAACAGACCAAGGGCATTTTACCATGTTGG
GTGTCAGGGATCGCCTGGCTGTTCTCCCGCGGCACTCACAGCCCGGGAAGACTATTTGGGTGGAACACAAACTTG
TGAACATCCTTGATGCAGTCGAGCTGGTGGACGAGCAGGGCGTTAATTTGGAACTCACATTGGTGACACTAGATA
TTAATGAAAAATTTAGAGATATCACCAAGTTCATTCCAGAGACCATTAGCGGCGCTAGTGATGCAACTCTAGTGA
TCAACACAGAACATATGCCGTCAATGTTTGTCCCTGTGGGGGACGTCGTGCAGTACGGGTTCTTGAACCTCAGTG
GAAAGCCAACACATAGGACCATGATGTACAATTTCCCTACAAAAGCAGGACAGTGTGGAGGCGTGGTTACATCAG

TCGGTAAGATTGTTGGTATTCACATTGGTGGCAACGGGCGCCAAGGGTTCTGCGCTGGTTTGAAGAGGAGCTACT
TTGCGAGTATGCAAGGTGAGATCCAATGGGTGAAGCCTAACAAGGAAACTGGCAGACTAAACATCAATGGACCAA
CTCGCACTAAGTTGGAGCCTAGTGTATTTCATGATGTGTTTGAAGGCAACAAGGAACCAGCAGTTTTAACAAGTA
AAGACCCTAGATTGGAGGTCGACTTTGAACAAGCCCTGTTTTCCAAGTATGTGGGCAATGTTTTACACGAGCCCG
ATGAATATGTGACTCAAGCTGCCCTCCACTATGCGAATCAACTTAAACAATTGGACATAAACACTAGCAAGATGA
GCATGGAGGAAGCGTGCTATGGCACTGAAAACCTGGAAGCAATAGACCTCTGCACTAGTGCTGGGTATCCATACA
GTGCCCTTGGTATCAAGAAAAGAGACATTCTCGACCCCATAACCAGGGATGTGTCTAAGATGAAATTCTACATGG
ATAAATACGGACTAGATCTGCCATACTCTACCTATGTGAAGGATGAACTTAGATCTCTGGATAAAATCAAGAAAG
GAAAGTCACGCCTGATAGAGGCCAGCAGCTTGAATGACTCTGTCTACCTCAGAATGACTTTTGGGCACCTTTACG
AGGTGTTTCATGCTAACCCTGGTACTGTGACTGGCTCAGCAGTAGGTTGCAACCCAGACGTGTTTTGGAGTAAAC
TACCGATTCTGCTGCCTGGGTCACTCTTTGCCTTTGACTACTCAGGATATGATGCTAGTCTCAGCCCGGTATGGT
TCAGGGCTCTAGAAGTTGTGTTACGGGAGATTGGGTATTCAGAGGAGGCCGTGTCCCTAATAGAAGGAATCAACC
ACACCCACCATGTGTACCGGAATAAAACATACTGTGTACTTGGTGGGATGCCCTCAGGGTGCTCTGGTACTTCCA
TCTTCAATTCAATGATCAACAACATCATCATTAGAACCCTTTTGATCAAAACCTTTAAGGGAATAGACCTGGATG
AGTTGAACATGGTGGCCTATGGGGACGATGTGCTGGCCAGTTACCCTTTTCCTATTGATTGCCTTGAATTGGCTA
AGACTGGCAAAGAGTATGGTTTGACCATGACTCCTGCAGACAAATCACCCTGTTTCAATGAAGTAACATGGGAGA
ATGCTACCTTCCTGAAGAGAGGGTTCTTGCCAGACCACCAATTTCCATTCTTAATTCACCCTACGATGCCCATGA
GAGAGATCCATGAGTCCATTCGATGGACTAAGGACGCGCGTAACACCCAGGATCACGTGCGCTCCCTGTGTCTAT
TGGCATGGCACAATGGTAAGGATGAATATGAAAAGTTTGTGAGTGCAATTAGATCAGTTCCAGTTGGAAAAGCGT
TGGCCATTCCTAACTTTGAGAATCTGAGAAGAAATTGGCTCGAATTGTTTTAATATTACAGTTTAAAGCTGAACC
CCACTAGAAATCTGGTCGTGTTAATGACTAGTGGGGGTAAATTTGTTATAACCGGAATAGC

>EV-A71/BP
TTAAAACAGCTGTGGGTTGTACCCACTCACAGGGCCCACGTGGCGCTAGCACTCTGGTTCTGCGGAACCTTTGTG
CGCCTGTTTTACGCCCCCCCCCCAATTTGCAACTTAGAAGCAATACACAACACTGATCAACAGCAGGCATGGCGC
ACCAGCTATGTCTTGATCAAGCACTTCTGTTTCCCCGGGCCGAGTATCAATAGACTGTTCACGCGGTTGAAGGAG
AAAGCGCCCGTTATCCGGCTAACTACTTCGAGAAACCTAGTAGCACCATTGAAGCTGCAGAGTGTTTCGCTCGGC
ACTTCCCCCGTGTAGATCAGGTCGATGAGTCACTGCAATCCCCACGGGCGACCGTGGCAGTGGCTGCGCTGGCGG
CCTGCCTATGGGGCAACCCATAGGACGCTCTAATGTGGACATGGTGCGAAGAGTCTATTGAGCTAGTTAGTAGTC
CTCCGGCCCCTGAATGCGGCTAATCCTAACTGTGGAGCACATGCCTTCAATCCAGAGGGTAGTGTGTCGTAATGG
GCAACTCTGCAGCGGAACCGACTACTTTGGGTGTCCGTGTTTCCTTTTATCTTTACATTGGCTGCTTATGGTGAC
GATTATAGAATTGTTACCATATAGCTATTGGATTGGCCATCCGGTGTGCAATAGAGCTATTATATACCTGTTTGT
TGGCTTTGTACCACTAACCTTAAAATCTATAACCACCCTCGATTTTATATTAACCCTCAATACAATCAAACATGG
GCTCACAGGTGTCTACTCAGCGATCCGGCTCCCACGAGAACTCCAATTCAGCTACAGAAGGCTCCACCATTAATT
ACACTACCATCAACTATTACAAAGACTCCTATGCTGCGACAGCGGGCAAACAGAGCCTCAAGCAAGACCCTGATA
AATTTGCTAACCCTGTCAAGGACATTTTCACTGAAATGGCTGCACCACTGAAGTCTCCATCCGCTGAGGCTTGTG
GTTACAGTGATCGCGTGGCACAACTCACCATTGGAAACTCCACCATCACTACACAGGAGGCGGCGAATATCATAG
TCGGTTATGGTGAGTGGCCCTCATACTGCTCTGATGACGATGCTACAGCGGTGGACAAGCCAACGCGCCCAGATG
TTTCAGTGAATAGGTTTTATACGTTGGATACTAAATTGTGGGAAAAGTCATCCAAGGGGTGGTATTGGAAGTTTC
CTGATGTACTGACTGAGACCGGAGTCTTTGGCCAGAATGCACAGTTTCACTATTTATATAGGTCAGGATTTTGCA
TTCATGTGCAATGTAATGCTAGCAAGTTCCATCAAGGAGCGTTGTTAGTCGCCATACTTCCAGAGTATGTTATAG
GGACAGTGGCAGGCGGCACAGGAACTGAGGACAGCCACCCTCCTTACAAACAAACACAACCTGGCGCCGATGGTT
TTGAGTTGCAGCACCCGTACGTACTCGATGCTGGGATTCCTATATCACAATTAACAGTGTGCCCCCACCAATGGA
TTAACCTACGGACCAATAACTGTGCCACAATAATAGTGCCGTATATGAACACACTGCCTTTCGACTCTGCCCTGA
ACCATTGCAACTTTGGGCTGTTGGTGGTGCCCATTAGCCCACTAGATTTTGACCAAGGGGCAACTCCGGTTATCC
CTATTACAATCACTCTAGCTCCAATGTGCTCTGAGTTTGCAGGTCTCAGACAGGCGGTCACTCAAGGTTTTCCCA
CCGAGCCAAAACCAGGAACGAATCAATTTTTGACCACCGATGACGGTGTCTCAGCACCCATTTTACCAAATTTCT
ACCCCACACCATGTATTCACATACCCGGTGAAGTCAGAAACCTGCTTGAGTTGTGTCAAGTGGAGACCATTCTTG
AGGTTAACAATGTACCCACCAATGCCACCAGTCTGATGGAAAGGCTACGATTCCCGGTGTCCGCGCAAGCGGGAA
AAGGTGAATTGTGTGCCGTGTTTAGGGCCGACCCTGGAAGAGACGGTCCATGGCAATCAACAATGCTGGGCCAGT
TGTGTGGATATTACACCCAGTGGTCAGGATCACTGGAGGTTACTTTTATGTTCACCGGGTCTTTCATGGCCACGG
GTAAAATGCTCATAGCTTATACACCTCCTGGTGGCCCCTTACCCAAAGATCGGGCCACAGCAATGCTGGGCACAC
ATGTTATCTGGGATTTTGGGCTACAATCATCTGTCACCCTTGTAATACCATGGATTAGCAACACCCACTACAGAG
CGCATGCCCGGGATGGAGTGTTCGATTACTATACCACAGGACTGGTTAGTATCTGGTATCAAACAAACTACGTGG
TTCCAATTGGGGCACCCAACACAGCTTACATAATAGCACTAGCGGCAGCCCAGAAGAATTTTACCATGAAACTGT
GTAAAGACACCAGTCACATATTACAGACAGCCTCTATTCAGGGAGATAGAGTGGCAGATGTGATAGAGAGCTCTA
TAGGAGATAGTGTGAGTAGGGCACTTACCCAGGCCCTGCCAGCTCCAACAGGTCAGAACACGCAGGTGAGCAGTC
ATCGACTAGACACTGGTGAAGTTCCAGCGCTCCAAGCTGCTGAAATAGGGGCATCGTCAAATACTAGTGATGAGA
GTATGATTGAGACACGATGCGTTCTTAATTCACACAGTACGGCAGAGACCACCCTGGACAGCTTCTTCAGTAGGG
CAGGCTTGGTAGGAGAGATAGATCTCCCTCTTGAGGGTACCACTAATCCAAATGGTTATGCTAATTGGGATATAG
ACATAACTGGTTACGCACAAATGCGCAGGAAAGTGGAGCTGTTCACCTACATGCGCTTTGATGCGGAATTCACTT

TTGTTGCGTGCACTCCTACTGGTCAGGTTGTCCCACAATTACTTCAGTATATGTTTGTTCCCCCTGGTGCTCCCA
AACCAGAGTCTAGAGAATCACTTGCTTGGCAGACAGCCACAAACCCCTCAGTTTTTGTCAAGTCGACTGATCCCC
CGGCACAGGTCTCAGTTCCGTTCATGTCACCCGCGAGCGCTTACCAGTGGTTTTACGACGGGTACCCCACGTTTG
GAGAACACAAACAGGAGAAAGACCTTGAGTATGGAGCGTGCCCTAATAATATGATGGGCACTTTCTCGGTGCGAA
ATGTGGGTTCATCAAAGTCCAAGTATCCTTTGGTTGTCAGGATATATATGAGAATGAAGCATGTCAGGGCGTGGA
TACCTCGCCCGATGCGCAACCAAAACTACCTGTTTAAAGCCAATCCAAACTATGCCGGTAACTCCATCAAACCGA
CCGGCACTAGTCGTACTGCCATTACTACCCTTGGAAAGTTCGGCCAGCAATCTGGGGCCATCTACGTGGGCAACT
TCAGAGTGGTTAATCGTCACCTCGCTACTCATAATGACTGGGCGAACCTCGTCTGGGAAGATAGCTCCCGCGACC
TATTAGTGTCGTCTACCACCGCCCAGGGCTGTGATACAATTGCACGTTGTGACTGTCAAACAGGAGTGTACTATT
GTAATTCCAAAAGAAAGCACTATCCAGTCAGCTTCTCCAAACCCAGCCTCATATATGTGGAGGCTAGCGAGTATT
ACCCTGCTAGATACCAATCGCACCTGATGCTTGCAGCAGGCCACTCTGAGCCCGGCGACTGCGGGGGCATCTTAA
GGTGTCAACATGGTGTAGTTGGTATAGTGTCCACGGGTGGCAACGGGCTCGTTGGTTTTGCTGATGTGAGGGATC
TCTTGTGGTTGGATGAAGAGGCCATGGAGCAAGGTGTGTCTGACTACATTAAGGGGCTCGGTGACGCATTTGGAA
CAGGTTTCACTGATGCTGTATCCAGGGAAGTTGAAGCCCTCAGGAACCACCTCATAGGATCTGATGGAGCAGTGG
AAAAAATCCTAAAGAACCTTATTAAGCTGATTTCAGCGTTAGTAATTGTGATTAGGAGCGATTATGATATGGTCA
CCCTCACAGCAACTTTAGCCCTGATTGGTTGTCATGGAAGTCCCTGGGCTTGGATTAAAGCCAAAACAGCATCCA
TTTTAGGTATCCCCATCGCCCAGAAGCAGAGCGCTTCTTGGCTAAAGAAATTTAATGAAATGGCGAGTGCTGCCA
AGGGTTTAGAATGGATATCCAACAAAATTAGTAAGTTCATTGACTGGCTCAGGGAGAAGATTGTTCCAGCAGCTA
AAGAGAAAGCAGAATTTTTAACCAATTTGAAGCAATTACCACTATTAGAGAACCAGATCACGAACTTGGAGCAGT
CCGCTGCCTCGCAAGAGGACCTTGAAGCTATGTTTGGGAATGTGTCATACCTCGCCCATTTCTGTCGCAAGTTCC
AACCATTATACGCCACGGAGGCCAAGCGAGTCTATGTTCTAGAGAAGAGAATGAACAATTACATGCAGTTCAAGA
GCAAACACCGTATTGAACCTGTATGTCTCATCATTAGAGGCTCACCAGGCACTGGAAAGTCCCTTGCGACCGGCA
TCATTGCCCGGGCCATAGCAGACAAGTACCACTCTAGTGTGTACTCACTCCCACCGGATCCTGACCATTTTGACG
GGTACAAACAGCAAGTGGTTACAGTTATGGATGACCTGTGCCAGAATCCTGACGGCAAAGACATGTCATTATTTT
GCCAGATGGTATCCACCGTGGATTTTATTCCACCAATGGCTTCTCTCGAAGAAAAGGGAGTCTCTTTCACATCTA
AATTTGTTATCGCATCCACCAACGCCAGCAACATTATAGTGCCCACAGTGTCTGACTCTGACGCCATTCGTCGCA
GGTTCTACATGGATTGCGACATTGAGGTCACAGACTCATACAAAACAGACTTGGGTAGACTAGACGCTGGGCGGG
CTGCTAAGTTATGCTCTGAAAACAACACCGCAAATTTCAAACGATGCAGCCCACTAGTGTGTGGGAAAGCTATTC
AACTTAGAGACAGGAAATCCAAGGTCAGGTATAGCGTGGACACAGTGGTCTCTGAACTTATTAGAGAATACAATA
GCAGATCCGCTATTGGTAACACAATTGAAGCATTATTCCAAGGCCCACCCAAGTTCAGGCCAATAAGGATCAGTC
TTGAGGAGAAGCCAGCCCCAGACGCTATTAGCGATCTCCTTGCTAGTGTGGATAGCGAGGAAGTGCGCCAATACT
GTAGGGAACAAGGCTGGATTATCCCTGAAACTCCCACCAATGTTGAACGACATCTTAATAGAGCAGTGCTAGTCG
TGCAATCCATCACTACTGTGGTGGCAGTCGTCTCACTGGTGTACGTTATTTACAAGCTCTTTGCGGGGTTTCAAG
GTGCGTATTCTGGAGCTCCCAAGCAAGTGCTCAAGACGCCTGTCCTCCGCACGGCAACAGTGCAGGGTCCAAGCC
TTGATTTTGCCCTATCCTTGCTGAGGAGAAACATCAGGCAAGTCCAAACAGACCAAGGGCATTTTACCATGTTGG
GTGTCAGGGATCGCCTGGCTGTTCTCCCGCGGCACTCACAGCCCGGGAAGACTATTTGGGTGGAACACAAACTTG
TGAACATCCTTGATGCAGTCGAGCTGGTGGACGAGCAGGGCGTTAATTTGGAACTCACATTGGTGACACTAGATA
TTAATGAAAAATTTAGAGATATCACCAAGTTCATTCCAGAGACCATTAGCGGCGCTAGTGATGCAACTCTAGTGA
TCAACACAGAACATATGCCGTCAATGTTTGTCCCTGTGGGGGACGTCGTGCAGTACGGGTTCTTGAACCTCAGTG
GAAAGCCAACACATAGGACCATGATGTACAATTTCCCTACAAAAGCAGGACAGTGTGGAGGCGTGGTTACATCAG
TCGGTAAGATTGTTGGTATTCACATTGGTGGCAACGGGCGCCAAGGGTTCTGCGCTGGTTTGAAGAGGAGCTACT
TTGCGAGTATGCAAGGTGAGATCCAATGGGTGAAGCCTAACAAGGAAACTGGCAGACTAAACATCAATGGACCAA
CTCGCACTAAGTTGGAGCCTAGTGTATTTCATGATGTGTTTGAAGGCAACAAGGAACCAGCAGTTTTAACAAGTA
AAGACCCTAGATTGGAGGTCGACTTTGAACAAGCCCTGTTTTCCAAGTATGTGGGCAATGTTTTGCACGAGCCCG
ATGAATATGTGACTCAAGCTGCCCTCCACTATGCGAATCAACTTAAACAATTGGACATAAACACTAGCAAGATGA
GCATGGAGGAAGCGTGCTATGGCACTGAAAACCTGGAAGCAATAGACCTCTGCACTAGTGCTGGGTATCCATACA
GTGCCCTTGGTATCAAGAAAAGAGACATTCTCGACCCCATAACCAGGGATGTGTCTAAGATGAAATTCTACATGG
ATAAATACGGACTAGATCTGCCATACTCTACCTATGTGAAGGATGAACTTAGATCTCTGGATAAAATCAAGAAAG
GAAAGTCACGCCTGATAGAGGCCAGCAGCTTGAATGACTCTGTCTACCTCAGAATGACTTTTGGGCACCTTTACG
AGGTGTTTCATGCTAACCCTGGTACTGTGACTGGCTCAGCAGTAGGTTGCAACCCAGACGTGTTTTGGAGTAAAC
TACCGATTCTGCTGCCTGGGTCACTCTTTGCCTTTGACTACTCAGGATATGATGCTAGTCTCAGCCCGGTATGGT
TCAGGGCTCTAGAAGTTGTGTTACGGGAGATTGGGTATTCAGAGGAGGCCGTGTCCCTAATAGAAGGAATCAACC
ACACCCACCATGTGTACCGGAATAAAACATACTGTGTACTTGGTGGGATGCCCTCAGGGTGCTCTGGTACTTCCA
TCTTCAATTCAATGATCAACAACATCATCATTAGAACCCTTTTGATTAAAACCTTTAAGGGAATAGACCTGGATG
AGTTGAACATGGTGGCCTATGGGGACGATGTGCTGGCCAGTTACCCTTTTCCTATTGATTGCCTTGAATTGGCTA
AGACTGGCAAAGAGTATGGTTTGACCATGACTCCTGCAGACAAATCACCCTGTTTCAATGAAGTAACATGGGAGA
ATGCTACCTTCCTGAAGAGAGGGTTCTTGCCAGACCACCAATTTCCATTCTTAATTCACCCTACGATGCCCATGA
GAGAGATCCATGAGTCCATTCGATGGACTAAGGACGCGCGTAACACCCAGGATCACGTGCGTTCCCTGTGTCTAT
TGGCATGGCACAATGGTAAGGATGAATATGAAAAGTTTGTGAGTGCAATTAGATCAGTTCCAGTTGGAAAAGCGT
TGGCCATTCCTAACTTTGAGAATCTGAGAAGAAATTGGCTCGAATTGTTTTAATATTACAGTTTAAAGCTGAACC
CCACTAGAAATCTGGTCGTGTTAATGACTAGTGGGGGTAAATTTGTTATAACCGGAATAGC

>EV-A71/HP
TTAAAACAGCTGTGGGTTGTACCCACTCACAGGGCCCACGTGGCGCTAGCACTCTGGTTCTGCGGAACCTTTGTG
CGCCTGTTTTACGCCCCCCCCCCGATTTGCAACTTAGAAGCAATACACAACACTGATCAACAGCAGGCATGGCGC
ACCAGCTATGTCTTGATCAAGCACTTCTGTTTCCCCGGGCCGAGTATCAATAGACTGTTCACGCGGTTGAAGGAG
AAAGCGCCCGTTATCCGGCTAACTACTTCGAGAAACCTAGTAGCACCATTGAAGCTGCAGAGTGTTTCGCTCGGC
ACTTCCCCCGTGTAGATCAGGTCGATGAGTCACTGCAATCCCCACGGGCGACCGTGGCAGTGGCTGCGCTGGCGG
CCTGCCTATGGGGCAACCCATAGGACGCTCTAATGTGGACATGGTGCGAAGAGTCTATTGAGCTAGTTAGTAGTC
CTCCGGCCCCTGAATGCGGCTAATCCTAACTGTGGAGCACATGCCTTCAATCCAGAGGGTAGTGTGTCGTAATGG
GCAACTCTGCAGCGGAACCGACTACTTTGGGTGTCCGTGTTTCCTTTTATCTTTACATTGGCTGCTTATGGTGAC
GATTATAGAATTGTTACCATATAGCTATTGGATTGGCCATCCGGTGTGCAATAGAGCTATTATATACCTGTTTGT
TGGCTTTGTACCACTAACCTTAAAATCTATAACCACCCTCGATTTTATATTAACCCTCAATACAATCAAACATGG
GCTCACAGGTGTCTACTCAGCGATCCGGCTCCCACGAGAACTCCAATTCAGCTACAGAAGGCTCCACCATTAATT
ACACTACCATCAACTATTACAAAGACTCCTATGCTGCGACAGCGGGCAAACAGAGCCTCAAGCAAGACCCTGATA
AATTTGCTAACCCTGTCAAGGACATTTTCACTGAAATGGCTGCACCACTGAAGTCTCCATCCGCTGAGGCTTGTG
GTTACAGTGATCGCGTGGCACAACTCACCATTGGAAACTCCACCATCACTACACAGGAGGCGGCGAATATCATAG
TCGGTTATGGTGAGTGGCCCTCATACTGCTCTGATGACGATGCTACAGCGGTGGACAAGCCAACGCGCCCAGATG
TTTCAGTGAATAGGTTTTATACGTTGGATACTAAATTGTGGGAAAAGTCATCCAAGGGGTGGTATTGGAAGTTTC
CTGATGTACTGACTGAGACCGGAGTCTTTGGCCAGAATGCACAGTTTCACTATTTATATAGGTCAGGATTTTGCA
TTCATGTGCAATGTAATGCTAGCAAGTTCCATCAAGGAGCGTTGTTAGTCGCCATACTTCCAGAGTATGTTATAG
GGACAGTGGCAGGCGGCACAGGAACTGAGGACAGCCACCCTCCTTACAAACAAACACAACCTGGCGCCGATGGTT
TTGAGTTGCAGCACCCGTACGTACTCGATGCTGGGATTCCTATATCACAATTAACAGTGTGCCCCCACCAATGGA
TTAACCTACGGACCAATAACTGTGCCACAATAATAGTGCCGTATATGAACACACTGCCTTTCGACTCTGCCCTGA
ACCATTGCAACTTTGGGCTGTTGGTGGTGCCCATTAGCCCACTAGATTTTGACCAAGGGGCAACTCCGGTTATCC
CTATTACAATCACTCTAGCTCCAATGTGCTCTGAGTTTGCAGGTCTCAGACAGGCGGTCACTCAAGGTTTTCCCA
CCGAGCCAAAACCAGGAACGAATCAATTTTTGACCACCGATGACGGTGTCTCAGCACCCATTTTACCAAATTTCT
ACCCCACACCATGTATTCACATACCCGGTGAAGTCAGAAACCTGCTTGAGTTGTGTCAAGTGGAGACCATTCTTG
AGGTTAACAATGTACCCACCAATGCCACCAGTCTGATGGAAAGGCTACGATTCCCGGTGTCCGCGCAAGCGGGAA
AAGGTGAATTGTGTGCCGTGTTTAGGGCCGACCCTGGAAGAGACGGTCCATGGCAATCAACAATGCTGGGCCAGT
TGTGTGGATATTACACCCAGTGGTCAGGATCACTGGAGGTTACTTTTATGTTCACCGGGTCTTTCATGGCCACGG
GTAAAATGCTCATAGCTTATACACCTCCTGGTGGCCCCTTACCCAAAGATCGGGCCACAGCAATGCTGGGCACAC
ATGTTATCTGGGATTTTGGGCTACAATCATCTGTCACCCTTGTAATACCATGGATTAGCAACACCCACTACAGAG
CGCATGCCCGGGATGGAGTGTTCGATTACTATACCACAGGACTGGTTAGTATCTGGTATCAAACAAACTACGTGG
TTCCAATTGGGGCACCCAACACAGCTTACATAATAGCACTAGCGGCAGCCCAGAAGAATTTTACCATGAAACTGT
GTAAAGACACCAGTCACATATTACAGACAGCCTCTATTCAGGGAGATAGAGTGGCAGATGTGATAGAGAGCTCTA
TAGGAGATAGTGTGAGTAGGGCACTTACCCAGGCCCTGCCAGCTCCAACAGGTCAGAACACGCAGGTGAGCAGTC
ATCGACTAGACACTGGTGAAGTTCCAGCGCTCCAAGCTGCTGAAATAGGGGCATCGTCAAATACTAGTGATGAGA
GTATGATTGAGACACGATGCGTTCTTAATTCACACAGTACGGCAGAGACCACCCTGGACAGCTTCTTCAGTAGGG
CAGGCTTGGTAGGAGAGATAGATCTCCCTCTTGAGGGTACCACTAATCCAAATGGTTATGCTAATTGGGATATAG
ACATAACTGGTTACGCACAAATGCGCAGGAAAGTGGAGCTGTTCACCTACATGCGCTTTGATGCGGAATTCACTT
TTGTTGCGTGCACTCCTACTGGTCAGGTTGTCCCACAATTACTTCAGTATATGTTTGTTCCCCCTGGTGCTCCCA
AACCAGAGTCTAGAGAATCACTTGCTTGGCAGACAGCCACAAACCCCTCAGTTTTTGTCAAGTCGACTGATCCCC
CGGCACAGGTCTCAGTTCCGTTCATGTCACCCGCGAGCGCTTACCAGTGGTTTTACGACGGGTACCCCACGTTTG
GAGAACACAAACAGGAGAAAGACCTTGAGTATGGAGCGTGCCCTAATAATATGATGGGCACTTTCTCGGTGCGAA
ATGTGGGTTCATCAAAGTCCAAGTATCCTTTGGTTGTCAGGATATATATGAGAATGAAGCATGTCAGGGCGTGGA
TACCTCGCCCGATGCGCAACCAAAACTACCTGTTTAAAGCCAATCCAAACTATGCCGGTAACTCCATCAAACCGA
CCGGCACTAGTCGTACTGCCATTACTACCCTTGGAAAGTTCGGCCAGCAATCTGGGGCCATCTACGTGGGCAACT
TCAGAGTGGTTAATCGTCACCTCGCTACTCATAATGACTGGGCGAACCTCGTCTGGGAAGATAGCTCCCGCGACC
TATTAGTGTCGTCTACCACCGCCCAGGGCTGTGATACAATTGCACGTTGTGACTGTCAAACAGGAGTGTACTATT
GTAATTCCAAAAGAAAGCACTATCCAGTCAGCTTCTCCAAACCCAGCCTCATATATGTGGAGGCTAGCGAGTATT
ACCCTGCTAGATACCAATCGCACCTGATGCTTGCAGCAGGCCACTCTGAGCCCGGCGACTGCGGGGGCATCTTAA
GGTGTCAACATGGTGTAGTTGGTATAGTGTCCACGGGTGGCAACGGGCTCGTTGGTTTTGCTGATGTGAGGGATC
TCTTGTGGTTGGATGAAGAGGCCATGGAGCAAGGTGTGTCTGACTACATTAAGGGGCTCGGTGACGCATTTGGAA
CAGGTTTCACTGATGCTGTATCCAGGGAAGTTGAAGCCCTCAGGAACCACCTCATAGGATCTGATGGAGCAGTGG
AAAAAATCCTAAAGAACCTTATTAAGCTGATTTCAGCGTTAGTAATTGTGATTAGGAGCGATTATGATATGGTCA
CCCTCACAGCAACTTTAGCCCTGATTGGTTGTCATGGAAGTCCCTGGGCTTGGATTAAAGCCAAAACAGCATCCA
TTTTAGGTATCCCCATCGCCCAGAAGCAGAGCGCTTCTTGGCTAAAGAAATTTAATGAAATGGCGAGTGCTGCCA
AGGGTTTAGAATGGATATCCAACAAAATTAGTAAGTTCATTGACTGGCTCAGGGAGAAGATTGTTCCAGCAGCTA
AAGAGAAAGCAGAATTTTTAACCAATTTGAAGCAATTACCACTATTAGAGAACCAGATCACGAACTTGGAGCAGT
CCGCTGCCTCGCAAGAGGACCTTGAAGCTATGTTTGGGAATGTGTCATACCTCGCCCATTTCTGTCGCAAGTTCC
AACCATTATACGCCACGGAGGCCAAGCGAGTCTATGTTCTAGAGAAGAGAATGAACAATTACATGCAGTTCAAGA

GCAAACACCGTATTGAACCTGTATGTCTCATCATTAGAGGCTCACCAGGCACTGGAAAGTCCCTTGCGACCGGCA
TCATTGCCCGGGCCATAGCAGACAAGTACCACTCTAGTGTGTACTCACTCCCACCGGATCCTGACCATTTTGACG
GGTACAAACAGCAAGTGGTTACAGTTATGGATGACCTGTGCCAGAATCCTGACGGCAAAGACATGTCATTATTTT
GCCAGATGGTATCCACCGTGGATTTTATTCCACCAATGGCTTCTCTCGAAGAAAAGGGAGTCTCTTTCACATCTA
AATTTGTTATCGCATCCACCAACGCCAGCAACATTATAGTGCCCACAGTGTCTGACTCTGACGCCATTCGTCGCA
GGTTCTACATGGATTGCGACATTGAGGTCACAGACTCATACAAAACAGACTTGGGTAGACTAGACGCTGGGCGGG
CTGCTAAGTTATGCTCTGAAAACAACACCGCAAATTTCAAACGATGCAGCCCACTAGTGTGTGGGAAAGCTATTC
AACTTAGAGACAGGAAATCCAAGGTCAGGTATAGCGTGGACACAGTGGTCTCTGAACTTATTAGAGAATACAATA
GCAGATCCGCTATTGGTAACACAATTGAAGCATTATTCCAAGGCCCACCCAAGTTCAGGCCAATAAGGATCAGTC
TTGAGGAGAAGCCAGCCCCAGACGCTATTAGCGATCTCCTTGCTAGTGTGGATAGCGAGGAAGTGCGCCAATACT
GTAGGGAACAAGGCTGGATTATCCCTGAAACTCCCACCAATGTTGAACGACATCTTAATAGAGCAGTGCTAGTCG
TGCAATCCATCACTACTGTGGTGGCAGTCGTCTCACTGGTGTACGTTATTTACAAGCTCTTTGCGGGGTTTCAAG
GTGCGTATTCTGGAGCTCCCAAGCAAGTGCTCAAGACGCCTGTCCTCCGCACGGCAACAGTGCAGGGTCCAAGCC
TTGATTTTGCCCTATCCTTGCTGAGGAGAAACATCAGGCAAGTCCAAACAGACCAAGGGCATTTTACCATGTTGG
GTGTCAGGGATCGCCTGGCTGTTCTCCCGCGGCACTCACAGCCCGGGAAGACTATTTGGGTGGAACACAAACTTG
TGAACATCCTTGATGCAGTCGAGCTGGTGGACGAGCAGGGCGTTAATTTGGAACTCACATTGGTGACACTAGATA
TTAATGAAAAATTTAGAGATATCACCAAGTTCATTCCAGAGACCATTAGCGGCGCTAGTGATGCAACTCTAGTGA
TCAACACAGAACATATGCCGTCAATGTTTGTCCCTGTGGGGGACGTCGTGCAGTACGGGTTCTTGAACCTCAGTG
GAAAGCCAACACATAGGACCATGATGTACAATTTCCCTACAAAAGCAGGACAGTGTGGAGGCGTGGTTACATCAG
TCGGTAAGATTGTTGGTATTCACATTGGTGGCAACGGGCGCCAAGGGTTCTGCGCTGGTTTGAAGAGGAGCTACT
TTGCGAGTATGCAAGGTGAGATCCAATGGGTGAAGCCTAACAAGGAAACTGGCAGACTAAACATCAATGGACCAA
CTCGCACTAAGTTGGAGCCTAGTGTATTTCATGATGTGTTTGAAGGCAATAAGGAACCAGCAGTTTTAACAAGTA
AAGACCCTAGATTGGAGGTCGACTTTGAACAAGCCCTGTTTTCCAAGTATGTGGGCAATGTTTTGCACGAGCCCG
ATGAATATGTGACTCAAGCTGCCCTCCACTATGCGAATCAACTTAAACAATTGGACATAAACACTAGCAAGATGA
GCATGGAGGAAGCGTGCTATGGCACTGAAAACCTGGAAGCAATAGACCTCTGCACTAGTGCTGGGTATCCATACA
GTGCCCTTGGTATCAAGAAAAGAGACATTCTCGACCCCATAACCAGGGATGTGTCTAAGATGAAATTCTACATGG
ATAAATACGGACTAGATCTGCCATACTCTACCTATGTGAAGGATGAACTTAGATCTCTGGATAAAATCAAGAAAG
GAAAGTCACGCCTGATAGAGGCCAGCAGCTTGAATGACTCTGTCTACCTCAGAATGACTTTTGGGCACCTTTACG
AGGTGTTTCATGCTAACCCTGGTACTGTGACTGGCTCAGCAGTAGGTTGCAACCCAGACGTGTTTTGGAGTAAAC
TACCGATTCTGCTGCCTGGGTCACTCTTTGCCTTTGACTACTCAGGATATGATGCTAGTCTCAGCCCGGTATGGT
TCAGGGCTCTAGAAGTTGTGTTACGGGAGATTGGGTATTCAGAGGAGGCCGTGTCCCTAATAGAAGGAATCAACC
ACACCCACCATGTGTACCGGAATAAAACATACTGTGTACTTGGTGGGATGCCCTCAGGGTGCTCTGGTACTTCCA
TCTTCAATTCAATGATCAACAACATCATCATTAGAACCCTTTTGATCAAAACCTTTAAGGGAATAGACCTGGATG
AGTTGAACATGGTGGCCTATGGGGACGATGTGCTGGCCAGTTACCCTTTTCCTATTGATTGCCTTGAATTGGCTA
AGACTGGCAAAGAGTATGGTTTGACCATGACTCCTGCAGACAAATCACCCTGTTTCAATGAAGTAACATGGGAGA
ATGCTACCTTCCTGAAGAGAGGGTTCTTGCCAGACCACCAATTTCCATTCTTAATTCACCCTACGATGCCCATGA
GAGAGATCCATGAGTCCATTCGATGGACTAAGGACGCGCGTAACACCCAGGATCACGTGCGTTCCCTGTGTCTAT
TGGCATGGCACAATGGTAAGGATGAATATGAAAAGTTTGTGAGTGCAATTAGATCAGTTCCAGTTGGAAAAGCGT
TGGCCATTCCTAACTTTGAGAATCTGAGAAGAAATTGGCTCGAATTGTTTTAATATTACAGTTTAAAGCTGAACC
CCACTAGAAATCTGGTCGTGTTAATGACTAGTGGGGGTAAATTTGTTATAACCGGAATAGC

>EV-A71/VS
TTAAAACAGCTGTGGGTTGTACCCACTCACAGGGCCCACGTGGCGCTAGCACTCTGGTTCTGCGGAACCTTTGTG
CGCCTGTTTTACGCCCCCCCCCCAATTTGCAACTTAGAAGCAATACACAACACTGATCAACAGCAGGCATGGCGC
ACCAGCTATGTCTTGATCAAGCACTTCTGTTTCCCCGGGCCGAGTATCAATAGACTGTTCACGCGGTTGAAGGAG
AAAGCGCCCGTTATCCGGCTAACTACTTCGAGAAACCTAGTAGCACCATTGAAGCTGCAGAGTGTTTCGCTCGGC
ACTTCCCCCGTGTAGATCAGGTCGATGAGTCACTGCAATCCCCACGGGCGACCGTGGCAGTGGCTGCGCTGGCGG
CCTGCCTATGGGGCAACCCATAGGACGCTCTAATGTGGACATGGTGCGAAGAGTCTATTGAGCTAGTTAGTAGTC
CTCCGGCCCCTGAATGCGGCTAATCCTAACTGTGGAGCACATGCCTTCAATCCAGAGGGTAGTGTGTCGTAATGG
GCAACTCTGCAGCGGAACCGACTACTTTGGGTGTCCGTGTTTCCTTTTATCTTTACATTGGCTGCTTATGGTGAC
GATTATAGAATTGTTACCATATAGCTATTGGATTGGCCATCCGGTGTGCAATAGAGCTATTATATACCTGTTTGT
TGGCTTTGTACCACTAACCTTAAAATCTATAACCACCCTCGATTTTATATTAACCCTCAATACAATCAAACATGG
GCTCACAGGTGTCTACTCAGCGATCCGGCTCCCACGAGAACTCCAATTCAGCTACAGAAGGCTCCACCATTAATT
ACACTACCATCAACTATTACAAAGACTCCTATGCTGCGACAGCGGGCAAACAGAGCCTCAAGCAAGACCCTGATA
AATTTGCTAACCCTGTCAAGGACATTTTCACTGAAATGGCTGCACCACTGAAGTCTCCATCCGCTGAGGCTTGTG
GTTACAGTGATCGCGTGGCACAACTCACCATTGGAAACTCCACCATCACTACACAGGAGGCGGCGAATATCATAG
TCGGTTATGGTGAGTGGCCCTCATACTGCTCTGATGACGATGCTACAGCGGTGGACAAGCCAACGCGCCCAGATG
TTTCAGTGAATAGGTTTTATACGTTGGATACTAAATTGTGGGAAAAGTCATCCAAGGGGTGGTATTGGAAGTTTC
CTGATGTACTGACTGAGACCGGAGTCTTTGGCCAGAATGCACAGTTTCACTATTTATATAGGTCAGGATTTTGCA
TTCATGTGCAATGTAATGCTAGCAAGTTCCATCAAGGAGCGTTGTTAGTCGCCATACTTCCAGAGTATGTTATAG
GGACAGTGGCAGGCGGCACAGGAACTGAGGACAGCCACCCTCCTTACAAACAAACACAACCTGGCGCCGATGGTT

TTGAGTTGCAGCACCCGTACGTACTCGATGCTGGGATTCCTATATCACAATTAACAGTGTGCCCCCACCAATGGA
TTAACCTACGGACCAATAACTGTGCCACAATAATAGTGCCGTATATGAACACACTGCCTTTCGACTCTGCCCTGA
ACCATTGCAACTTTGGGCTGTTGGTGGTGCCCATTAGCCCACTAGATTTTGACCAAGGGGCAACTCCGGTTATCC
CTATTACAATCACTCTAGCTCCAATGTGCTCTGAGTTTGCAGGTCTCAGACAGGCGGTCATTCAAGGTTTTCCCA
CCGAGCCAAAACCAGGAACGAATCAATTTTTGACCACCGATGACGGTGTCTCAGCACCCATTTTACCAAATTTCT
ACCCCACACCATGTATTCACATACCCGGTGAAGTCAGAAACCTGCTTGAGTTGTGTCAAGTGGAGACCATTCTTG
AGGTTAACAATGTACCCACCAATGCCACCAGTCTGATGGAAAGGCTACGATTCCCGGTGTCCGCGCAAGCGGGAA
AAGGTGAATTGTGTGCCGTGTTTAGGGCCGACCCTGGAAGAGACGGTCCATGGCAATCAACAATGCTGGGCCAGT
TGTGTGGATATTACACCCAGTGGTCAGGATCACTGGAGGTTACTTTTATGTTCACCGGGTCTTTCATGGCCACGG
GTAAAATGCTCATAGCTTATACACCTCCTGGTGGCCCCTTACCCAGAGATCGGCCACAGCAATGCTGGGCACAC
ATGTTATCTGGGATTTTGGGCTACAATCATCTGTCACCCTTGTAATACCATGGATTAGCAACACCCACTACAGAG
CGCATGCCCGGGATGGAGTGTTCGATTACTATACCACAGGACTGGTTAGTATCTGGTATCAAACAAACTACGTGG
TTCCAATTGGGGCACCCAACACAGCTTACATAATAGCACTAGCGGCAGCCCAGAAGAATTTTACCATGAAACTGT
GTAAAGACACCAGTCACATATTACAGACAGCCTCTATTCAGGGAGATAGAGTGGCAGATGTGATAGAGAGCTCTA
TAGGAGATAGTGTGAGTAGGGCACTTACCCAGGCCCTGCCAGCTCCAACAGGTCAGAATACGCAGGTGAGCAGTC
ATCGACTAGACACTGGTGAAGTTCCAGCGCTCCAAGCTGCTGAAATAGGGGCATCGTCAAATACTAGTGATGAGA
GTATGATTGAGACACGATGCGTTCTTAATTCACACAGTACGGCAGAGACCACCCTGGACAGCTTCTTCAGTAGGG
CAGGCTTGGTAGGAGAGATAGATCTCCCTATTGAGGGTACCACTAATCCAAATGGTTATGCTAATTGGGATATAG
ACATAACTGGTTACGCACAAATGCGCAGGAAAGTGGAGCTGTTCACCTACATGCGCTTTGATGCGGAATTCACTT
TTATTGCGTGCACTCCTACTGGTCAGGTTGTCCCACAATTACTTCAGTATATGTTTGTTCCCCCTGGTGCTCCCA
AACCAGAGTCTAGAGAATCACTTGCTTGGCAGACAGCCACAAACCCCTCAGTTTTTGTCAAGTTGACTGATCCCC
CGGCACAGGTCTCAGTTCCGTTCATGTCACCCGCGAGCGCTTACCAGTGGTTTTACGACGGGTACCCCACGTTTG
GAGAACACAAACAGGAGAAAGACCTTGAGTATGGAGCGTGCCCTAATAATATGATGGGCACTTTCTCGGTGCGAA
CTGTGGGTTCATCAAAGTCCAAGTATTCTTTGGTTGTCAGGATATATATGAGAATGAAGCATGTCAGGGCGTGGA
TACCTCGCCCGATGCGCAACCAAAACTACCTGTTTAAAGCCAATCCAAACTATGCCGGTAACTCCATCAAACCGA
CCGGCACTAGTCGTACCGCCATTACTACCCTTGGAAAGTTCGGCCAGCAATCTGGGGCCATCTACGTGGGCAACT
TCAGAGTGGTTAATCGTCACCTCGCTACTCATAATGACTGGGCGAACCTCGTCTGGGAAGATAGCTCCCGCGACC
TATTAGTGTCGTCTACCACCGCCCAGGGCTGTGATACAATTGCACGTTGTGACTGTCAAACAGGAGTGTACTATT
GTAATTCCAAAAGAAAGCACTATCCAGTCAGCTTCTCCAAACCCAGCCTCATATATGTGGAGGCTAGCGAGTATT
ACCCTGCTAGATACCAATCGCACCTGATGCTTGCAGCAGGCCACTCTGAGCCCGGCGACTGCGGGGGCATTTTAA
GGTGTCAACATGGTGTAGTTGGTATAGTGTCCACGGGTGGCAACGGGCTTGTTGGTTTTGCTGATGTGAGGGATC
TCTTGTGGTTGGATGAAGAGGCCATGGAGCAAGGTGTGTCTGACTACATTAAGGGGCTCGGTGACGCATTTGGAA
CAGGTTTCACTGATGCTGTATCCAGGGAAGTTGAAGCCCTCAGGAACCACCTCATAGGATCTGATGGAGCAGTGG
AAAAAATCCTAAAGAACCTTATTAAGCTGATTTCAGCGTTAGTAATTGTGATTAGGAGCGATTATGATATGGTCA
CCCTCACAGCAACTTTAGCCCTGATTGGTTGTCATGGAAGTCCCTGGGCTTGGATTAAAGCCAAAACAGCATCCA
TTTTAGGTATCCCCATCGCCCAGAAGCAGAGCGCTTCTTGGCTAAAGAAATTTAATGATATGGCGAGTGCTGCCA
AGGGTTTAGAATGGATATCCAACAAAATTAGTAAGTTCATTGACTGGCTCAGGGAGAAGATTGTTCCAGCAGCTA
AAGAGAAAGCAGAATTTTTAACCAATTTGAAGCAATTACCACTATTAGAGAACCAGATCACGAGCTTGGAGCAGT
CCGCTGCCTCGCAAGAGGACCTTGAAGCTATGTTTGGGAATGTGTCATACCTCGCCCATTTCTGTCGCAAGTTCC
AACCATTATACGCCACGGAGGCCAAGCGAGTCTATGTTCTAGAGAAGAGAATGAACAATTACATGCAGTTCAAGA
GCAAACACCGTATTGAACCTGTATGTCTCATCATTAGAGGCTCACCAGGCACTGGAAAGTCCCTTGCGACCGGCA
TCATTGCCCGGGCCATAGCAGACAAGTACCACTCTAGTGTGTACTCACTCCCACCGGATCCTGACCATTTTGACG
GGTACAAACAGCAAGTGGTTACAGTTATGGATGACCTGTGCCAGAATCCTGATGGCAAAGACATGTCATTATTTT
GCCAGATGGTATCCACCGTGGATTTTATTCCACCAATGGCTTCTCTCGAAGAAAAGGGAGTTTCTTTCACATCTA
AATTTGTTATCGCATCCACCAACGCCAGCAACATTATAGTGCCCACAGTGTCTGACTCTGACGCCATTCGTCGCA
GGTTCTACATGGATTGCGACATTGAGGTCACAGACTCATACAAAACAGACTTGGGTAGACTAGACGCTGGGCGGG
CTGCTAAGTTATGCTCTGAAAACAACACCGCAAATTTCAAACGATGCAGCCCACTAGTGTGTGGGAAAGCTATTC
AACTTAGAGACAGGAAATCCAAGGTCAGGTATAGCGTGGACACAGTGGTCTCTGAACTTATTAGAGAATACAATA
GCAGATCCGCTATTGGTAACACAATTGAAGCATTATTCCAAGGCCCACCCAAGTTCAGGCCAATAAGGATCAGTC
TTGAGGAGAAGCCAGCCCCAGACGCTATTAGCGATCTCCTTGCTAGTGTGGATAGCGAGGAAGTGCGCCAATACT
GTAGGGAACAAGGCTGGATTATCCCTGAAACTCCCACCAATGTTGAACGACATCTTAATAGAGCAGTGCTAGTCG
TGCAATCCATCACTACTGTGGTGGCAGTCGTCTCACTGGTGTACGTCATTTACAAGCTCTTTGCGGGGTTTCAAG
GTGCGTATTCTGGAGCTCCCAAGCAAGTGCTCAAGAAGCCTGTCCTCCGCACGGCAACAGTGCAGGGTCCAAGCC
TTGATTTTGCCCTATCCTTGCTGAGGAGGAACATCAGGCAAGTCCAAACAGACCAAGGGCATTTTACCATGTTGG
GTGTCAGGGATCGCCTGGCTGTTCTCCCGCGGCACTCACAGCCCGGGAAGACTATTTGGGTGGAACACAAACTTG
TGAACATCCTTGATGCAGTCGAGCTGGTGGACGAGCAGGGCGTTAATTTGGAACTCACATTGGTGACACTAGATA
TTAATGAAAAATTTAGAGATATCACCAAGTTCATTCCAGAGACCATTAGCGGCGCTAGTGATGCAACTCTAGTGA
TCAACACAGAACATATGCCGTCAATGTTTGTCCCTGTGGGGGACGTCGTGCAGTACGGGTTCTTGAACCTCAGTG
GAAAGCCAACACATAGGACCATGATGTACAATTTCCCTACAAAAGCAGGACAGTGTGGAGGCGTGGTTACATCAG
TCGGTAAGATTGTTGGTATTCACATTGGTGGCAACGGGCGCCAAGGGTTCTGCGCTGGTTTGAAGAGGAGCTACT
TTGCGAGTATGCAAGGTGAGATCCAATGGGTGAAGCCTAACAAGGAAACTGGCAGACTAAACATCAATGGACCAA

CTCGCACTAAGTTGGAGCCTAGTGTATTTCATGATGTGTTTGAAGGCAACAAGGAACCAGCAGTTTTAACAAGTA
AAGACCCTAGATTGGAGGTCGACTTTGAACAAGCCCTGTTTTCCAAGTATGTGGGCAATGTTTTACACGAGCCCG
ATGAATATGTGACTCAAGCTGCCCTCCACTATGCGAATCAACTTAAACAATTGGACATAAACACTAGCAAGATGA
GCATGGAGGAAGCGTGCTATGGCACTGAAAACCTGGAAGCAATAGACCTCTGCACTAGTGCTGGGTATCCATACA
GTGCCCTTGGTATCAAGAAAAGAGACATTCTCGACCCCATAACCAGGGATGTGTCTAAGATGAAATTCTACATGG
ATAAATACGGACTAGATCTGCCATACTCTACCTATGTGAAGGATGAACTTAGATCTCTGGATAAAATCAAGAAAG
GAAAGTCACGCCTGATAGAGGCCAGCAGCTTGAATGACTCTGTCTACCTCAGAATGACTTTTGGGCACCTTTACG
AGGTGTTTCATGCTAACCCTGGTACTGTGACTGGCTCAGCAGTAGGTTGCAACCCAGACGTGTTTTGGAGTAAAC
TACCGATTCTGCTGCCTGGGTCACTCTTTGCCTTTGACTACTCAGGATATGATGCTAGTCTCAGCCCGGTATGGT
TCAGGGCTCTAGAAGTTGTGTTACGGGAGATTGGGTATTCAGAGGAGGCCGTGTCCCTAATAGAAGGAATCAACC
ACACCCACCATGTGTACCGGAATAAAACATACTGTGTACTTGGTGGGATGCCCTCAGGGTGCTCTGGTACTTCCA
TCTTCAATTCAATGATCAACAACATCATCATTAGAACCCTTTTGATCAAAACCTTTAAGGGAATAGACCTGGATG
AGTTGAACATGGTGGCCTATGGGGACGATGTGCTGGCCAGTTACCCTTTTCCTATTGATTGCCTTGAATTGGCTA
AGACTGGCAAAGAGTATGGTTTGACCATGACTCCTGCAGACAAATCACCCTGTTTCAATGAAGTAACATGGGAGA
ATGCTACCTTCCTGAAGAGAGGGTTCTTGCCAGACCACCAATTTCCATTCTTAATTCACCCTACGATGCCCATGA
GAGAGATCCATGAGTCCATTCGATGGACTAAGGACGCGCGTAACACCCAGGATCACGTGCGCTCCCTGTGTCTAT
TGGCATGGCACAATGGTAAGGATGAATATGAAAAGTTTGTGAGTGCAATTAGATCAGTTCCAGTTGGAAAAGCGT
TGGCCATTCCTAACTTTGAGAGTCTGAGAAGAAATTGGCTCGAATTGTTTTAATATTACAGTTTAAAGCTGAACC
CCACTAGAAATCTGGTCGTGTTAATGACTAGTGGGGGTAAATTTGTTATAACCGGAATAGC

>EV-A71/VM
TTAAAACAGCTGTGGGTTGTACCCACTCACAGGGCCCACGTGGCGCTAGCACTCTGGTTCTGCGGAACCTTTGTG
CGCCTGTTTTACGCCCCCCCCCCAATTTGCAACTTAGAAGCAATACACAACACTGATCAACAGCAGGCATGGCGC
ACCAGCTATGTCTTGATCAAGCACTTCTGTTTCCCCGGGCCGAGTATCAATAGACTGTTCACGCGGTTGAAGGAG
AAAGCGCCCGTTATCCGGCTAACTACTTCGAGAAACCTAGTAGCACCATTGAAGCTGCAGAGTGTTTCGCTCGGC
ACTTCCCCCGTGTAGATCAGGTCGATGAGTCACTGCAATCCCCACGGGCGACCGTGGCAGTGGCTGCGCTGGCGG
CCTGCCTATGGGGCAACCCATAGGACGCTCTAATGTGGACATGGTGCGAAGAGTCTATTGAGCTAGTTAGTAGTC
CTCCGGCCCCTGAATGCGGCTAATCCTAACTGTGGAGCACATGCCTTCAATCCAGAGGGTAGTGTGTCGTAATGG
GCAACTCTGCAGCGGAACCGACTACTTTGGGTGTCCGTGTTTCCTTTTATCTTTACATTGGCTGCTTATGGTGAC
GATTATAGAATTGTTACCATATAGCTATTGGATTGGCCATCCGGTGTGCAATAGAGCTATTATATACCTGTTTGT
TGGCTTTGTACCACTAACCTTAAAATCTATAACCACCCTCGATTTTATATTAACCCTCAATACAATCAAACATGG
GCTCACAGGTGTCTACTCAGCGATCCGGCTCCCACGAGAACTCCAATTCAGCTACAGAAGGCTCCACCATTAATT
ACACTACCATCAACTATTACAAAGACTCCTATGCTGCGACAGCGGGCAAACAGAGCCTCAAGCAAGACCCTGATA
AATTTGCTAACCCTGTCAAGGACATTTTCACTGAAATGGCTGCACCACTGAAGTCTCCATCCGCTGAGGCTTGTG
GTTACAGTGATCGCGTGGCACAACTCACCATTGGAAACTCCACCATCACTACACAGGAGGCGGCGAATATCATAG
TCGGTTATGGTGAGTGGCCCTCATACTGCTCTGATGACGATGCTACAGCGGTGGACAAGCCAACGCGCCCAGATG
TTTCAGTGAATAGGTTTTATACGTTGGATACTAAATTGTGGGAAAAGTCATCCAAGGGGTGGTATTGGAAGTTTC
CTGATGTACTGACTGAGACCGGAGTCTTTGGCCAGAATGCACAGTTTCACTATTTATATAGGTCAGGATTTTGCA
TTCATGTGCAATGTAATGCTAGCAAGTTCCATCAAGGAGCGTTGTTAGTCGCCATACTTCCAGAGTATGTTATAG
GGACAGTGGCAGGCGGCACAGGAACTGAGGACAGCCACCCTCCTTACAAACAAACACAACCTGGCGCCGATGGTT
TTGAGTTGCAGCACCCGTACGTACTCGATGCTGGGATTCCTATATCACAATTAACAGTGTGCCCCCACCAATGGA
TTAACCTACGGACCAATAACTGTGCCACAATAATAGTGCCGTATATGAACACACTGCCTTTCGACTCTGCCCTGA
ACCATTGCAACTTTGGGCTGTTGGTGGTGCCCATTAGCCCACTAGATTTTGACCAAGGGGCAACTCCGGTTATCC
CTATTACAATCACTCTAGCTCCAATGTGCTCTGAGTTTGCAGGTCTCAGACAGGCGGTCACTCAAGGTTTTCCCA
CCGAGCCAAAACCAGGAACGAACCAATTTTTGACCACCGATGACGGTGTCTCAGCACCCATTTTACCAAATTTCT
ACCCCACACCATGTATTCACATACCCGGTGAAGTCAGAAACCTGCTTGAGTTGTGTCAAGTGGAGACCATTCTTG
AGGTTAACAATGTACCCACCAATGCCACCAGTCTGATGGAAAGGCTACGATTCCCGGTGTCCGCGCAAGCGGGAA
AAGGTGAATTGTGTGCCGTGTTTAGGGCCGACCCTGGAAGAGACGGTCCATGGCAATCAACAATGCTGGGCCAGT
TGTGTGGATATTACACCCAGTGGTCAGGATCACTGGAGGTTACTTTTATGTTCACCGGGTCTTTCATGGCCACGG
GTAAAATGCTCATAGCTTATACACCTCCTGGTGGCCCCTTACCCAGAGATCGGGCCACAGCAATGCTGGGCACAC
ATGTTATCTGGGATTTTGGGCTACAATCATCTGTCACCCTTGTAATACCATGGATTAGCAACACCCACTACAGAG
CGCATGCCCGGGATGGAGTGTTCGATTACTATACCACAGGACTGGTTAGTATCTGGTATCAAACAAACTACGTGG
TTCCAATTGGGGCACCCAACACAGCTTACATAATAGCACTAGCGGCAGCCCAGAAGAATTTTACCATGAAACTGT
GTAAAGACACCAGTCACATATTACAGACAGCCTCTATTCAGGGAGATAGAGTGGCAGATGTGATAGAGAGCTCTA
TAGGAGATAGTGTGAGTAGGGCACTTACCCAGGCCCTGCCAGCTCCAACAGGTCAGAATACGCAGGTGAGCAGTC
ATCGACTAGACACTGGTGAAGTTCCAGCGCTCCAAGCTGCTGAAATAGGGGCATCGTCAAATACTAGTGATGAGA
GTATGATTGAGACACGATGCGTTCTTAATTCACACAGTACGGCAGAGACCACCCTGGACAGCTTCTTCAGTAGGG
CAGGCTTGGTAGGAGAGATAGATCTCCCTATTGAGGGTACCACTAATCCAAATGGTTATGCTAATTGGGATATAG
ACATAACTGGTTACGCACAAATGCGCAGGAAAGTGGAGCTGTTCACCTACATGCGCTTTGATGCGGAATTCACTT
TTATTGCGTGCACTCCTACTGGTCAGGTTGTCCCACAATTACTTCAGTATATGTTTGTTCCCCCTGGTGCTCCCA
AACCAGAGTCTAGAGAATCACTTGCTTGGCAGACAGCCACAAACCCCTCAGTTTTTGTCAAGTTGACTGATCCCC

CGGCACAGGTCTCAGTTCCGTTCATGTCACCCGCGAGCGCTTACCAGTGGTTTTACGACGGGTACCCCACGTTTG
GAGAACACAAACAGGAGAAAGACCTTGAGTATGGAGCGTGCCCTAATAATATGATGGGCACTTTCTCGGTGCGAA
CTGTGGGTTCATCAAAGTCCAAGTATTCTTTGGTTGTCAGGATATATATGAGAATGAAGCATGTCAGGGCGTGGA
TACCTCGCCCGATGCGCAACCAAAACTACCTGTTTAAAGCCAATCCAAACTATGCCGGTAACTCCATCAAACCGA
CCGGCACTAGTCGTACTGCCATTACTACCCTTGGAAAGTTCGGCCAGCAATCTGGGGCCATCTACGTGGGCAACT
TCAGAGTGGTTAATCGTCACCTCGCTACTCATAATGACTGGGCGAACCTCGTCTGGGAAGATAGCTCCCGCGACC
TATTAGTGTCGTCTACCACCGCCCAGGGCTGTGATACAATTGCACGTTGTGACTGTCAAACAGGAGTGTACTATT
GTAATTCCAAAAGAAAGCACTATCCAGTCAGCTTCTCCAAACCCAGCCTCATATATGTGGAGGCTAGCGAGTATT
ACCCTGCTAGATACCAATCGCACCTGATGCTTGCAGCAGGCCACTCTGAGCCCGGCGACTGCGGGGGCATCTTAA
GGTGTCAACATGGTGTAGTTGGTATAGTGTCCACGGGTGGCAACGGGCTTGTTGGTTTTGCTGATGTGAGGGATC
TCTTGTGGTTGGATGAAGAGGCCATGGAGCAAGGTGTGTCTGACTACATTAAGGGGCTCGGTGACGCATTCGGAA
CAGGTTTCACTGATGCTGTATCCAGGGAAGTTGAAGCCCTCAGGAACCACCTCATAGGATCTGATGGAGCAGTGG
AAAAAATCCTAAAGAACCTTATTAAGCTGATTTCAGCGTTAGTAATTGTGATTAGGAGCGATTATGATATGGTCA
CCCTCACAGCAACTTTAGCCCTGATTGGTTGTCATGGAAGTCCCTGGGCTTGGATTAAAGCCAAAACAGCATCCA
TTTTAGGTATCCCCATCGCCCAGAAGCAGAGCGCTTCTTGGCTAAAGAAATTTAATGATATGGCGAGTGCTGCCA
AGGGTTTAGAATGGATATCCAACAAAATTAGTAAGTTCATTGACTGGCTCAGGGAGAAGATTGTTCCAGCAGCTA
AAGAGAAAGCAGAATTTTTAACCAATTTGAAGCAATTACCACTATTAGAGAACCAGATCACGAACTTGGAGCAGT
CCGCTGCCTCGCAAGAGGACCTTGAAGCTATGTTTGGGAATGTGTCATACCTCGCCCATTTCTGTCGCAAGTTCC
AACCATTATACGCCACGGAGGCCAAGCGAGTCTATGTTCTAGAGAAGAGAATGAACAATTACATGCAGTTCAAGA
GCAAACACCGTATTGAACCTGTATGTCTCATCATTAGAGGCTCACCAGGCACTGGAAAGTCCATTGCGACCGGCA
TCATTGCCCGGGCCATAGCAGACAAGTACCACTCTAGTGTGTACTCACTCCCACCGGATCCTGACCATTTTGACG
GGTACAAACAGCAAGTGGTTACAGTTATGGATGACCTGTGCCAGAATCCTGACGGCAAAGACATGTCATTATTTT
GCCAGATGGTATCCACCGTGGATTTTATTCCACCAATGGCTTCTCTCGAAGAAAAGGGAGTTTCTTTCACATCTA
AATTTGTTATCGCATCCACCAACGCCAGCAACATTATAGTGCCCACAGTGTCTGACTCTGACGCCATTCGTCGCA
GGTTCTACATGGATTGCGACATTGAGGTCACAGACTCATACAAAACAGACTTGGGTAGACTAGACGCTGGGCGGG
CTGCTAAGTTATGCTCTGAAAACAACACTGCAAACTTCAAACGATGCAGCCCACTAGTGTGTGGGAAAGCTATTC
AACTTAGAGACAGGAAATCCAAGGTCAGGTATAGCGTGGACACAGTGGTCTCTGAACTTATTAGAGAATACAACA
GCAGATCCGCTATTGGTAACACAATTGAAGCATTATTCCAAGGCCCACCCAAGTTCAGGCCAATAAGGATCAGTC
TTGAGGAGAAGCCAGCCCCAGACGCTATTAGCGATCTCCTTGCTAGTGTGGATAGCGAGGAAGTGCGCCAATACT
GTAGGGAACAAGGCTGGATTATCCCTGAAACTCCCACCAATGTTGAACGACATCTTAATAGAGCAGTGCTAGTCG
TGCAATCCATCACTACTGTGGTGGCAGTCGTCTCACTGGTGTACGTCATTTACAAGCTCTTTGCGGGGTTTCAAG
GTGCGTATTCTGGAGCTCCCAAGCAAGTGCTCAAGAGGCCTGTCCTCCGCACGGCAACAGTGCAGGGTCCAAGCC
TTGATTTTGCCCTATCCTTGCTGAGGAGGAACATCAGGCAAGTCCAAACAGACCAAGGGCATTTTACCATGTTGG
GTGTCAGGGATCGCCTGGCTGTTCTCCCGCGGCACTCACAACCCGGGAAGACTATTTGGGTGGAACACAAACTTG
TGAACATCCTTGATGCAGTCGAGCTGGTGGACGAGCAGGGCGTTAATTTGGAACTCACATTGGTGACACTAGATA
TTAATGAAAAATTTAGAGATATCACCAAGTTCATTCCAGAGACCATTAGCGGCGCTAGTGATGCAACTCTAGTGA
TCAACACAGAACATATGCCGTCAATGTTTGTCCCTGTGGGGGACGTCGTGCAGTACGGGTTCTTGAACCTCAGTG
GAAAGCCAACACATAGGACCATGATGTACAATTTCCCTACAAAAGCAGGACAGTGTGGAGGCGTGGTTACATCAG
TCGGTAAGATTGTTGGTATTCACATTGGTGGCAACGGGCGCCAAGGGTTCTGCGCTGGTTTGAAGAGGAGCTACT
TTGCGAGTATGCAAGGTGAGATCCAATGGGTGAAGCCTAACAAGGAAACTGGCAGACTGAACATCAATGGACCAA
CTCGCACTAAGTTGGAGCCTAGTGTGTTTCATGATGTGTTTGAAGGCAACAAGGAACCAGCAGTTTTAACAAGTA
AAGACCCTAGATTGGAGGTCGACTTTGAACAAGCCCTGTTTTCCAAGTATGTGGGCAATGTTTTACACGAGCCCG
ATGAATATGTGACTCAAGCTGCCCTCCACTATGCGAATCAACTTAAACAATTGGACATAAACACTAGCAAGATGA
GCATGGAGGAAGCGTGCTATGGCACTGAAAACCTGGAAGCAATAGACCTCTGCACTAGTGCTGGGTATCCATACA
GTGCCCTTGGTATCAAGAAAAGAGACATTCTCGACCCCATAACCAGGGATGTGTCTAAGATGAAATTCTACATGG
ATAAATACGGACTAGATCTGCCATACTCTACCTATGTGAAGGATGAACTTAGATCTCTGGATAAAATCAAGAAAG
GAAAGTCACGCCTGATAGAGGCCAGCAGCTTGAATGACTCTGTCTACCTCAGAATGACTTTTGGGCACCTTTACG
AGGTGTTTCATGCTAACCCTGGTACTGTGACTGGCTCAGCAGTAGGTTGCAACCCAGACGTGTTTTGGAGTAAAC
TACCGATTCTGCTGCCTGGGTCACTCTTTGCCTTTGACTACTCAGGATATGATGCTAGTCTCAGCCCGGTATGGT
TCAGGGCTCTAGAAGTTGTGTTACGGGAGATTGGGTATTCAGAGGAGGCCGTGTCCCTAATAGAAGGAATCAACC
ACACCCACCATGTGTACCGGAATAAAACATACTGTGTACTTGGTGGGATGCCCTCAGGGTGCTCTGGTACTTCCA
TCTTCAATTCAATGATCAACAACATCATCATTAGAACCCTTTTGATCAAAACCTTTAAGGGAATAGACCTGGATG
AGTTGAACATGGTGGCCTATGGGGACGATGTGCTGGCCAGTTACCCTTTTCCTATTGATTGCCTTGAATTGGCTA
AGACTGGCAAAGAGTATGGTTTGACCATGACTCCTGCAGACAAATCACCCTGTTTCAATGAAGTAACATGGGAGA
ATGCTACCTTCCTGAAGAGAGGGTTCTTGCCAGACCACCAATTTCCATTCTTAATTCACCCTACGATGCCCATGA
GAGAGATCCATGAGTCCATTCGATGGACTAAGGACGCGCGTAACACCCAGGATCACGTGCGCTCCCTGTGTCTAT
TGGCATGGCACAATGGTAAGGATGAATATGAAAAGTTTGTGAGTGCAATTAGATCAGTTCCAGTTGGAAAAGCGT
TGGCCATTCCTAACTTTGAGAGTCTGAGAAGAAATTGGCTCGAATTGTTTTAATATTACAGTTTAAAGCTGAACC
CCACTAGAAATCTGGTCGTGTTAATGACTAGTGGGGGTAAATTTGTTATAACCGGAATAGC

Table S5: Synonymous and non-synonymous mutations of plaque variants in comparison with the EV-A71 strain 41 subgenotype B4 (GenBank: AF316321.2).

**Variants**	**Mutation (n.t. changes)**	**Regions in EV-A71 genome**	**Amino acid changes**
HP	A99G	5'UTR	-
MP	U209C	5'UTR	-
SP,MP	U290C	5'UTR	-
VS	C1711U	VP2-253	Non-synonymous mutation [Thr(T)→Ile(I)] ACU→AUU
VM	U1748C	VP3-11	Synonymous mutation [Asn(N)]
BP,HP,VS,VM	C1800U	VP3-29	Non-synonymous mutation [His(H)→Tyr(Y)] CAC→UAC
VS,VM	A2146G	VP3-144	Non-synonymous mutation [Lys(K)→Arg(R)] AAA→AGA
MP	U2271C	VP3-186	Non-synonymous mutation [Phe(F)→Leu(L)] UUC→CUC
SP	U2528C	VP1-29	Synonymous mutation [Gly(G)]
VS,VM	C2534U	VP1-31	Synonymous mutation [Asn(N)]
VS,VM	C2730A	VP1-97	Non-synonymous mutation [Leu(L)→Ile(I)] CUU→AUU
SP,MP	A2752G	VP1-104	Non-synonymous mutation [Asn(N)→Ser(S)] AAU→AGU
VS,VM	G2853A	VP1-138	Non-synonymous mutation [Val(V)→Ile(I)] GUU→AUU
BP,HP	U2989C	VP1-183	Non-synonymous mutation [Leu(L)→Ser(S)] UUG→UCG

SP, MP, BP, HP	C3151A	VP1-237	Non-synonymous mutation [Thr(T)→Asn(N)] ACU→AAU
VS,VM	C3177U	VP1-246	Non-synonymous mutation [Pro(P)→Ser(S)] CCU→UCU
SP,MP	A3285G	VP1-282	Non-synonymous mutation [Asn(N)→Asp(D)] AAC→GAC
VS	C3671U	2A-113	Synonymous mutation [Ile(I)]
VS,VM	C3725U	2A-131	Synonymous mutation [Leu(L)]
VM	U3821C	2B-13	Synonymous mutation [Phe(F)]
BP,HP	U4109A	2C-10	Non-synonymous mutation [Asp(D)→Glu(E)] GAU→GAA
SP	G4156C	2C-26	Non-synonymous mutation [Ser(S)→Thr(T)] AGU→ACU
VS	A4264G	2C-62	Non-synonymous mutation [Asn(N)→Ser(S)] AAC→AGC
SP	G4367A	2C-96	Synonymous mutation [Thr(T)]
VM	C4488A	2C-137	Non-synonymous mutation [Leu(L)→Ile(I)] CUU→AUU
VS	C4628U	2C-183	Synonymous mutation [Asp(D)]
BP,HP	U4712C	2C-211	Synonymous mutation [Val(V)]
VM	C4904U	2C-275	Synonymous mutation [Thr(T)]
VM	U4910C	2C-277	Synonymous mutation [Asn(N)]
VM	U5024C	2C-315	Synonymous mutation [Asn(N)]
BP,HP	C5297U	3A-77	Synonymous mutation [Val(V)]
BP,HP	A5362C	3B-13	Non-synonymous mutation [Lys(K)→Thr(T)] AAG→ACG
VM	A5362G	3B-13	Non-synonymous mutation [Lys(K)→Arg(R)] AAG→AGG

BP,HP	G5429A	3C-36	Synonymous mutation [Gln(Q)]
VM	G5516A	3C-41	Synonymous mutation [Leu(L)]
VM	A5984G	3D-15	Synonymous mutation [Val(V)]
VM	A6026G	3D-29	Synonymous mutation [Arg(R)]
HP	C6050U	3D-37	Synonymous mutation [Asp(D)]
BP,HP	A6140G	3D-67	Synonymous mutation [Leu(L)]
SP	U6621C	3D-228	Non-synonymous mutation [Ser(S)→Pro(P)] AAG→ACG
BP	C6872U	3D-311	Synonymous mutation [Ile(I)]
BP,HP	C7187U	3D-416	Synonymous mutation [Arg(R)]
VS	A7297G	3D-453	Non-synonymous mutation [Asn(N)→Ser(S)] AAU→AGU

Table S6: Summary of salt bridge interactions in 3D polymerase of EV-A71/WT and EV-A71/SP.

Protein	Amino acids		EV-A71/WT	EV-A71/SP
	Residue 1	Residue 2	Distance (Å)	
3D	ND1 HIS A 31	OD1 ASP A 32	2.88	2.93
	NZ LYS A 38	OE1 GLU A 397	3.17	3.08
	NH1 ARG A 49	OD1 ASP A 47	3.89	3.86
	NH1 ARG A 49	OD2 ASP A 47	2.95	2.92
	NH2 ARG A 49	OE1 GLU A 39	3.02	2.94
	NZ LYS A 61	OE1 GLU A 177	-	2.94
	ND1 HIS A 80	OD1 ASP A 320	-	2.93
	ND1 HIS A 80	OD2 ASP A 320	-	3.78
	NZ LYS A 94	OE1 GLU A 261	2.83	-
	NH1 ARG A 128	OD1 ASP A 132	3.80	3.53
	NH1 ARG A 128	OD2 ASP A 132	2.92	3.05
	NH2 ARG A 128	OD2 ASP A 132	3.05	3.14
	NZ LYS A 142	OD1 ASP A 146	2.98	2.88
	NZ LYS A 142	OD2 ASP A 146	-	3.82
	NH1 ARG A 163	OE1 GLU A 161	3.83	-
	NH2 ARG A 163	OE1 GLU A 161	3.86	-
	NZ LYS A 170	OE1 GLU A 51	-	2.83
	NH1 ARG A 174	OD2 ASP A 238	-	2.93
	NH2 ARG A 174	OD2 ASP A 238	-	2.89
	ND1 HIS A 193	OE1 GLU A 196	2.89	2.90

ND1 HIS A 199	OE1 GLU A 108	-	3.68
NE2 HIS A 199	OE1 GLU A 108	-	3.94
NH2 ARG A 247	OE1 GLU A 250	3.27	3.13
ND1 HIS A 274	OE1 GLU A 177	2.82	-
NZ LYS A 279	OE1 GLU A 55	-	2.90
NZ LYS A 312	OD2 ASP A 71	3.46	3.08
NZ LYS A 312	OE1 GLU A 350	2.99	3.70
NZ LYS A 346	OE1 GLU A 343	3.24	3.09
NZ LYS A 360	OD2 ASP A 233	2.84	2.85
NZ LYS A 376	OE1 GLU A 397	3.13	2.97
NH1 ARG A 377	OD1 ASP A 215	-	4.00
NH2 ARG A 377	OD1 ASP A 215	2.97	2.90
NE2 HIS A 390	OD1 ASP A 382	2.92	2.91
NH1 ARG A 396	OE1 GLU A 35	-	3.83
NH2 ARG A 396	OE1 GLU A 35	-	2.92
NH2 ARG A 403	OE1 GLU A 400	3.49	3.01
NZ LYS A 406	OD2 ASP A 160	-	3.42
NH1 ARG A 409	OD1 ASP A 407	-	3.74
NH1 ARG A 409	OD2 ASP A 407	2.84	2.97
NH1 ARG A 416	OD1 ASP A 413	2.86	2.86
NE2 HIS A 424	OD1 ASP A 215	3.81	3.81
NH1 ARG A 455	OD1 ASP A 382	3.44	3.35
NH1 ARG A 455	OD2 ASP A 382	2.92	2.86

The ESBRI software predicts salt bridges between the charged residues (Arg, Lys, His, Asp, and Glu) with a distance ≤ 4.0 Å.

“-” indicates no salt bridges or salt bridges with a distance above 4.0 Å.

Data S7: Sequences of human SCARB2 (PDB: 6I2K) and African green monkey SCARB2 (GenBank: XP_007997136.2) with its sequence comparisons.

```
>XP_007997136.2 (mSCARB2)
MGRCCFYTAGTLSLLLLVTSVTLLVARVFQKAVDQSIEKKIVLRNGTEAFDSWEKPPLPVYTQFYFFNVT
NPEEILRGETPRVEEVGPYTYRELRNKANVQFGDNGTTISAVSNKAYVFERDQSVGDPKIDLIRTLNIPV
LTVIEWSQVHFLREIIEAMLKAYQQKLFVTHTVDELLWGYKDEILSLIHVFRPDISPYFGLFYEKNGTND
GDYVFLTGEDNYLNFTKIVEWNGKTSLDWWITDKCNMINGTDGDSFHPLITKDEVLYVFPSDFCRSVYIT
FSDYESVQGLPAFRYKVPAEILANTSDNAGFCIPEGNCLGSGVLNVSICKNGAPIIMSFPHFYQADERFV
SAIEGMHPNKEDHETFVDINPLTGIILKAAKRFQINIYVKKLDDFVETGDIRTMVFPVMYLNESVLIDKE
TASRLKSVINTTLIITNIPYIIMALGVFFGLVFTWLACKGQGSMDEGTADERAPLIRT

>6I2K (hSCARB2)
ETGVFQKAVDQSIEKKIVLRNGTEAFDSWEKPPLPVYTQFYFFNVTNPEEILRGETPRVEEVGPYTYREL
RNKANIQFGDNGTTISAVSNKAYVFERDQSVGDPKIDLIRTLNIPVLTVIEWSQVHFLREIIEAMLKAYQ
QKLFVTHTVDELLWGYKDEILSLIHVFRPDISPYFGLFYEKNGTNDGDYVFLTGEDSYLNFTKIVEWNGK
TSLDWWITDKCNMINGTDGDSFHPLITKDEVLYVFPSDFCRSVYITFSDYESVQGLPAFRYKVPAEILAN
TSDNAGFCIPEGNCLGSGVLNVSICKNGAPIIMSFPHFYQADERFVSAIEGMHPNQEDHETFVDINPLTG
IILKAAKRFQINIYVKKLDDFVETGDIRTMVFPVMYLNESVHIDKETASRLKSMINTTGKHHHHHH
```

```
hSCARB2      ------------------------ETGVFQKAVDQSIEKKIVLRNGTEAFDSWEKPPLPV   36
mSCARB2      MGRCCFYTAGTLSLLLLVTSVTLLVARVFQKAVDQSIEKKIVLRNGTEAFDSWEKPPLPV   60
                                      : *********************************

hSCARB2      YTQFYFFNVTNPEEILRGETPRVEEVGPYTYRELRNKANIQFGDNGTTISAVSNKAYVFE   96
mSCARB2      YTQFYFFNVTNPEEILRGETPRVEEVGPYTYRELRNKANVQFGDNGTTISAVSNKAYVFE   120
             ***************************************:********************

hSCARB2      RDQSVGDPKIDLIRTLNIPVLTVIEWSQVHFLREIIEAMLKAYQQKLFVTHTVDELLWGY   156
mSCARB2      RDQSVGDPKIDLIRTLNIPVLTVIEWSQVHFLREIIEAMLKAYQQKLFVTHTVDELLWGY   180
             ************************************************************

hSCARB2      KDEILSLIHVFRPDISPYFGLFYEKNGTNDGDYVFLTGEDSYLNFTKIVEWNGKTSLDWW   216
mSCARB2      KDEILSLIHVFRPDISPYFGLFYEKNGTNDGDYVFLTGEDNYLNFTKIVEWNGKTSLDWW   240
             ****************************************.*******************

hSCARB2      ITDKCNMINGTDGDSFHPLITKDEVLYVFPSDFCRSVYITFSDYESVQGLPAFRYKVPAE   276
mSCARB2      ITDKCNMINGTDGDSFHPLITKDEVLYVFPSDFCRSVYITFSDYESVQGLPAFRYKVPAE   300
             ************************************************************

hSCARB2      ILANTSDNAGFCIPEGNCLGSGVLNVSICKNGAPIIMSFPHFYQADERFVSAIEGMHPNQ   336
mSCARB2      ILANTSDNAGFCIPEGNCLGSGVLNVSICKNGAPIIMSFPHFYQADERFVSAIEGMHPNK   360
             ***********************************************************:

hSCARB2      EDHETFVDINPLTGIILKAAKRFQINIYVKKLDDFVETGDIRTMVFPVMYLNESVHIDKE   396
mSCARB2      EDHETFVDINPLTGIILKAAKRFQINIYVKKLDDFVETGDIRTMVFPVMYLNESVLIDKE   420
             ******************************************************* ****

hSCARB2      TASRLKSMINTTGKHHHHHH--------------------------------------   416
mSCARB2      TASRLKSVINTTLIITNIPYIIMALGVFFGLVFTWLACKGQGSMDEGTADERAPLIRT   478
             *******:****    :  :
```
